# Transcriptional Dysregulation of *MYC* Reveals Common Enhancer-Docking Mechanism

**DOI:** 10.1016/j.celrep.2018.03.056

**Published:** 2018-04-10

**Authors:** Jurian Schuijers, John Colonnese Manteiga, Abraham Selby Weintraub, Daniel Sindt Day, Alicia Viridiana Zamudio, Denes Hnisz, Tong Ihn Lee, Richard Allen Young

**Affiliations:** 1 Whitehead Institute for Biomedical Research, 455 Main Street, Cambridge, MA 02142, USA; 2 Department of Biology, Massachusetts Institute of Technology, Cambridge, MA, 02139, USA

## Abstract

Transcriptional dysregulation of the *MYC* oncogene is among the most frequent events in aggressive tumor cells, and this is generally accomplished by acquisition of a super-enhancer somewhere within the 2.8 Mb TAD where *MYC* resides. We find that these diverse cancer-specific super-enhancers, differing in size and location, interact with the *MYC* gene through a common and conserved CTCF binding site located 2 kb upstream of the *MYC* promoter. Genetic perturbation of this enhancer-docking site in tumor cells reduces CTCF binding, super-enhancer interaction, *MYC* gene expression, and cell proliferation. CTCF binding is highly sensitive to DNA methylation, and this enhancer-docking site, which is hypomethylated in diverse cancers, can be inactivated through epigenetic editing with dCas9-DNMT. Similar enhancer-docking sites occur at other genes, including genes with prominent roles in multiple cancers, suggesting a mechanism by which tumor cell oncogenes can generally hijack enhancers. These results provide insights into mechanisms that allow a single target gene to be regulated by diverse enhancer elements in different cell types.

## In Brief

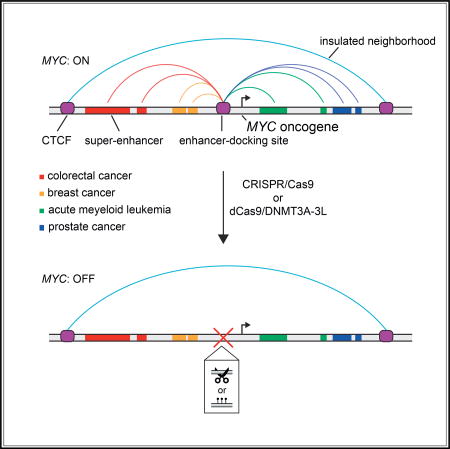

Schuijers et al. show that a conserved CTCF site at the promoter of the *MYC* oncogene plays an important role in enhancer-promoter looping with tumor-specific super-enhancers. Perturbation of this site provides a potential therapeutic vulnerability.

## INTRODUCTION

Elevated expression of the c-MYC transcription factor occurs in a broad spectrum of human cancers and is associated with tumor aggression and poor clinical outcome (Berns et al., 1992; Dang, 2012; Gabay et al., 2014; Grotzer et al., 2001). Activation of the *MYC* gene, which encodes c-MYC, is a hallmark of cancer initiation and maintenance. Dysregulation of *MYC* is often achieved through the formation of large tumor-specific super-enhancers in the region surrounding the *MYC* gene (Chapuy et al., 2013; Fulco et al., 2016; Herranz et al., 2014; Hnisz et al., 2013; Lin et al., 2016; Liu et al., 2015; Lovén et al., 2013; Shi et al., 2013; Whyte et al., 2013; Zhang et al., 2016). These large enhancer clusters differ in size, composition, and distance from the *MYC* promoter, yet all accomplish the same task of stimulating *MYC* overexpression across a broad spectrum of tumors.

Selective gene activation is essential to the gene expression programs that define both normal and cancer cells. During gene activation, transcription factors (TFs) bind enhancer elements and regulate transcription from the promoters of nearby or distant genes through physical contacts that involve looping of DNA between enhancers and promoters (Bonev and Cavalli, 2016; Buecker and Wysocka, 2012; Bulger and Groudine, 2011; Fraser et al., 2015; Müeller-Storm et al., 1989; Spitz, 2016; de Wit et al., 2013). The mechanisms that ensure that specific enhancers interact with specific promoters are not fully understood. Some enhancer-promoter interactions are likely determined by the nature of TFs bound at the two sites (Muerdter and Stark, 2016; Weintraub et al., 2017).

Recent studies have revealed that specific chromosome structures play important roles in gene control. Enhancer-promoter interactions generally occur within larger chromosomal loop structures formed by the interaction of CTCF proteins bound to each of the loop anchors (Dekker and Mirny, 2016; Fraser et al., 2015; Gibcus and Dekker, 2013; Gorkin et al., 2014a; Hnisz et al., 2016a, 2018; Ji et al., 2016). These loop structures, variously called topologically associated domains (TADs), sub-TADs, loop domains, CTCF contact domains, and insulated neighborhoods, tend to insulate enhancers and genes within the CTCF-CTCF loops from elements outside those loops (Dixon et al., 2012; Dowen et al., 2014; Franke et al., 2016; Hnisz et al., 2016a, 2016b; Ji et al., 2016; Narendra et al., 2015; Nora et al., 2012; Phillips-Cremins et al., 2013; Rao et al., 2014). Constraining DNA interactions within CTCF-CTCF loop structures in this manner may facilitate proper enhancer-promoter contacts.

CTCF does not generally occupy enhancer and promoter elements (Cuddapah et al., 2009; Dixon et al., 2012; Dowen et al., 2014; Handoko et al., 2011; Ji et al., 2016; Kim et al., 2007; Phillips-Cremins et al., 2013; Rao et al., 2014; Rubio et al., 2008; Tang et al., 2015). Another TF, YY1, generally binds to enhancers and promoters and facilitates their interaction through YY1 dimerization (Weintraub et al., 2017). However, when CTCF does bind these regulatory elements, it can also contribute to enhancer-promoter interactions (Banani et al., 2017; Nora et al., 2017; Splinter et al., 2006; Zuin et al., 2014).

Here, we investigate DNA looping structures in the *MYC* locus in multiple cancers and identify a CTCF-occupied site at the *MYC* promoter that facilitates docking with essentially any enhancers that are formed within the 2.8 Mb *MYC* locus. The CTCF-occupied site at the *MYC* promoter, which we call the *MYC* enhancer-docking site, can be abrogated by genetic and epigenetic editing. Similar enhancer-docking sites occur at other oncogenes. This suggests a mechanism by which tumor cells can generally hijack enhancers and, with editing, a potential therapeutic vulnerability.

## RESULTS

### Cell-Type-Specific *MYC* Enhancers Loop to a Common Upstream CTCF Site

**Figure 1:**
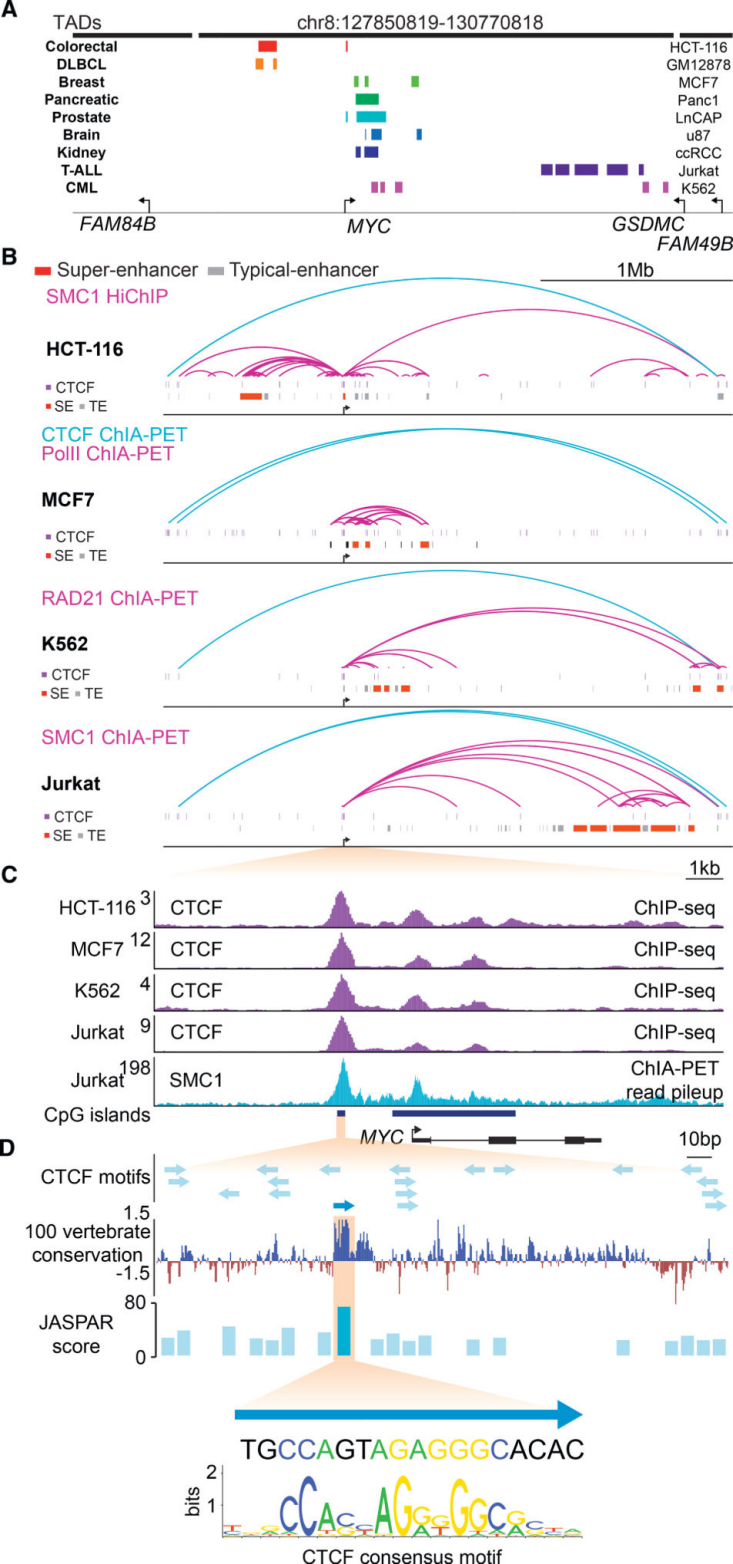
Cell-Type-Specific Super-Enhancers in the *MYC* Locus Loop to a Common CTCF Site (A) The 4.5 Mb region surrounding the *MYC* gene. The 2.8 Mb TAD containing *MYC* and portions of the two adjacent TADs are indicated with thick black horizontal lines. Super-enhancers (data from Becket et al., 2016; Frietze et al., 2012; Lin et al., 2012; Pope et al., 2014; Wang et al., 2011) are shown in colored boxes for a panel of tumor cell lines that express *MYC*. (B) Chromosome interaction data at the ~3 Mb *MYC* locus. For HCT-116, SMC1 HiChIP interactions with an origami score of at least 0.9 and a minimum PET count of 9 are shown as purple arcs; the insulated neighborhood spanning interaction, which encompasses the TAD, is shown as a blue arc (data from this study). For MCF7, Pol II ChIA-PET interactions with an origami score of at least 0.9 are shown as purple arcs; the insulated neighborhood spanning interactions from CTCF ChIA-PET data are shown in blue (data from ENCODE and Li et al., 2012). For K562, RAD21 ChIA-PET interactions with an origami score of at least 0.9 are shown as purple arcs; the insulated neighborhood spanning interaction is shown in blue and has an origami score of 0.44 (data from Heidari et al., 2014). For Jurkat, SMC1 ChIA-PET interactions with an origami score of at least 0.99 are shown as purple arcs; the insulated neighborhood spanning interactions are shown in blue (data from Hnisz et al., 2016a). CTCF ChIP-seq peaks are depicted as purple rectangles, super-enhancers are depicted as red rectangles, and typical enhancers are depicted as gray rectangles (data from this study; Hnisz et al., 2016a; Pope et al., 2014). (C) CTCF ChIP-seq and SMC1 ChIA-PET read counts in the *MYC* promoter regions. Purple tracks display CTCF ChIP-seq signal in the four cell lines from (B). Light blue track displays the read counts from read pileup of Jurkat SMC1 ChIA-PET data, showing that the major peak of SMC1 ChIA-PET reads occurs at the −2 kb CTCF site. Dark blue bars indicate CpG islands. ChIP-seq read counts are shown in reads per million sequenced reads per base pair. ChIA-PET reads are shown as read counts per base pair. (D) The top panel depicts all putative CTCF binding motifs as blue arrows, which indicate the orientation of the motif. The CTCF motif depicted in dark blue occurs in the most conserved region and shows the best match with consensus CTCF motif. 100 vertebrate conservation from the UCSC genome browser is depicted in the middle panel. The JASPAR score for all the motifs is indicated with blue bars. The position weight matrix for the canonical JASPAR CTCF motif and the actual sequence is shown below. See also Figure S1

Previous studies have established that tumor cells acquire tumor-specific super-enhancers at various sites throughout the *MYC* locus (Figures 1A and S1A) (Bradner et al., 2017; Chapuy et al., 2013; Gabay et al., 2014; Gröschel et al., 2014; Herranz et al., 2014; Hnisz et al., 2013; Lin et al., 2016; Lovén et al., 2013; Parker et al., 2013; Zhang et al., 2016; Shi et al., 2013), but the mechanisms by which these diverse enhancer structures control *MYC* are not clear. In one case, for example, a super-enhancer located ~2 Mb downstream of the *MYC* gene has been shown to physically interact with *MYC*, but the mechanisms responsible for this specific interaction are unclear (Shi et al., 2013). To gain insights into the potential role of DNA loop structures in gene control at the *MYC* locus, we generated cohesin HiChIP data for HCT-116 cells and collected published DNA interaction data for three other cancer cell types for comparison (Figure 1B; Tables S1 and S5) (Hnisz et al., 2016a; Pope et al., 2014). Among the DNA loop structures observed in these datasets, a large 2.8 Mb DNA loop was evident in all four cell types. This loop connects CTCF sites encompassing the *MYC* gene and qualifies as an insulated neighborhood. The DNA anchor sites of this 2.8 Mb DNA loop occur at the boundaries of a TAD found in all cells (Figure S1B). The *MYC* TAD encompasses a region previously described as a “gene desert,” because this large span of DNA contains no other annotated protein-coding genes (Montavon and Duboule, 2012; Ovcharenko et al., 2005).

While all cells examined appear to share the TAD-spanning 2.8 Mb loop encompassing *MYC*, the loop structures within the neighborhood were found to be markedly different among the tumor types. The internal loops were dominated by interactions between a *MYC* promoter-proximal CTCF site and the diverse super-enhancers (Figures 1B and 1C). The major differences between these internal structures in the different tumor cells involved the different positions of the tumor-specific super-enhancer elements. Examination of Hi-C data for a broader spectrum of tumor cell types suggests that tumor cells generally have DNA contacts between the *MYC* promoter-proximal site and other sites within the 2.8 Mb *MYC* TAD (Figure S1B). This looping was not limited to cancer cells, because examination of enhancer and promoter-capture Hi-C data in a variety of normal cell types that express *MYC* (Javierre et al., 2016) revealed that cell-type-specific enhancers do indeed loop to the *MYC* proximal CTCF site (Figures S1C and S1D). This indicated that this CTCF site is also used during normal development by cell-type-specific enhancers to facilitate *MYC* expression and cellular proliferation.

Further examination of the *MYC* promoter-proximal region revealed three constitutive CTCF binding sites (Figure 1C). All three sites were found to be occupied by CTCF in a wide variety of normal cells and tumor cells, and this binding pattern is shared across species (Figure S1C). Previous studies have examined the role of CTCF binding at all three sites (Filippova et al., 1996; Gombert and Krumm, 2009; Gombert et al., 2003; Klenova et al., 1993; Rubio et al., 2008). The two sites located within the *MYC* gene have been shown to play roles in *MYC* transcript start site selection and in promoter-proximal pausing of RNA polymerase II (Filippova et al., 1996). The CTCF binding site located 2 kb upstream of the major transcription start site has been reported to protect the promoter from methylation and to be an insulator element (Gombert and Krumm, 2009; Gombert et al., 2003). The DNA interaction data described here, however, suggests that this upstream site dominates connections with distal enhancer elements, as the majority of reads in the DNA interaction data are associated with this site in all tumor cells examined (Figures 1C and S1E). The −2 kb CTCF binding site contains a number of putative CTCF binding motifs; one of these most closely matches the canonical CTCF motif in the JASPAR database (Sandelin et al., 2004) and occurs within a highly conserved sequence (Figure 1D). These features, the presence of CTCF sites in tumor super-enhancers, and the ability of two CTCF-bound sites to be brought together through CTCF homodimerization (Saldaña-Meyer et al., 2014; Yusufzai et al., 2004) led us to further study the possibility that the −2 kb site has an enhancer-docking function critical to *MYC* expression.

### *MYC* Promoter Proximal CTCF Site Is Necessary for Enhancer-Promoter Looping and High *MYC* Expression

**Figure 2:**
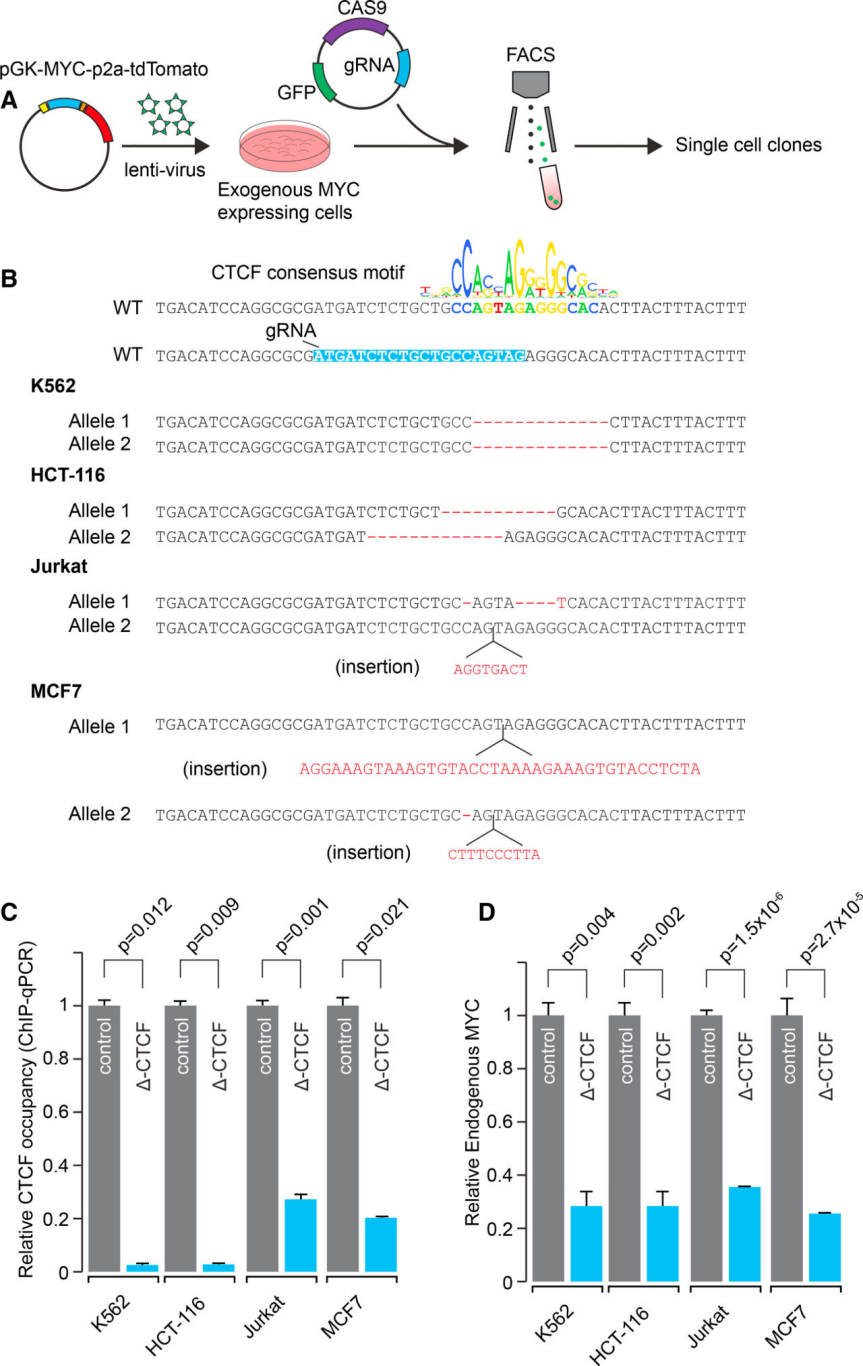
Perturbation of the Core CTCF Motif in the *MYC* CTCF Loop-Anchor Reduces CTCF Occupancy and *MYC* Expression (A) Schematic representation of the experiment. HCT-116, K562, Jurkat, and MCF7 cells were transduced with a construct expressing *MYC* under a *PGK* promoter and selected for successful integration. These cells were then transiently transfected with plasmid carrying Cas9 and a gRNA targeting the CTCF binding motif. Positive cells were identified and selected using fluorescence-activated cell sorting (FACS). These cells were multiplied, and clonal populations were characterized. (B) The DNA sequences in the vicinity of the core CTCF motif and the mutations generated in clonal populations of K562, HCT-116, Jurkat, and MCF7 cell lines. The reference (WT, wild-type) sequence highlighted in blue is complementary to the gRNA sequence targeting the most prominent CTCF motif (shown here in bold colored sequence). For the aneuploid MCF7 cell line, the two most common mutations are depicted. (C) ChIP-qPCR showing reduction of the CTCF occupancy in Δ-CTCF K562, HCT-116, Jurkat, and MCF7 cells. p values were generated with a Student’s t test. Error bars represent the SD of the mean from three technical replicates. (D) qPCR showing reduction of endogenous *MYC* mRNA levels in Δ-CTCF K562, HCT-116, Jurkat, and MCF7 cells. p values were generated with a Student’s t test. Error bars represent the SD of the mean from three biological replicates. See also Figure S2

To determine whether the putative enhancer-docking site plays a functional role in *MYC* expression through DNA loop formation, small perturbations of the CTCF binding site were generated in both alleles of the tumor cell lines K562, HCT-116, Jurkat, and MCF7 using clustered regularly interspaced short palindromic repeats (CRISPR)/Cas9 (Figures 2A and 2B). Attempts at genetic perturbation by transfection with constructs carrying CRISPR/Cas9 with a guide RNA specifically targeting the CTCF motif upstream of the *MYC* gene did not yield viable clones. To allow cells to continue to proliferate if the CTCF motif deletion was lethal, cells were virally transduced with an exogenous *MYC* gene driven by a phosphoglycerate kinase (*PGK*) promoter (Figure S2A). This construct contained sequence differences in the 3′ UTR that allowed discrimination between the endogenous and exogenous *MYC* mRNAs. Cells expressing this exogenous *MYC* gene were then subjected to CRISPR/Cas9 perturbation. Clones were selected with small deletions or insertions disrupting the canonical CTCF motif (Figures 2B and S2B) and these cells were further characterized. CTCF chromatin immunoprecipitation quantative polymerase chain reaction (ChIP-qPCR) showed complete loss of CTCF binding to this site in K562 and HCT-116 cells and a 60%–70% reduction in CTCF binding at this site in Jurkat and MCF7 cells (Figure 2C). RNA analysis revealed a 70%–80% reduction of endogenous *MYC* mRNA in the absence of the enhancer-docking site in all of these cell types (Figure 2D). Furthermore, an inducible CRISPR/Cas9 perturbation model showed reduced proliferation for these four cell types upon induction of CTCF-site deletions (Figures S2C–S2G). These results indicate that the CTCF motif in the *MYC* enhancer-docking site is necessary for CTCF binding, for high levels of *MYC* expression and for cellular proliferation.

**Figure 3:**
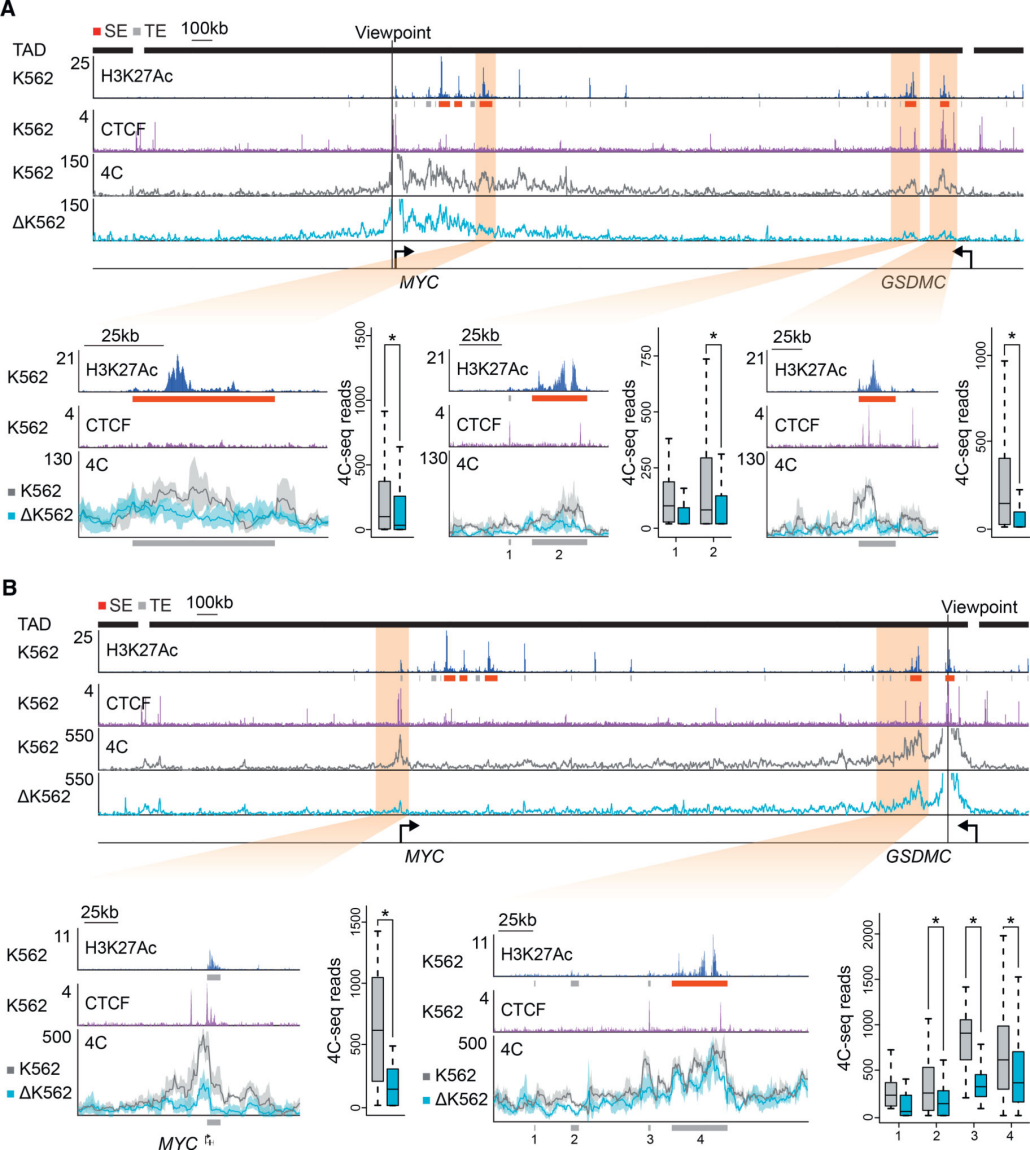
Perturbation of the *MYC* Enhancer-Docking Site Reduces Looping to Super-Enhancers (A) 4C analysis showing reduced looping of the *MYC* promoter proximal CTCF site to super-enhancers in CTCF motif deletion cells (ΔK562) versus unmodified cells (K562). H3K27Ac ChIP-seq and CTCF ChIP-seq are shown in blue and purple colors respectively. Blowups show the 4C interactions for three K562 specific super-enhancers. The 4C viewpoint is situated 112 base pairs upstream of the deleted loop-anchor region. (B) 4C analysis showing reduced looping of the *MYC* promoter proximal CTCF site to super-enhancers in CTCF motif deletion cells (ΔK562) versus unmodified cells (K562) using a viewpoint centered on the most distant super-enhancer downstream of the *MYC* gene. H3K27Ac ChIP-seq and CTCF ChIP-seq are shown in blue and purple, respectively. Blowups show the 4C interactions at the *MYC* promoter and distant super-enhancer near the viewpoint. Shading represents the 90% confidence interval based on three biological replicates. Peak calls from the H3K27Ac ChIP-seq were used to define the regions to be quantified and are indicated in gray boxes at the bottom of the panels. Boxplots show quantification of the reads per fragment for the indicated regions. p values were generated using Student’s t test, and data pairs with a p value < 0.05 are indicated with an asterisk. Reads are shown in reads per million sequenced reads per base pair. Typical enhancers and super-enhancers are shown as gray boxes and red boxes, respectively. See also Figure S3

If the putative *MYC* enhancer-docking site contributes to looping interactions with distal enhancers, then the loss of this site should cause a decrease in DNA interactions between the *MYC* promoter and the distal super-enhancers. We used chromosome conformation capture combined with high-throughput sequencing (4C-seq) to compare super-enhancer interactions in K562 and HCT-116 cells with normal or perturbed CTCF binding motifs. The 4C-seq data in K562 cells indicated that the *MYC* enhancer-docking site interacts predominantly with distal super-enhancers located ~0.3 Mb and ~2 Mb downstream of the *MYC* gene and that the majority of these interactions were significantly reduced when the putative CTCF motif was perturbed (Figures 3A and S3A). In order to control for any direct effects of a genetic alteration near the viewpoint, 4C-seq was performed with a viewpoint placed in the downstream super-enhancer. This showed clear interactions with the *MYC* enhancer-docking site as well as with the nearby super-enhancer, and these interactions were significantly reduced upon perturbation of the CTCF motif (Figures 3B and S3B). Similar results were obtained in HCT-116 cells, where the viewpoint was centered on the super-enhancer located ~0.4 Mb upstream of the *MYC* gene (Figure S3C). These results showed that the CTCF site in the promoter-proximal region of *MYC* is important for optimal interaction with distal enhancers and supports the idea that this CTCF site functions as an enhancer-docking site.

### Loss of *MYC* Expression with Methylation of Enhancer-Docking Site

**Figure 4:**
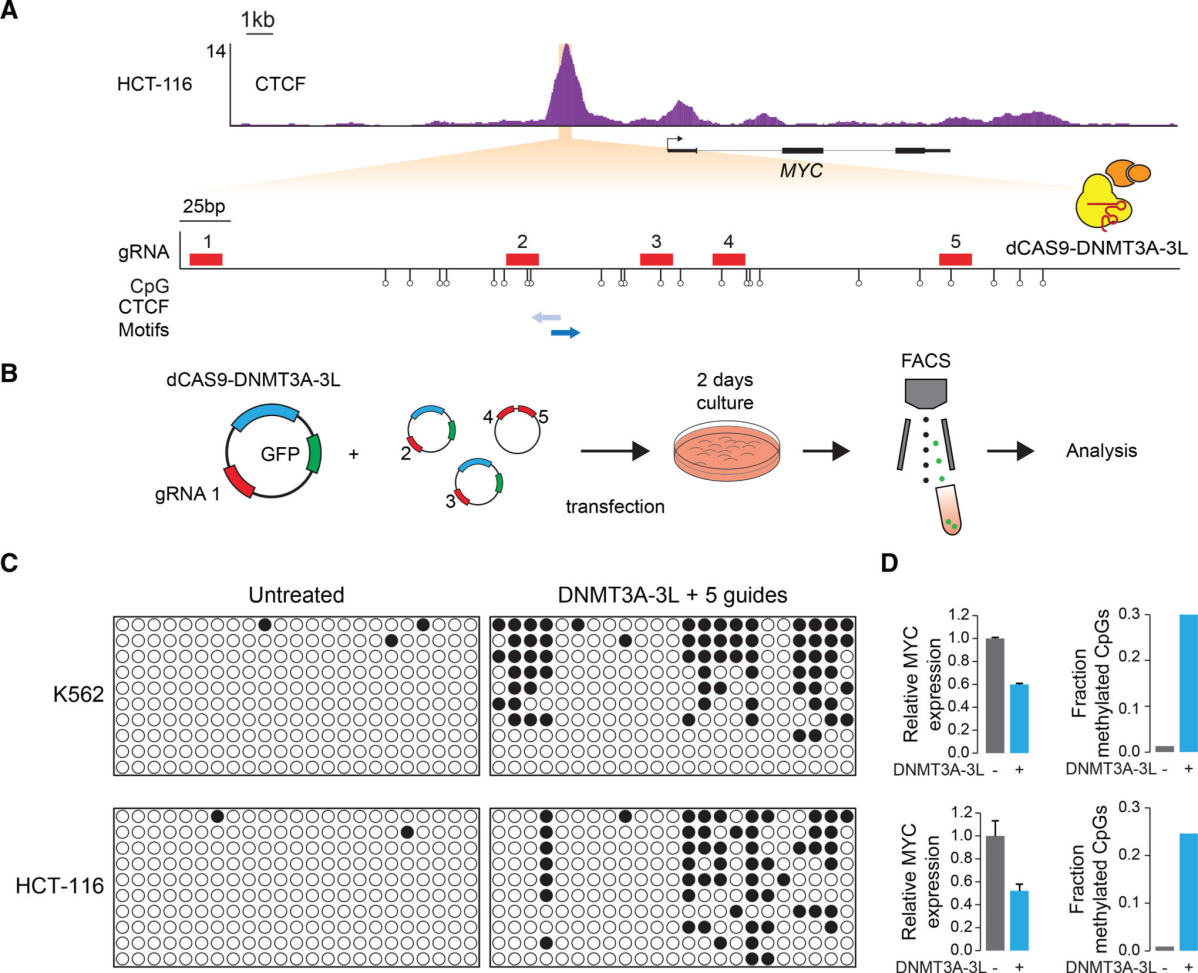
dCas9-Mediated Methylation of the CTCF Loop-Anchor Site Reduces *MYC* Expression in Tumor Cells (A) Top panel shows CTCF ChIP-seq at the *MYC* gene region in HCT-116 cells. ChIP-seq reads are shown in reads per million sequenced reads per base pair. Bottom panels shows a blowup of the ~700-bp region underneath the CTCF peak depicting the CTCF motifs (blue arrows) and the gRNAs (red rectangles) used to target dCas9-DNMT3A-3L to the enhancer anchor. Lollipop symbols indicate the location of CpGs that are assayed for methylation levels in (C). (B) Schematic representation of the experiment. HCT-116 or K562 cells were transfected with plasmids encoding the dCAS9-DNMT3A-3L, GFP, and a gRNA together with a plasmid encoding 2 additional gRNAs. HCT-116 or K562 cells were isolated by FACS after 2 days, and DNA and RNA were isolated. (C) Methylation at *MYC* promoter loop-anchor site in untreated cells and cells transfected with dCas9-DNMT3A-3L in conjunction with the 5 indicated gRNAs. (D) qPCR analysis of *MYC* mRNA levels and fraction of methylated CpGs for untreated and dCas9-DNMT3A-3L + 5 gRNA transfected cells. Error bars represent the SD of the mean for three biological replicates. See also Figure S4

CTCF binding is abrogated when its sequence motif is methylated (Bell and Felsenfeld, 2000; Maurano et al., 2015), and the *MYC* enhancer-docking site occurs within a CpG island that is consistently hypomethylated in different tumor types as well as in different normal tissues (Figures S4A and S4B). The recent development of tools that permit site-specific DNA methylation (Liu et al., 2016; Siddique et al., 2013) suggested a means to disrupt *MYC* expression by methylation of the enhancer-docking site. To achieve targeted methylation, we created a construct to express a dCas9 fusion protein consisting of the catalytic domain of DNMT3A and the interacting domain of DMNT3L. This dCas9-DNMT3A-3L protein was targeted to the *MYC* enhancer-docking site in K562 and HCT-116 cells using multiple guide RNAs that span the region (Figures 4A and 4B). The targeting of dCas9-DNMT3A-3L resulted in robust local DNA methylation (Figure 4C) and a 40%–50% reduction in mRNA levels in both cell types (Figure 4D). The methylated region likely contains binding sites for additional TFs that may be sensitive to DNA methylation, so it is possible that the reduced mRNA levels are due to multiple factors. In order to test whether disruption of TFs other than CTCF contribute to the reduction in *MYC* mRNA levels, the dCas9-DNMT3A-3L was targeted to the *MYC* enhancer-docking site in CTCF-site deleted K562 cells. No further reduction of *MYC* mRNA levels was observed under these conditions (Figures S4C and S4D), indicating that loss of CTCF was a major contributor to the observed reduction of *MYC* expression upon targeted methylation of the *MYC* enhancer-docking site. These results demonstrate that epigenetic editing of the enhancer-docking site can reduce *MYC* expression.

### CTCF Enhancer-Docking Sites at Additional Genes

**Figure 5:**
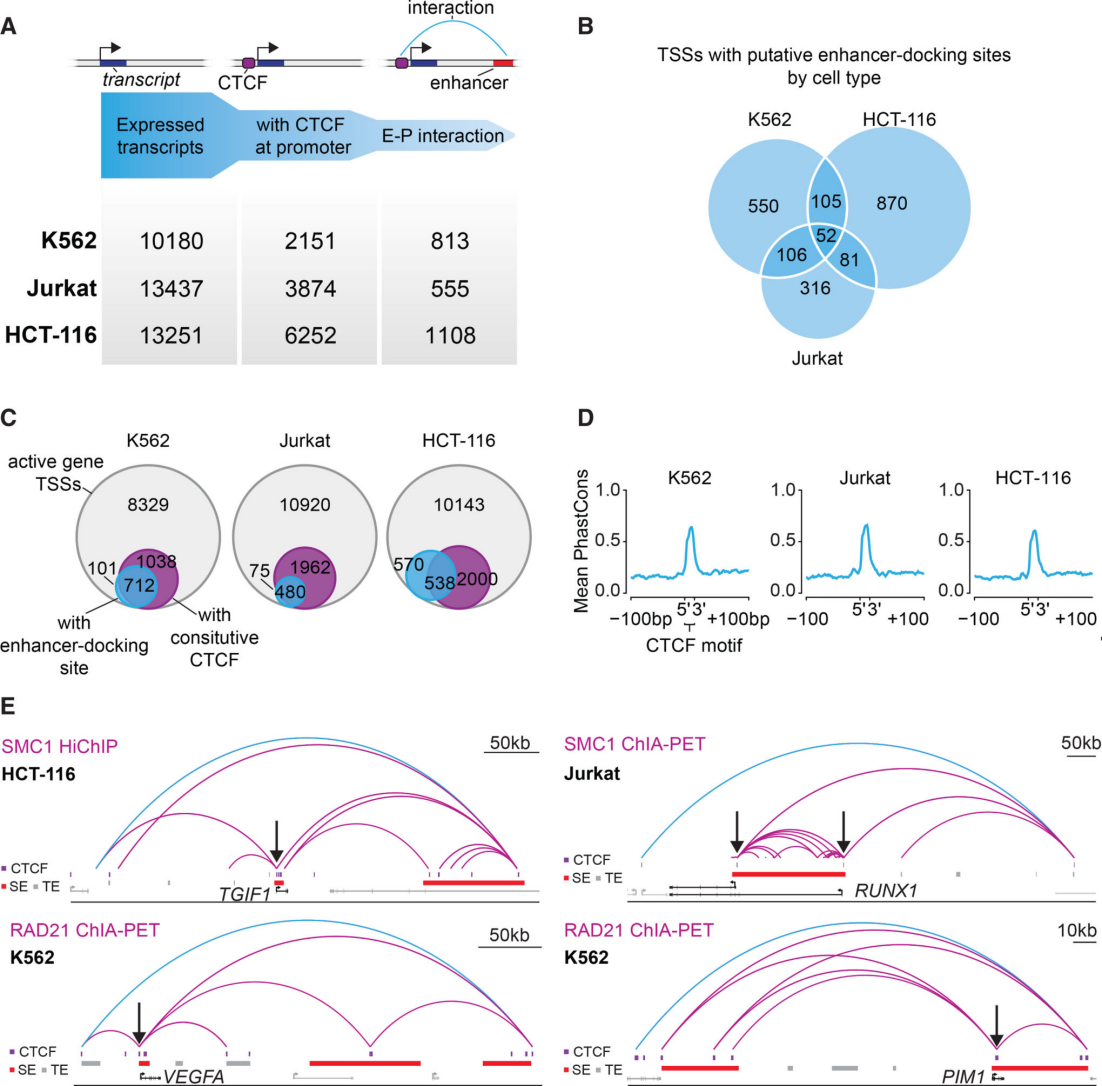
Putative Enhancer-Docking Sites Occur at Additional Genes with Prominent Roles in Cancer (A) Identification of genes with putative enhancer-docking sites. Genes were filtered for their expression status, presence of a CTCF binding site within 2.5 kb of the TSS and evidence of looping to an active enhancer, defined by H3K27Ac ChIP-seq. (B) Venn-diagram showing the overlap of TSSs with putative CTCF enhancer docking in K562, HCT-116, and Jurkat cells. (C) Venn-diagrams showing the number of TSSs from active genes, the number of these that exhibit putative CTCF enhancer-docking and how many of these have a constitutive CTCF site within 2.5 kb of the TSS. (D) Conservation analysis of the CTCF motifs in the CTCF-bound elements in putative enhancer-docking sites. The mean 46-way PhastCons score of the highest JASPAR scoring motifs in CTCF peaks within putative CTCF enhancer docking and their flanking regions are shown. (E) Examples of genes with putative CTCF enhancer-docking sites from the different cell types analyzed. CTCF ChIP-seq peaks are shown as purple rectangles, typical enhancers are shown as gray rectangles, and super-enhancers are shown as red rectangles. Black arrows indicate the CTCF sites that may facilitate enhancer docking. The insulated neighborhood loop is shown in blue and loops internal to it are shown in purple. HCT-116 HiChIP interactions internal to the neighborhood with an origami score of at least 0.9 and a minimum PET count of 15 are shown for the *TGIF1* locus. Jurkat SMC1 ChIA-PET interactions internal to the neighborhood with an origami score of at least 0.97 are shown for the *RUNX1* locus. K562 RAD21 ChIA-PET interactions internal to the neighborhood with an origami score of at least 0.9 and a minimum PET count of 30 are shown for the *VEGFA* locus. K562 RAD21 ChIA-PET interactions internal to the neighborhood with an origami score of at least 0.9 and aminimum PET count of 30 are shown for the *PIM1* locus. Data are from this study and two others (Hnisz et al., 2016a; Heidari et al., 2014). See also Figure S5

Previous genomic studies have noted that CTCF might engender enhancer-promoter interactions at a minority of genes (Banani et al., 2017; Nora et al., 2017; Splinter et al., 2006; Zuin et al., 2014). We therefore identified the set of genes whose promoter-proximal regions contain CTCF-bound sites and that show evidence of enhancer interactions in K562, Jurkat, and HCT-116 cells. We identified all active transcription start sites (TSSs) that have at least one CTCF-bound site within 2.5 kb of the TSS that interacts with at least one enhancer. This yielded between 555 and 1,108 TSSs with a nearby CTCF site that loops to an active enhancer (Figure 5A; Table S2). We define these TSSs as having a putative CTCF enhancer-docking site. The majority of TSSs identified in this analysis were identified in only one cell type, with only 52 TSSs identified in all three cell types (Figure 5B). Nonetheless, these putative enhancer-docking sites tended to be constitutively bound by CTCF in all three cell types, and the CTCF motifs in these sites showed high sequence conservation (Figures 5C and 5D). This suggests that these putative enhancer-docking sites are occupied by CTCF regardless of interaction with active enhancers and that differences in cell-type-specific enhancers are largely responsible for differential use of enhancer-docking site genes in these cells.

Gene ontology analysis of the genes with putative enhancer-docking sites found different processes to be significantly enriched in each cell type, and these processes were dominated by the cellular identity of the cell lines (Figure S5A; Table S3). Common processes among the three cell types include cell cycle and other cancer-related processes such as gene expression and response to signaling (Figure S5A). A number of cancer-associated genes were found, including *TGIF1*, *VEGFA*, *RUNX1*, and *PIM1* (Figure 5E), as well as others (Figure S5B). We conclude that genes other than *MYC* are likely regulated by CTCF-bound enhancer-docking sites and that these include multiple cancer-associated genes.

## DISCUSSION

Aberrant transcriptional activation of the *MYC* oncogene occurs frequently in tumor cells and is associated with tumor aggression. *MYC* resides within a 2.8 Mb TAD and its aberrant activation is generally accomplished by acquisition of a super-enhancer somewhere within that TAD. How these diverse cancer-specific super-enhancers loop long distances to specifically interact with *MYC* has not been clear. We find that the diverse super-enhancers commonly interact with, and depend on, a conserved CTCF binding site located 2 kb upstream of the *MYC* promoter. Because tumor super-enhancers can encompass genomic regions as large as 200 kb, and CTCF occupies sites that occur on average every 10 kb, there is considerable opportunity for super-enhancers to adventitiously contain a CTCF-bound site, which in turn could serve to interact with the *MYC* CTCF site (Table S6). Thus, different tumor super-enhancers have the opportunity to form through diverse mechanisms throughout this large TAD and can exploit the *MYC* CTCF site to interact with and activate *MYC* expression.

The concept that enhancer-promoter interactions generally occur within larger chromosomal loop structures such as TADs, which are themselves often formed by the interaction of CTCF proteins bound to each of the TAD loop anchors (Dekker and Mirny, 2016; Fraser et al., 2015; Gorkin et al., 2014a; Hnisz et al., 2016a), is supported by the observations described here. These larger loop structures tend to insulate enhancers and genes within the CTCF-CTCF loops from elements outside those loops. Constraining DNA interactions within CTCF-CTCF loop structures in this manner may facilitate proper enhancer-promoter contacts.

The evidence described here argues that diverse human tumor cell super-enhancers depend on the *MYC* CTCF site for optimal levels of enhancer-promoter looping and mRNA expression. A recent independent study in K562 cells used a tiling CRISPR screen to systematically perturb the *MYC* locus and also found that full *MYC* expression and cell proliferation is dependent on this region (Fulco et al., 2016). However, deletion of the −2 kb CTCF site has limited effects on *MYC* expression in mice (Dave et al., 2017; Gombert and Krumm, 2009), and some translocated enhancers can drive *MYC* expression in the absence of this CTCF site (Shiramizu et al., 1991). There are several potential explanations for these diverse results. It is possible that the −2 kb CTCF site is important for optimal *MYC* expression levels in human cells, but not in mice. It is conceivable that the deletion of a region containing the CTCF site can be compensated by features of the new enhancer landscape in the deletion mutations. Furthermore, additional mechanisms normally involved in enhancer-promoter interactions, such as YY1-YY1 interactions, may mask the loss of the CTCF site *in vivo*; YY1 is present in the *MYC* promoter region and is thus likely to contribute to DNA looping and expression (Weintraub et al., 2017).

Our studies suggest that an additional set of human genes, beyond *MYC*, may utilize promoter-proximal enhancer-docking sites to mediate cell-type-specific enhancer-promoter interactions. Such CTCF-mediated enhancer-promoter interactions are generally nested within larger CTCF-mediated loops that would function as insulated neighborhoods. At these genes with CTCF-mediated enhancer docking, the promoter-proximal enhancer-docking sites tend to be constitutively bound by CTCF and these binding sites tend to be highly conserved. Indeed, two studies have reported that these genes tend to lose expression upon perturbation of CTCF (Nora et al., 2017; Zuin et al., 2014), consistent with a role for CTCF in enhancer-promoter looping. Among these genes are cancer-associated genes that likely employ this mechanism to engender interactions with tumor-specific enhancers. For example, at *CSNK1A1*, a drug target in acute myeloid leukemia (AML) tumor cells (Järås et al., 2014), *VEGFA*, which is upregulated in many cancers (Goel and Mercurio, 2013), and *RUNX1*, a well-defined oncogene in AML (Deltcheva and Nimmo, 2017), the evidence suggests that super-enhancers in these cancer cells use a CTCF enhancer-docking mechanism to interact with the oncogene. Thus, a CTCF-dependent enhancer-docking mechanism, which presumably facilitates interaction with different cell-specific enhancers during development, is exploited by cancer cells to dysregulate expression of prominent oncogenes.

*MYC* dysregulation is a hallmark of cancer (Bradner et al., 2017). The c-MYC TF is an attractive target for cancer therapy because of the role that excessive c-MYC levels play in a broad spectrum of aggressive cancers (Felsher and Bishop, 1999; Jain et al., 2002; Soucek et al., 2008), but direct pharmacologic inhibition of c-MYC remains an elusive challenge in drug discovery (Bradner et al., 2017). The *MYC* enhancer-docking site, and presumably those of other oncogenes, can be repressed by dCas9-DNMT-mediated DNA methylation. Oncogene enhancer-docking sites may thus represent a vulnerability in multiple human cancers.

## EXPERIMENTAL PROCEDURES

Further details and an outline for resources used in this work can be found in Supplemental Experimental Procedures.

### CRISPR/Cas9 Genome Editing

Genome editing was performed using CRISPR/Cas9 essentially as described previously (Ran et al., 2013). The genomic sequences complementary to all guide RNAs are listed in Table S4.

### ChIP-Seq

ChIP was performed as described previously (Lee et al., 2006). Approximately 30 million cells were crosslinked for 10 min at room temperature by the addition of one-tenth of the volume of 11% formaldehyde solution to the growth media followed by 5 min quenching with 125 mM glycine. Cells were washed twice with PBS, and then the supernatant was aspirated and the cell pellet was flash frozen at −80°C. 100 µL Protein G Dynabeads (Thermo 10003D) were blocked with 0.5%BSA (w/v) in PBS. Magnetic beads were bound with 40 µL anti-CTCF antibody (Millipore 07–729). Nuclei were isolated as previously described (Lee et al., 2006) and sonicated in lysis buffer on a Misonix 3000 sonicator for 5 cycles at 30 s each on ice (18–21 W) with 60 s on ice between cycles. Sonicated lysates were cleared once by centrifugation and incubated overnight at 4°C with magnetic beads bound with antibody to enrich for DNA fragments bound by the indicated factor. Beads were washed with wash buffers A, B, C, and D sequentially. DNA was eluted, cross-links were reversed, and DNA was purified with phenol chloroform extraction and ethanol precipitation. Libraries for Illumina sequencing were prepared following the Illumina TruSeq DNA Sample Preparation v2 kit and sequenced on the Illumina HiSeq 2500 for 40 bases in single-read mode.

### 4C-Seq

A modified version of 4C-seq (van de Werken et al., 2012) was developed (Supplemental Experimental Procedures). The major change was the ligation is performed in intact nuclei (*in situ*). This change was incorporated because previous work has noted that *in situ* ligation dramatically decreases the rate of chimeric ligations and background interactions (Rao et al., 2014).

### HiChIP

HiChIP was performed essentially as described (Mumbach et al., 2016). 10 million HCT116 cells were crosslinked for 10 min at room temperature by the addition of one-tenth of the volume of 11% formaldehyde solution to the growth media followed by 5-min quenching with glycine. Cells were washed twice with PBS, and then the cell pellet was flash frozen in liquid nitrogen. Frozen samples were processed according to protocol (Supplemental Experimental Procedures).

### Targeted Methylation and Bisulfite Sequencing

To perform targeted methylation, cells were transfected with a dCas9-DNMT3A-3L construct and five guides. To generate the dCas9-DNMT3A-3L construct, dCas9 was isolated from pSQL1658 (Addgene 51023) by PCR. Cas9 was removed from pX330-Cas9 (Addgene 42230) and replaced by DNMT3A-3L (Siddique et al., 2013). Guide RNAs used for targeting can be found in Table S4.

### Statistical Methods

#### ChIP-Seq Data Analysis

ChIP-seq datasets were generated for this study as well as collated from previous studies (Table S5) and were aligned using Bowtie (version 0.12.2) to the human genome (build hg19, GRCh37) with parameter -k 1 -m 1 -n 2. We used MACS version 1.4.2 with the parameter “-no-model-keep-dup=auto.” A p value threshold of enrichment of 1e-09 was used.

#### 4C Analysis

4C-seq reads were trimmed and mapped using bowtie with options -k 1 -m 1 against the hg19 genome assembly. We only used the reads from non-blind fragments for further analysis. The normalized profile of each sample was smoothened using a 6-kb running mean at 500-bp steps across the genome. Quantification of the 4C signal counted the reads per fragment per million sequenced reads in the super-enhancers or the CTCF MACS peak calls.

#### HiChIP and ChIA-PET Data Analysis

We developed a new software pipeline and analytical method called origami to process HiChIP and chromatin interaction analysis by paired-end tag sequencing (ChIA-PET). The software and releases can be found at https://github.com/younglab/origami using version alpha20160828. The ChIA-PET datasets analyzed along with their corresponding linker sequence and called interactions in and around the *MYC* TAD can be found in Table S4. Each ChIA-PET datasets was processed as follows: the reads were first trimmed and aligned using origami-alignment. Each end of a paired end tag (PET) with a linker sequence were separately mapped to the hg19 genome assembly using bowtie with the following options: -v 1 -k 1 -m 1. After alignment, the separated PETs were re-paired in the final BAM output. After repairing, all duplicated PETs within the data were removed. Peaks were called on the re-paired ChIA-PET reads using MACS1 v1.4.2 with the following parameters: -nolambda -nomodel -p 1e–9.

## Supplementary Material

1

2

3

4

5

6

7

8

## Highlights

- Tumor-specific super-enhancers loop to an enhancer-docking site at the *MYC* oncogene
- Deletion or methylation of *MYC* enhancer-docking site reduces enhancer-promoter looping
- *MYC* enhancer-docking site provides common vulnerability in diverse cancer cells
- Similar docking sites for multiple enhancers occur at additional genes
