# Supplementary material for: Transcriptional Dysregulation of MYC Reveals Common Enhancer-Docking Mechanism
Source: Cell Rep. Author manuscript; Available in PMC 2018 May 1. (PMC5929158; doi:10.1016/j.celrep.2018.03.056)
Supplement: 1 [file NIHMS961951-supplement-1.pdf]

Cell Reports, Volume *23*

## Supplemental Information

# Transcriptional Dysregulation of *MYC* Reveals Common Enhancer-Docking Mechanism

**Jurian Schuijers, John Colonnese Manteiga, Abraham Selby Weintraub, Daniel Sindt Day, Alicia Viridiana Zamudio, Denes Hnisz, Tong Ihn Lee, and Richard Allen Young**

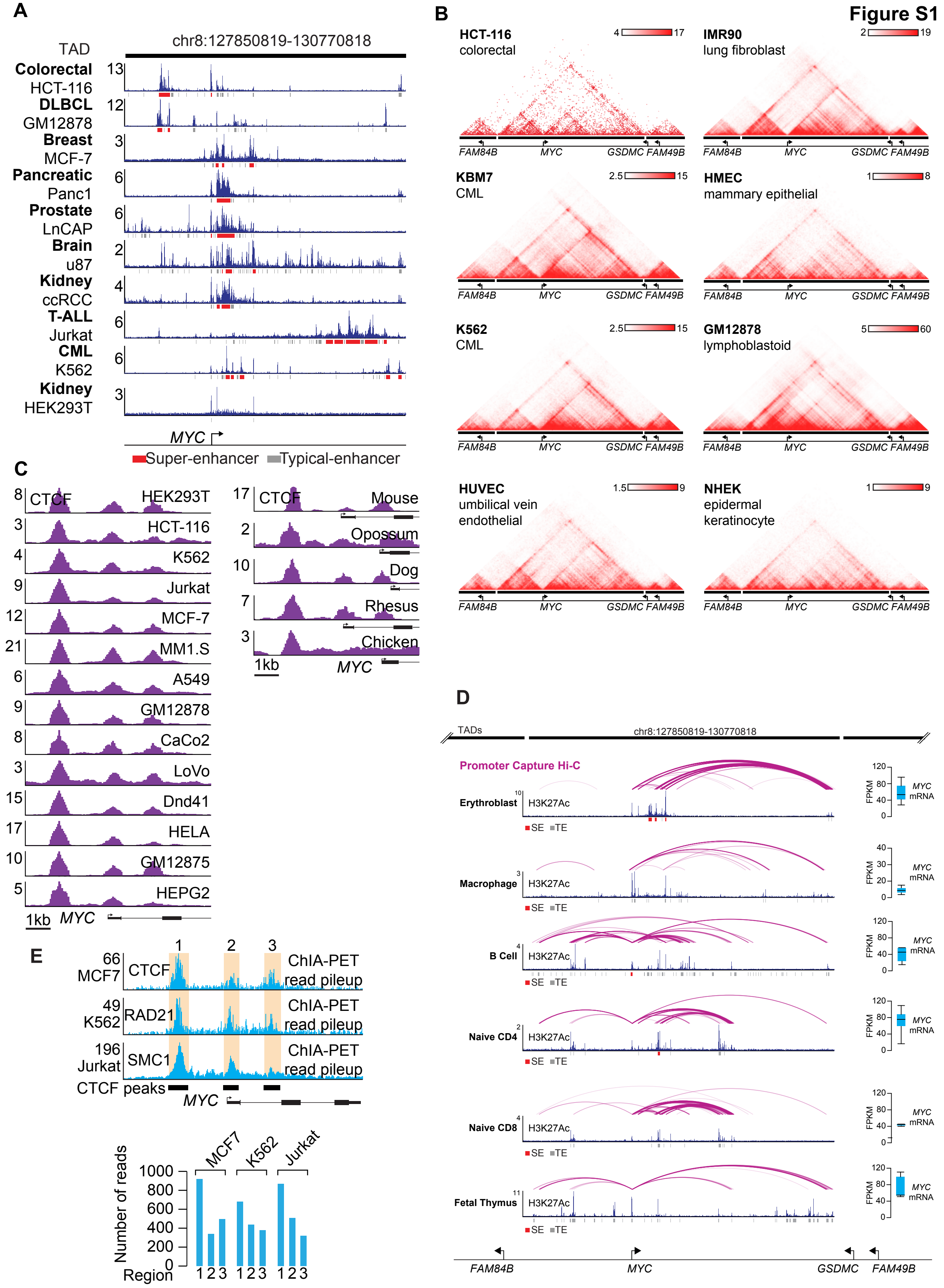
Figure S1
A
TAD
chr8:127850819-130770818
Colorectal HCT-116
DLBCL GM12878
Breast MCF-7
Pancreatic Panc1
Prostate LnCAP
Brain u87
Kidney ccRCC
T-ALL Jurkat
CML K562
Kidney HEK293T
MYC
Super-enhancer
Typical-enhancer
B
HCT-116 colorectal
IMR90 lung fibroblast
KBM7 CML
HMEC mammary epithelial
K562 CML
GM12878 lymphoblastoid
HUVEC umbilical vein endothelial
NHEK epidermal keratinocyte
FAM84B MYC GSDMC FAM49B
C
CTCF
HEK293T
HCT-116
K562
Jurkat
MCF-7
MM1.S
A549
GM12878
CaCo2
LoVo
Dnd41
HELA
GM12875
HEPG2
Mouse
Opossum
Dog
Rhesus
Chicken
1kb
MYC
D
TADs
chr8:127850819-130770818
Promoter Capture Hi-C
Erythroblast
Macrophage
B Cell
Naive CD4
Naive CD8
Fetal Thymus
H3K27Ac
SE TE
FPKM
MYC mRNA
FAM84B MYC GSDMC FAM49B
E
MCF7 CTCF ChIA-PET read pileup
K562 RAD21 ChIA-PET read pileup
Jurkat SMC1 ChIA-PET read pileup
CTCF peaks
MYC
Number of reads
Region
MCF7 K562 Jurkat

**Figure S1. Cell type-specific super-enhancers in the *MYC* locus loop to a common CTCF site. Related to Figure 1.**

(**A**) The 2.8 Mb TAD containing the *MYC* gene is indicated with thick black horizontal lines. H3K27Ac ChIP-seq signal (reads per million sequenced reads per base pair, data from this manuscript, (Becket et al., 2016; Frietze et al., 2012; Lin et al., 2012; Pope et al., 2014; Wang et al., 2011)) is shown in dark blue for a panel of tumor cell lines that express *MYC*. Tumor super-enhancers in the *MYC* TAD are depicted as red rectangles and typical enhancers are depicted as grey rectangles. (**B**) Heatmap of the ORIGAMI processed HiChIP, unfiltered data showing the *MYC* TAD with flanking regions (chr8:127100000-131525000) and Heatmaps of Hi-C interaction data showing the *MYC* TAD with flanking regions (chr8:127100000-131525000) across seven different cell types (data from (Rao et al., 2014)). The HiChIP was not smoothened as opposed to the smoothened HiC data. Scale bars represent the contrast settings used, numbers indicate the maximum intensity cutoff. The color intensity represents the PET count and the cutoff is represented in PET numbers for the HiChIP data. (**C**) CTCF ChIP-seq across a panel of tumor cell lines (data from (Anders et al., 2014; Encode Consortium, 2012; Hnisz et al., 2016; Pope et al., 2014; Wang et al., 2012; Yan et al., 2013)), and from mouse T-helper cells, Opossum, Dog, and Rhesus macaque liver (data from (Schmidt et al., 2012; Stadler et al., 2011)). Read counts are shown in reads per million sequenced reads per base pair.

(**D**) Promoter Hi-C interaction data and H3K27Ac ChIP-seq at the *MYC* TAD for cell types that represent different stages in hematopoietic development. The 2.8 Mb TAD containing *MYC* and part of the two adjacent TADs are indicated with thick black horizontal lines. Promoter Hi-C interactions are shown as purple colored arcs; the intensity of purple color reflects the confidence score from (Javierre et al., 2016). H3K27Ac ChIP-seq signal is shown, measured in reads per million sequenced reads per base pair (data from(Bernstein et al., 2010; Encode Consortium, 2012; Schmidt et al., 2016; Xu et al., 2012)). Super-enhancers are depicted as red rectangles and typical enhancers as grey rectangles. The relative level of *MYC* transcripts in the corresponding cell types are shown as boxplots in fragments per kilobase of exon per million sequenced reads (FPKM), expression data from the BLUEPRINT consortium, fetal thymus expression data from the ENCODE consortium. (**E**) ChIA-PET read pileups at the *MYC* promoter and quantification of the reads in the three CTCF peaks indicated. Light blue tracks display the read counts from read pileups of MCF7 CTCF, K562 RAD21 and Jurkat SMC1 ChIA-PET data showing that the majority of the ChIA-PET reads are found at the enhancer-docking site. Reads are shown as read counts per base pair.

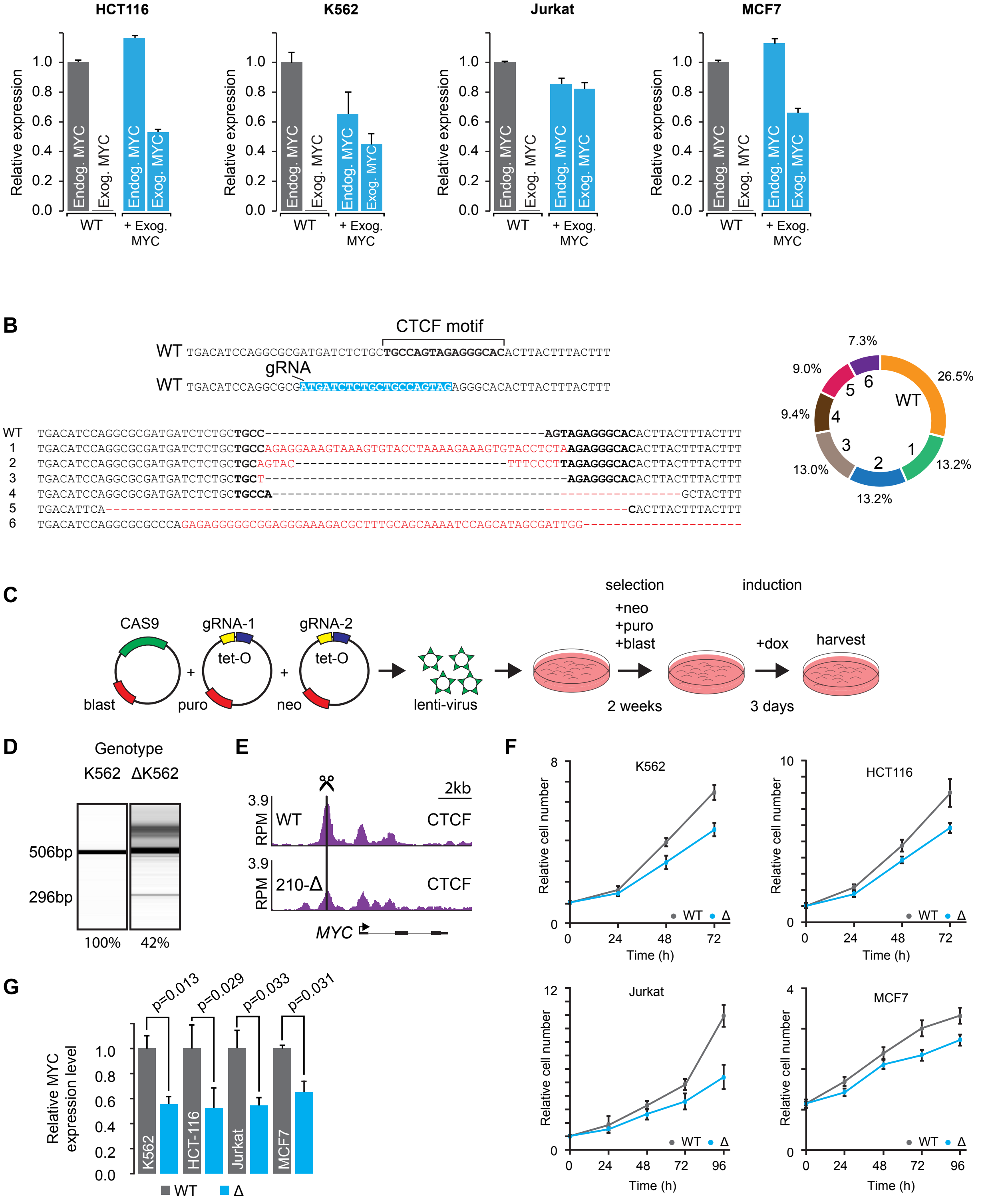
A
HCT116
K562
Jurkat
MCF7
Relative expression
Endog. MYC
Exog. MYC
WT
+ Exog. MYC
B
CTCF motif
WT TGACATCCAGGCGCGATGATCTCTGCTGCCAGTAGAGGGCACACTTACTTTACTTT
gRNA
WT TGACATCCAGGCGCGATGATCTCTGCTGCCAGTAGAGGGCACACTTACTTTACTTT
WT TGACATCCAGGCGCGATGATCTCTGCTGCC-----AGTAGAGGGCACACTTACTTTACTTT
1 TGACATCCAGGCGCGATGATCTCTGCTGCCAGAGGAAAGTAAAGTGTACCTAAAAGAAAGTGTACCTCTAAGAGGGCACACTTACTTTACTTT
2 TGACATCCAGGCGCGATGATCTCTGCTGCAGTAC-----TTTCCCTTAGAGGGCACACTTACTTTACTTT
3 TGACATCCAGGCGCGATGATCTCTGCTGCT-----AGAGGGCACACTTACTTTACTTT
4 TGACATCCAGGCGCGATGATCTCTGCTGCCA-----GCTACTTT
5 TGACATTCA-----CACTTACTTTACTTT
6 TGACATCCAGGCGCGCCCAGAGAGGGGGCGGAGGGAAAGACGCTTTGCAGCAAAATCCAGCATAGCGATTGG-----
7.3%
26.5%
9.0%
9.4%
13.0%
13.2%
13.2%
WT
C
CAS9
gRNA-1
gRNA-2
tet-O
blast
puro
neo
lenti-virus
selection
+neo
+puro
+blast
2 weeks
induction
+dox
3 days
harvest
D
Genotype
K562 ΔK562
506bp
296bp
100% 42%
E
2kb
3.9
RPM
WT
CTCF
210-Δ
MYC
F
K562
HCT116
Jurkat
MCF7
Relative cell number
Time (h)
WT Δ
G
p=0.013
p=0.029
p=0.033
p=0.031
Relative MYC expression level
K562
HCT-116
Jurkat
MCF7
WT Δ

**Figure S2. Perturbation of the core CTCF motif in the *MYC* CTCF loop-anchor reduces CTCF occupancy and *MYC* expression. Related to Figure 2.**

(**A**) qPCR measuring the mRNA levels of endogenous (endog.) and exogenous (exog.) *MYC* in parental (wild type) and exogenous *MYC* expressing HCT-116, K562, MCF7 and Jurkat cells. Endogenous and exogenous *MYC* were detected using primers directed against the 3' UTR of the *MYC* mRNA and the *MYC*-tdTomato junction respectively. (**B**) Sequencing of mutant alleles in the selected MCF7 clone with mutated enhancer-docking site. The CRISPR targeted region was amplified, fragmented and sequenced to identify the composition and frequency of mutant alleles. The 6 most common mutant alleles are displayed. (**C**) Perturbation of *MYC* promoter proximal CTCF site reduces *MYC* expression and proliferation rate across cancers. Schematic representation of the experiment. Cells were transduced with one virus carrying Cas9 and two viruses each carrying one guide RNA (gRNA) under a doxycycline inducible promoter. After selection for all three components, cells were induced with doxycycline for 3 days prior to harvest and testing. (**D**) Heat map of fragment lengths after genotyping PCR of wild type K562 and ΔK562 cells. PCR product was analyzed with a Fragment Analyzer and fragments of different lengths were quantified. The percentage of fragments with a length expected from wild type cells (506) is indicated under the lanes. The expected length of the PCR product for deleted and recombined alleles is 296bp. (**E**) ChIP-seq of CTCF in induced or uninduced K562 cells targeting the 210bp fragment containing the -2kb CTCF site. The -2kb CTCF binding is reduced, while the other CTCF binding sites in the *MYC* locus are unaffected. (**F**) Proliferation of parental (grey) and CTCF site deleted (blue) K562, HCT-116, Jurkat and MCF7 cells. Error bars represent the standard deviation of the mean from six biological replicates. (**G**) qPCR showing the *MYC* mRNA levels after deletion of the CTCF site in K562, HCT-116, Jurkat and MCF7 cells. p-values were generated with a Students T-test. Error bars represent the standard deviation of the mean from three biological replicates.

**Figure S3**

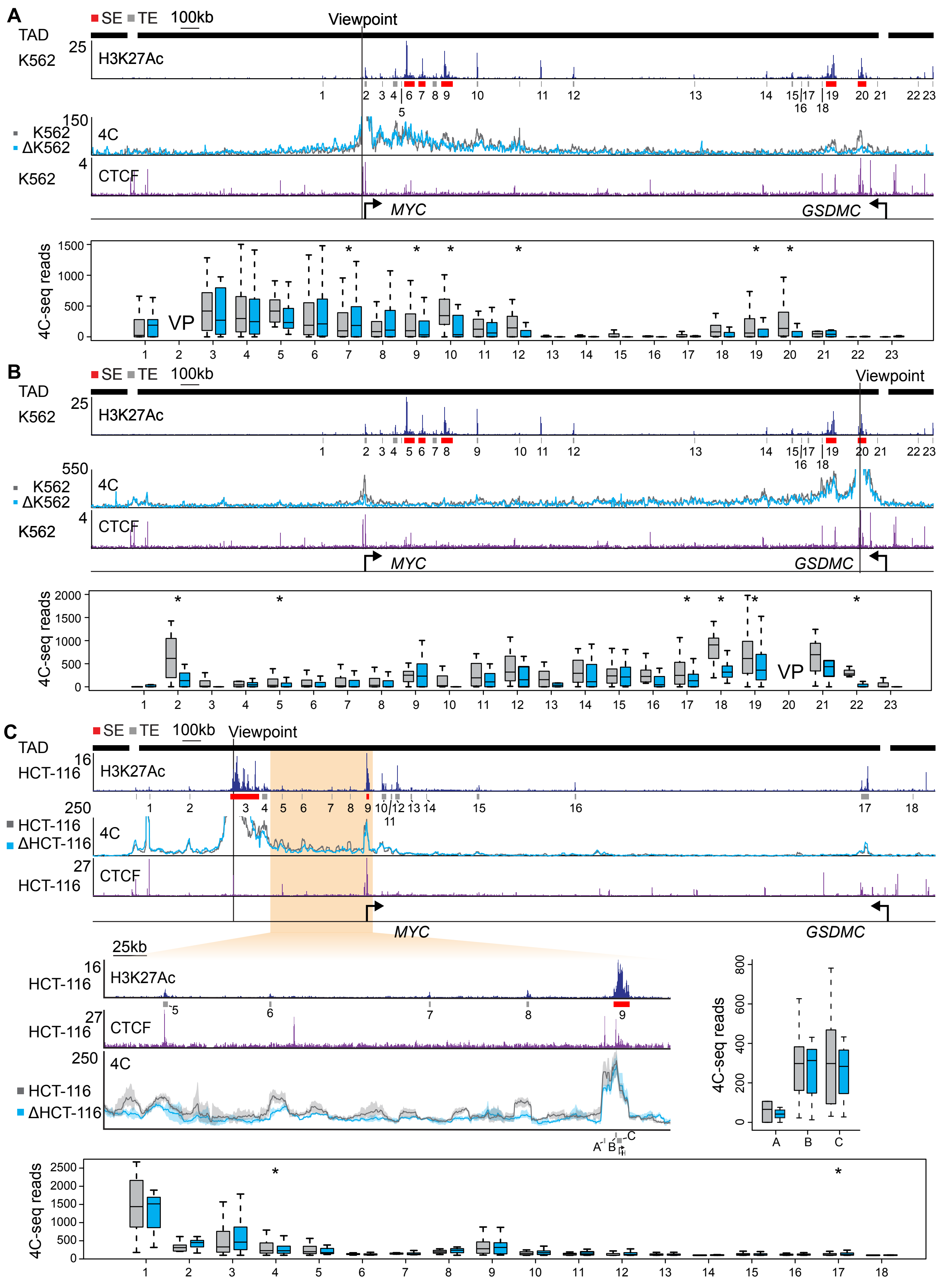

A
SE TE 100kb
Viewpoint
TAD
K562
25
H3K27Ac
1 2 3 4 5 6 7 8 9 10 11 12 13 14 15 16 17 18 19 20 21 22 23
K562
ΔK562
150
4C
4
K562
CTCF
MYC
GSDMC
4C-seq reads
VP
B
SE TE 100kb
Viewpoint
TAD
K562
25
H3K27Ac
550
4C
CTCF
MYC
GSDMC
4C-seq reads
VP
C
SE TE 100kb Viewpoint
TAD
16
HCT-116
H3K27Ac
250
HCT-116
ΔHCT-116
4C
27
HCT-116
CTCF
MYC
GSDMC
25kb
4C-seq reads
A B C

**Figure S3. Perturbation of the *MYC* enhancer-docking site reduces looping to super-enhancers. Related to figure 3.**

(**A**) 4C analysis showing quantification of regions based on H3K27Ac ChIP-seq peak calls for the 4C experiment with the viewpoint (VP) at the -2kb upstream CTCF site in K562 cells. (**B**) 4C analysis showing quantification of regions based on H3K27Ac ChIP-seq peak calls for the 4C experiment with the viewpoint (VP) at downstream super-enhancer in K562 cells. (**C**) 4C analysis showing quantification of regions based on H3K27Ac or CTCF ChIP-seq peak calls for the 4C experiment with the viewpoint (VP) at upstream super-enhancer in HCT-116 cells. Blowup shows the 4C interactions for the intervening region between the super-enhancer and the *MYC* gene. H3K27Ac ChIP-seq and CTCF ChIP-seq are shown in blue and purple, respectively. Shading represents the 90% confidence interval based on three biological replicates. Grey bars indicate the regions that are quantified. Box plots show quantification of the reads per fragment for the indicated regions. p-values were generated using Students T-test and data pairs with a p-value of $< 0.05$ are indicated with an asterisk. Reads are shown in reads per million sequenced reads per base pair. Typical-enhancers and super-enhancers are shown as grey boxes and red boxes respectively.

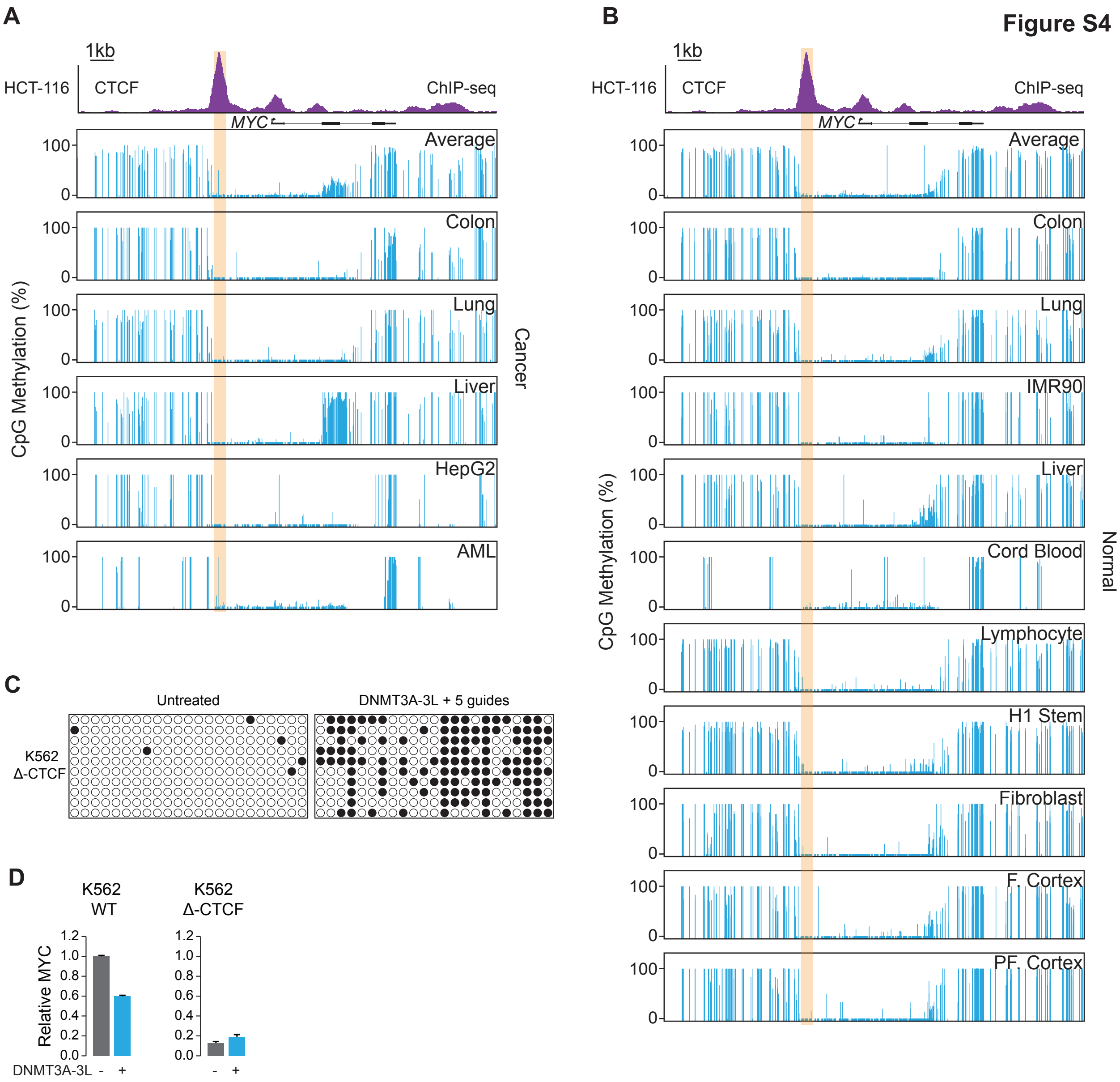


**Figure S4. The *MYC* CTCF loop-anchor site is hypomethylated in a swathe of cancer and normal cells. Loss of *MYC* expression upon docking site methylation is dependent on presence of CTCF. Related to figure 4.**
(**A**) Percentage of methylation of CpG's at the *MYC* locus in cancer cells. Percent methylation of each CpG in the region for which data was available for is represented as a blue line. HCT-116 CTCF ChIP-seq signal is shown in purple with the *MYC* promoter proximal CTCF site highlighted in yellow for reference. ChIP-seq read counts are shown in reads per million sequenced reads per base pair. (**B**) Percentage of methylation of CpG's at the *MYC* locus in normal cells. Percent methylation of each CpG in the region for which data was available for is represented as a blue line. Whole genome bisulfite sequencing data from ENCODE, (Barabé et al., 2016; Ziller et al., 2013).
(**C**) Methylation at *MYC* promoter loop-anchor site in untreated and dCas9-DNMT3A-3L + 5 gRNA transfected K562 Δ-CTCF cells. (**D**) qPCR analysis of *MYC* mRNA levels for untreated and dCas9-DNMT3A-3L + 5 gRNA transfected K562 WT cells (data from Figure 4D) and K562 Δ-CTCF cells. Error bars represent the standard deviation of the mean for three biological replicates.

**Figure S5**

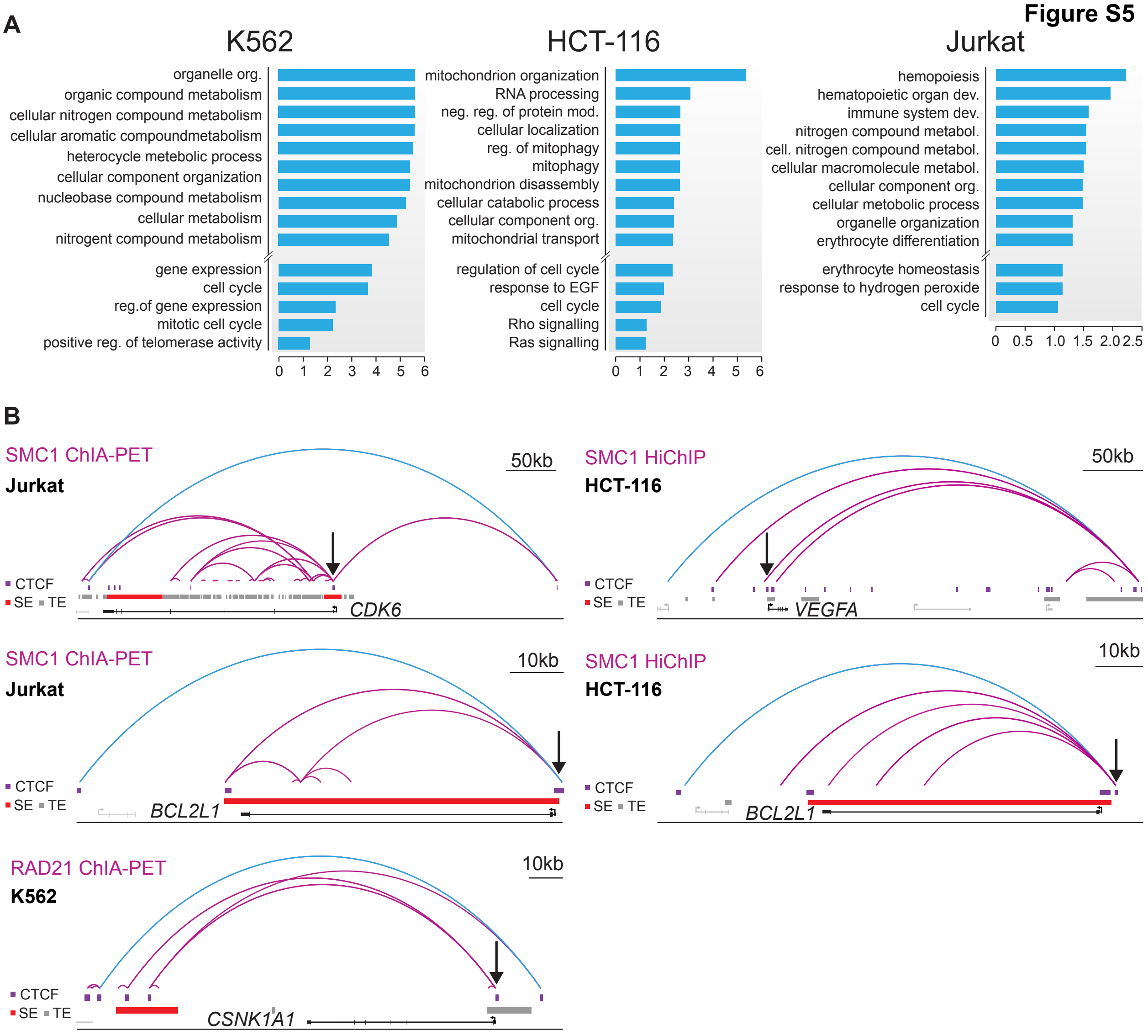


**Figure S5. Putative enhancer-docking sites occur at additional genes with prominent roles in cancer. Related to figure 5.**

(**A**) Gene ontology analysis of the genes with putative CTCF enhancer-docking in K562, HCT-116 and Jurkat cells. Blue bars indicate the –log10 of the q-value associated with the enrichment of the GO term indicated. The top ten most enriched GO terms are shown in addition to selected cancer associated GO terms that are significantly enriched (q-value < 0.1). (**B**) Displays of additional cancer associated genes with putative CTCF enhancer-docking. Black arrows indicate the CTCF sites that may facilitate enhancer-docking. CTCF ChIP-seq peaks are shown as purple rectangles, typical enhancers are shown as grey rectangles and super-enhancers are shown as red rectangles. The *MYC* insulated neighborhood loop is shown in blue and loops internal to it are shown in purple. ChIA-PET data used is indicated in purple lettering.

# Supplemental Experimental Procedures

## CONTACT FOR REAGENT AND RESOURCE SHARING

Further information and requests for resources and reagents should be directed to and will be fulfilled by the Lead Contact, Rick Young (young@wi.mit.edu). The Whitehead Institute has filed a patent application based on the work described in this manuscript.

### RESOURCE TABLE

| REAGENT or RESOURCE | SOURCE | IDENTIFIER |
|---|---|---|
| Antibodies | | |
| H3K27Ac | Abcam | 4729 |
| SMC1 | Bethyl | A300-055A |
| CTCF | Millipore | 07729 |
| Beta-Actin | Sigma | A5441 |
| Chemicals, Peptides, and Recombinant Proteins | | |
| Doxycycline | Sigma Aldrich | D9891 |
| polybrene | Millipore | TR-1003 |
| Critical Commercial Assays | | |
| EpiTect Bisulfite Kit | Qiagen | 59104 |
| TruSeq DNA Sample Preparation v2 Kit | Illumina | RS-122-2001 |
| Nextera DNA Library Preparation Kit | Illumina | FC-121-1030 |
| Expand Long Template Polymerase | Roche | 11759060001 |
| RNeasy plus | Qiagen | 74134 |
| TaqMan Universal PCR Master Mix | Applied Biosystems | 4304437 |
| Power SYBR Green Master Mix | Applied Biosystems | 4368577 |
| Deposited Data | | |
| 4C | This study | GSE92880 |
| HIChIP | This study | GSE92881 |
| ChIP-seq | This study | GSE92879 |
| Experimental Models: Cell Lines | | |
| HCT-116 | ATCC | CCL-243 |
| K562 | ATCC | HTB-22 |
| MCF7 | ATCC | CCL-247 |
| Jurkat | ATCC | TIB-152 |
| HEK293T | ATCC | CRL-3216 |
| Oligonucleotides | | |
| See table S4 | | |
| Recombinant DNA | | |
| dCAS9-DNMT3A-3L | This study | |
| px330-Cas9 | Zhang Lab | Addgene: 42230 |
| DNMT3A-3L | Jeltsch Lab | |
| pLentiGuidePuro | | Addgene: 52963 |
| pAW21 | This study | |
| pAW22 | This study | |

| | | |
|---|---|---|
| psPAX | Didier Trono | Addgene 12260 |
| pMD2.G | Didier Trono | Addgene 12259 |
| Software and Algorithms | | |
| Origami | This study/ (Weintraub et al., 2017) | https://github.com/younglab/origami |
| ROSE | (Whyte et al., 2013) | https://bitbucket.org/young_computation/rose |
| 4C Fourfold | This Study | https://github.com/younglab/fourfold |
| Bowtie | (Langmead et al., 2009) | http://bowtiebio.sourceforge.net/index.shtml |
| Samtools | (Li et al., 2009) | http://samtools.sourceforge.net |
| MACS | (Zhang et al., 2008) | http://liulab.dfci.harvard.edu/MACS/index.html |
| BEDTools | (Quinlan and Hall, 2010) | http://bedtools.readthedocs.io |
| UCSC Genome Browser | (Kent et al., 2002) | http://genome.ucsc.edu/cgibin/hgGateway |
| WASHU EpiGenome browser | (Zhou et al., 2011) | http://epigenomegateway.wustl.edu/ |
| 3D Genome viewer | (Wang et al., 2017) | http://www.3dgenome.org |
| Kallisto | (Bray et al., 2016) | https://pachterlab.github.io/kallisto/ |

## EXPERIMENTAL MODEL AND SUBJECT DETAILS

### Cell Lines

K562 (female), MCF7 (female), HCT-116 (male) and Jurkat (male) cells were purchased from ATCC (CCL-243, HTB-22, CCL-247, TIB-152) and propagated according to ATCC guidelines in RPMI-1640 with GlutaMax (Life Technologies 61870-127) or DMEM, high glucose, pyruvate (Life Technologies 11995-073), supplemented with 10% fetal bovine serum (Sigma). Cells were maintained at 37°C and 5% $CO_2$.

## METHOD DETAILS

### CRISPR/Cas9 genome editing

Genome editing was performed using CRISPR/Cas9 essentially as described (Ran et al., 2013; Shalem et al., 2014). The genomic sequences complementary to all guide RNAs are listed in Table S4

For generation of the *MYC* CTCF site deletion clones, target-specific oligonucleotides were cloned into the pX330 plasmid which carries a U6 promoter, chimeric guide RNA, and a codon-optimized version of Cas9. pX330 was a kind gift of F. Zhang (Ling et al., 2012) (Addgene: 42230). Using this construct, 2 million cells (expressing the exogenous

copy of *MYC*) were transfected with 10 ug of DNA with 50 uL of 1mg/ml PEI and sorted for presence of GFP after 2 days. Individual cells were then propagated in to clonal lines.

For the inducible CRISPR/Cas9 CTCF perturbation experiments (Figure S2C-G), target specific oligonucleotides were cloned into a plasmid containing the chimeric RNA, a doxycycline inducible H1 promoter, TetR, and a selectable marker (pAW 21 or pAW 22). Three separate viruses were produced containing pAW21::up guide, pAW22::down guide, and pLentiCas9-blast (Addgene: 52962, a kind gift of F. Zhang (Sanjana et al., 2014)). Stable cell lines were generated (see section on virus production and cell line generation) and then genome editing was induced by the addition of doxycycline (Sigma Aldrich D9891) at 1 ug/mL. Cells were induced for 72 hours and every 24 hours fresh doxycycline was spiked in.

<u>Virus production and generation of cell lines</u>

For virus production, HEK293T cells were grown to 50-75% confluency on a 15 cm dish and then transfected with 15 µg plasmid of interest, 11.25 µg psPAX (Addgene 12260), and 3.75 µg pMD2.G (Addgene 12259). psPAX and pMD2.G were kind gifts of Didier Trono. After 12 hours, media was replaced. Viral supernatant was collected 24 hours after media replacement (36 hrs post transfection) and fresh media was added. Viral supernatant was collected again 48 hours after the media replacement (60 hours post transfection). Viral supernatant was cleared of cells by either centrifugation at 500 x g for 10 minutes or filtration through a 0.45-micron filter. The virus was concentrated with Lenti-X concentrator (Clonetech 631231) per manufacturers' instruction. Concentrated virus was resuspended in either DMEM or RPMI (depending on the cell line being infected) and added to 5 million cells in the presence of polybrene (Millipore TR-1003) at 8 ug/mL. After 24 hours, viral media was removed and fresh media containing drug was added. Drug concentrations are as follows: Puromycin (Thermo A1113802) (2 ug/mL), Geneticin (Thermo 10131027) (800 ug/mL), Blasticidin (Invivogen ant-bl-1) (10 ug/mL). Cells were selected until all cells on non-transduced plates died.

<u>RNA isolation and quantitative RT-PCR</u>

RNA was isolated using the RNeasy, RNeasy plus or AllPrep kit (QIAGEN 74004, 80204) and reverse transcribed using oligo-dT primers (Promega C1101) and SuperScript III reverse transcriptase (Thermo 18080093) according to the manufacturers' instructions. Quantitative real-time PCR was performed on a 7000 AB Detection System using Taqman probes for *MYC* (Hs00153408_m1) and GAPDH (Hs02758991_g1) in conjunction with Taqman 2x master mix (Thermo 4304437). For detection of endogenous *MYC* only in experiments utilizing cells expressing the exogenous copy of *MYC*, primers specific to the endogenous copy of *MYC* (Table S4) were designed against a *MYC* 3' UTR region not present in the exogenous construct and qPCR was conducted with SYBR green PCR master mix (Thermo 4309155).

<u>ChIP-seq</u>

ChIP was performed as described in (Lee et al., 2006) with a few adaptations. ~30 million K562 cells were crosslinked for 10 min at room temperature by the addition of one-tenth of the volume of 11% formaldehyde solution (11% formaldehyde, 50 mM HEPES pH 7.3, 100 mM NaCl, 1 mM EDTA pH 8.0, 0.5 mM EGTA pH 8.0) to the growth media followed by 5 min quenching with 125 mM glycine. Cells were washed twice with PBS, then the supernatant was aspirated and the cell pellet was flash frozen at -80C. 100µl of Protein G Dynabeads (Thermo 10003D) were blocked with 0.5%BSA (w/v) in PBS. Magnetic beads were bound with 40 µl of anti-CTCF antibody (Millipore 07-729). Nuclei were isolated as previously described (Lee et al., 2006), and sonicated in lysis buffer (20 mM Tris-HCl pH 8.0, 150 mM NaCl, 2 mM EDTA pH8.0, 0.1% SDS, and 1% Triton X-100) on a Misonix 3000 sonicator for 5 cycles at 30s each on ice (18-21 W) with 60 s on ice between cycles. Sonicated lysates were cleared once by centrifugation and incubated overnight at 4°C with magnetic beads bound with antibody to enrich for DNA fragments bound by the indicated factor. Beads were washed with wash buffer A (50 mM HEPES-KOH pH7.9, 140 mM NaCl, 1 mM EDTA pH 8.0, 0.1% Na-Deoxycholate, 1%Triton X-100, 0.1% SDS), B (50 mM HEPES-KOH pH7.9, 500 mM NaCl, 1 mM EDTA pH 8.0,0.1% Na-Deoxycholate, 1% Triton X-100, 0.1% SDS), C (20 mM Tris-HCl pH8.0, 250 mMLiCl, 1 mM EDTA pH 8.0, 0.5% Na-Deoxycholate, 0.5% IGEPAL C-630 0.1% SDS) and D (TE with 50 mM NaCl) sequentially. DNA was eluted in elution buffer (50 mM Tris-HCL pH 8.0, 10mM EDTA, 1% SDS). Cross-links were reversed overnight at 65°C. RNA and protein were digested using RNase A and Proteinase K, respectively and DNA was purified with phenol chloroform extraction and ethanol precipitation. Purified ChIP DNA was used to prepare Illumina multiplexed sequencing libraries. Libraries for Illumina sequencing were prepared following the Illumina TruSeq DNA Sample Preparation v2 kit. Amplified libraries were size-selected using a 2% gel cassette in the Pippin Prep system from Sage Science set to capture fragments between 200 and 400 bp. Libraries were quantified by qPCR using the KAPA Biosystems Illumina Library Quantification kit according to kit protocols. Libraries were sequenced on the Illumina HiSeq 2500 for 40 bases in single read mode.

4C-seq

A modified version of 4C-seq (van de Werken et al., 2012a, 2012b) was developed. The major change was the ligation is performed in intact nuclei (in situ). This change was incorporated because previous work has noted that in situ ligation dramatically decreases the rate of chimeric ligations and background interactions (Nagano et al., 2015; Rao et al., 2014).

Approximately 5 million K562 cells were resuspended in 5 mL 10% FBS/PBS. 5 mL of 4% formaldehyde in 10% FBS/PBS was added and cells were crosslinked for 10 minutes while rotating at room temperature. Glycine was added to a final concentration of 0.125 M and cells were centrifuged at 300xg for 5 minutes. Cells were washed twice with PBS, transferred to an eppendorf tube, snap frozen and stored at -80. Pellets were gently resuspended in Hi-C lysis buffer (10 mM Tris-HCl pH 8, 10 mM NaCl, 0.2%

Igepal) with 1x protease inhibitors (Roche, 11697498001). Cells were incubated on ice for 30 minutes then washed once with 500 uL of ice-cold Hi-C lysis buffer with no protease inhibitors. Pellets were resuspended in 50 uL of 0.5% SDS and incubated at 62°C for 7 minutes. 145 uL of water and 25 uL of 10% Triton X-100 were added and tubes incubated at 37°C for 15 minutes. 25 uL of 10X New England Biolabs CutSmart buffer and 200 units of NlaIII (NEB R0125L) enzyme were added and the chromatin was digested for four hours at 37 degrees in a thermomixer at 500 RPM. 200 additional units of NlaIII was spiked in and digest continued for 12 hours. Then, 200 additional units of NlaIII was spiked in and digest continued for four more hours. Restriction enzyme was inactivated by heating to 62°C for 20 minutes while shaking at 500 rpm. Proximity ligation was performed in a total of 1200 uL with 2000 units of T4 DNA ligase (NEB M0202M) for six hours at room temperature. After ligation samples were spun down for 5 minutes at 2500 g and resuspended in 300 uL 10 mM Tris-HCl, 1% SDS and 0.5 mM NaCl with 1000 units of Proteinase K. Samples were reversed cross-linked overnight at 68°C.

Samples were then phenol-chloroform extracted and ethanol precipitated and the second digestion was performed overnight in 450 uL with 50 units of CviQI (NEB R0639L). Samples were phenol-chloroform extracted and ethanol precipitated and the second ligation was performed in 14 mL total with 6700 units of T4 DNA ligase (NEB 0202M) at 16°C overnight. Samples were ethanol precipitated, resuspended in 500 uL Qiagen EB buffer, and purified with a Qiagen PCR kit.

The concentration was measured with a Nanodrop and PCR amplification was performed with 16 50 uL PCR reactions using Roche Expand Long Template polymerase (Roche 11759060001). Reaction conditions are as follows: 11.2 uL Roche Expand Long Template Polymerase, 80 uL of 10 X Roche Buffer 1, 16 uL of 10 mM dNTPs (Promega PAU1515), 112 uL of 10 uM forward primer, 112 uL of 10 uM reverse primer (Table S4), 200 ng template, and milli-q water till 800 uL total. Reactions were mixed and then distributed into 16 50 uL reactions for amplification. Cycling conditions were a "Touchdown PCR" based on reports that this decreases non-specific amplification of 4C libraries (Ghavi-Helm et al., 2014). The conditions are: 2' 94°C, 10'' 94°C, 1' 63°C, 3' 68 °C, repeat steps 2-4 but decrease annealing temperature by one degree, until 53°C is reached at which point reaction is cycled an additional 15 times at 53°C, after 25 total cycles are performed the reaction is held for 5' at 68°C and then 4°C. Libraries were cleaned-up using a Roche PCR purification kit (Roche 11732676001) using 4 columns per library. Reactions were then further purified with Ampure XP beads (Agencourt A63882) with a 1:1 ratio of bead solution to library following the manufactures instructions. Samples were then quantified with Qubit and the KAPA Biosystems Illumina Library Quantification kit according to kit protocols. Libraries were sequenced on the Illumina HiSeq 2500 for 40 bases in single read mode.

HiChIP

HiChIP was performed essentially as described (Mumbach et al., 2016). 10 million HCT116 cells were crosslinked for 10 min at room temperature by the addition of one-tenth of the volume of 11% formaldehyde solution (11% formaldehyde, 50 mM HEPES pH 7.3, 100 mM NaCl, 1 mM EDTA pH 8.0, 0.5 mM EGTA pH 8.0) to the growth media followed by 5 min quenching with 125 mM glycine. Cells were washed twice with PBS, then the supernatant was aspirated and the cell pellet was flash frozen in liquid nitrogen. Frozen crosslinked cells were stored at −80°C.

The crosslinked pellets were thawed on ice, resuspended in 500 μL of ice-cold Hi-C Lysis Buffer (10mM Tris-HCl pH 8.0, 10 mM NaCl, 0.2% NP-40 with protease inhibitors), and rotated at 4ºC for 30 minutes. Nuclei were spun down at 2500 rcf for 5 minutes at 4ºC, and washed once with 500 μL of ice-cold Hi-C Lysis Buffer. Supernatant was removed, and the pellet was resuspended in 100 μL of 0.5% SDS. Nuclei were incubated at 62ºC for 7 minutes, and SDS was quenched by addition of 285 μL of $H_2O$ and 50 μL of 10% Triton X-100 for 15 minutes at 37ºC. After the addition of 50 μL of 10X NEB Buffer 2 and 400U of MboI restriction enzyme (NEB, R0147), chromatin was digested overnight at 37ºC. The following day, the MboI enzyme was inactivated by incubating the nuclei at 62ºC for 20 minutes.

To fill in the restriction fragment overhangs and mark the DNA ends with biotin, the following was added: 37.5 μL 0.4 mM biotin-ATP (19524-016, Invitrogen) 1.5 μL of 10 mM dCTP (N0441S, NEB), 1.5 μL of 10 mM dTTP (N0443S, NEB), 1.5 μL of 10 mM dGTP (N0442S, NEB), 10 μL of 5U/μL DNA Polymerase I, Large (Klenow) Fragment (NEB, M0210), and the tubes were incubated at 37ºC for 1 hour with rotation. Next, the following mix was added for the proximity ligation step: 150 μL of 10X NEB T4 DNA ligase buffer with 10 mM ATP (NEB, B0202), 125 μL 10% Triton X-100, 3 μL 50 mg/mL BSA, 10 μL 400 U/μL T4 DNA Ligase (NEB, M0202), 660 μL $H_2O$, and the nuclei suspension was incubated at room temperature for 6 hours with rotation. Nuclei were pelleted at 2500 rcf for 5 minutes and supernatant was removed.

Pellets were resuspended in 880 μL in Nuclear Lysis Buffer (50mM Tris-HCl pH 7.5, 10 mM EDTA, 1% SDS with protease inhibitors), and the lysates were sonicated on a Covaris S220 instrument using the following parameters: Fill Level 10, Duty Cycle 5, PIP 140, Cycles/Burst 200, for 4 minutes. Sonicated lysates were spun down at 16100 rcf for 15 minutes at 4oC, and the supernatant was transferred to a fresh tube. The supernatant was split into two Eppendorf tubes (about 400 μL of lysate in each), and 800 μL of ChIP dilution buffer (0.01% SDS, 1.1% Triton X-100, 1.2 mM EDTA, 16.7 mM Tris pH 7.5, 167 mM NaCl) was added to each tube. 60 μL of Protein G beads (Life Technologies) were washed in ChIP dilution buffer, resuspended in 100 μL ChIP dilution buffer and 50 μL were added to each of the two tube of lysates. Tubes were then rotated for 1 hour at 4ºC to preclear the lysates. Dynabeads were separated on a magnetic stand, and the supernatant was moved to a fresh tube. 3.5 μg of SMC1A antibody (Bethyl A300-055A) were added to each tube, and tubes were incubated at 4ºC overnight with rotation. The next day, 60 μL of Protein G beads were washed ChIP Dilution Buffer, resuspended in 100 μL ChIP Dilution Buffer, and 50 μL was added to

each sample tube. Samples were then incubated for 2 hours at 4°C with rotation. Beads were washed twice with Low Salt Wash Buffer (0.1% SDS, 1% Triton X-100, 2 mM EDTA, 20 mM Tris-HCl pH 7.5, 150 mM NaCl), twice with High Salt Wash Buffer (0.1% SDS, 1% Triton X-100, 2 mM EDTA, 20 mM Tris-HCl pH 7.5, 500 mM NaCl), twice with LiCl buffer (10 mM Tris pH 7.5, 250 mM LiCl, 1% NP-40, 1% Sodium-deoxycholate, 1 mM EDTA). Beads were then resuspended in 100 µL of DNA Elution Buffer (50 mM $NaHCO_3$, 1% SDS), incubated for 10 minutes at room temperature with rotation and 3 minutes at 37°C with shaking. Beads were separated on a magnetic stand, and supernatant was transferred to a fresh tube. Beads were then mixed with another 100 µL of DNA Elution Buffer, incubated for 10 minutes at room temperature with rotation and 3 minutes at 37°C with shaking. Beads were separated on a magnetic stand, and supernatant was combined with the previous round of supernatant. 10 µL of Proteinase K (20mg/ml) was added to each sample and samples were incubated at 55°C for 45 minutes with shaking. Temperature was then increased to 67°C, and samples were incubated for 1.5 hours with shaking. Samples were purified on a Zymo column (Zymo Research).

Fragmentation of the ChIP DNA was performed using the Tn5 transposase (Illumina). First, 5 µL of Streptavidin M-280 magnetic beads were washed with Tween Wash Buffer (5 mM Tris-HCl pH 7.5, 0.5 mM EDTA, 1 M NaCl, 0.05% Tween-20), resuspended in 10 µL of Binding Buffer (10 mM, Tris-HCl pH 7.5, 1 mM EDTA, 2 M NaCl), and added to the samples. Samples were then rotated for 15 minutes at room temperature. Beads were separated on a magnet, and supernatant was discarded. Beads were washed twice with 500 µL of Tween Wash Buffer and incubated at 55°C for 2 minutes shaking. Beads were then washed with 100 µL of 1X TD Buffer (Nextera DNA sample preparation kit, FC-121-1030, Illumina), and tagmented using the Nextera DNA sample preparation kit (FC-121-1030, Illumina). 0.5 µL TDE1 enzyme was used to tagment 10ng of ChIP DNA (quantified after the previous Zymo column purification). Tagmentation was performed for 10 minutes at 55°C with shaking. Beads were then separated on a magnet, and supernatant was discarded. Beads were washed with 50 mM EDTA at 50°C for 30 minutes, and twice with 50 mM EDTA at 50°C for 3 minutes. Beads were then washed twice in Tween Wash Buffer at 55°C for 2 minutes, and once with 10 mM Tris for 1 minute at room temperature. The tagmented library still bound to the beads was amplified by 12 cycles of PCR using the Nextera DNA sample preparation kit. The library was then purified on a Zymo column, size-selected (300-700 bp) using AMPure beads (Agencourt) per manufacturers' instructions, and sequenced 100x100 on an Illumina Hi-Seq 2500.

<u>Targeted methylation and bisulfite sequencing.</u>

To perform targeted methylation, HCT-116 cells or K562 cells were transfected with a dCas9-DNMT3A-3L construct and 5 guides. To generate the dCas9-DNMT3A-3L construct, dCas9 was isolated from pSQL1658 (Addgene: 51023) by PCR. Cas9 was removed from pX330-Cas9 (Addgene: 42230) by AgeI and EcoRI restriction digest.

dCas9 was inserted into pX330 to create pX330-dCas9. DNMT3A-3L carrier plasmid was a generous gift from the Jeltsch lab (Siddique et al., 2013). DNMT3A-3L was cloned into pX330-dCas9 using PmeI and AscI sites to create pX330-dCas9-DNMT3A-3L (no guides). Guide RNAs were added to pX330-dCas9-DNMT3A-3L by digesting pX330-dCas9-DNMT3A-3L with BbsI followed by ligation of annealed oligos (Table S4) to create pX330-dCas9-DNMT3A-3L-guide with three different guides. An additional plasmid containing two guides, lentiGuide-Puro-double_guide, was also generated. Double guide containing gBlock (individual guide sequences Table S4) was cloned into lentiGuide-Puro (Addgene: 52963) as described (Vidigal and Ventura, 2015) to create lentiGuide-Puro-double_guide. Two hundred fifty thousand HCT-116 cells were transfected with 250ng of pX330-dCas9-DNMT3A-3L-guide1, 250ng of pX330-dCas9-DNMT3A-3L-guide2, 250ng of pX330-dCas9-DNMT3A-3L-guide3, 250ng lentiGuide-Puro-double_guide, and 5 uL of 1mg/ml PEI, and harvested after two days. One million K562 cells were transfected with 2.5µg of pX330-dCas9-DNMT3A-3L-guide1, 2.5µg of pX330-dCas9-DNMT3A-3L-guide2, 2.5µg of pX330-dCas9-DNMT3A-3L-guide3, and 2.5µg lentiGuide-Puro-double_guide using the Neon transfection system (Invitrogen MPK10025) following the manufacturers protocol and electroporation settings as follows: 1450V, 10ms width, 3 pulses. HCT-116 and K562 cells were harvested and sorted for GFP presence after two days.

To detect methylation, 2ug of gDNA from HCT-116 or K562 cells untransfected or transfected with dCas9-DNMT3A-3L plus guides were bisulfite converted using the EpiTect Bisulfite Kit (QIAGEN 59104). Converted gDNA was eluted in 20uL $H_2O$. Converted gDNA was PCR amplified with EpiMark® Hot Start Taq DNA Polymerase (NEB M0490) using 3uL of converted gDNA as template and locus specific primers (table S4). PCR was carried out as follows: 95°C for 30 sec; 95°C for 20 sec; 52°C for 30 sec; 68°C for 30 sec; repeat steps 2-4 45x; 68°C for 5 min; Hold 4°C. Resultant amplicons were cleaned up using QIAquick PCR Purification Kit (QIAGEN 28106) and eluted in 20ul $H_2O$. Clean amplicons (3uL) were subcloned using the pGEM-T Easy vector system (Promega A1360) and transformed into DH5α competent cells. Individual colonies were then picked, and colony PCR was carried out using GoTaq Green Master Mix (Promega M712) with the same primers originally used for the converted gDNA amplification. Colony PCR was carried out as follows: 95°C for 2 min; 95°C for 45 sec; 51°C for 45 sec; 72°C for 45 sec; repeat steps 2-4 29x; 72°C 5 min; Hold 4°C. Resultant amplicons were then Sanger sequenced, and CpG methylation was detected as CpG sequences that were not converted to TpG. All converted DNA analyzed had greater than or equal to 95% bisulfite conversion rate.

Hi-C visualization

Hi-C datasets were visualized using the 3D Genome browser at http://www.3dgenome.org.

Visualization of ChIA-PET interactions on the WashU Genome Browser

The output of origami was visualized in the WashU genome browser by converting the output of origami into a WashU compatible format using *origami-conversion.*

Topoloogically Associating Domain (TAD) calls
TAD calls were taken from the TAD calls in (Dixon et al., 2012) from the H1 human embryonic cell line.

## QUANTIFICATION AND STATISTICAL ANALYSIS

ChIP-seq data analysis
ChIP-Seq datasets were generated for this study as well as collated from previous studies (Table S5), and were aligned using Bowtie (version 0.12.2) (44) to the human genome (build hg19, GRCh37) with parameter -k 1 -m 1 -n 2. We used the MACS version 1.4.2 (model-based analysis of ChIP-seq) (45) peak finding algorithm to identify regions of ChIP-seq enrichment over input DNA control with the parameter "--no-model --keep-dup=auto". A p-value threshold of enrichment of 1e-09 was used. UCSC Genome Browser tracks were generated using MACS wiggle outputs with parameters "-w –S – space=50". The browser snapshots of the ChIP-Seq binding profiles displayed throughout the study use read per kilobase per million mapped reads dimension (rpm/bp) on the y-axis.

Identification of enhancers and super-enhancers
Enhancers and super-enhancers were identified using H3K27Ac ChIP-seq data as previously described (Hnisz et al., 2013). Briefly, enhancers were defined as H3K27Ac ChIP-Seq peaks identified using MACS. To identify super-enhancers, the H3K27Ac ChIP-Seq peaks (i.e. enhancers) were stitched together if they were within 12.5 kb, and the stitched enhancers were ranked by their ChIP-seq read signal of H3K27Ac, using the ROSE algorithm (https://bitbucket.org/young_computation/rose) (Lovén et al., 2013). ROSE separates super-enhancers from typical enhancers by identifying an inflection point of H3K27ac signal vs. enhancer rank (Hnisz et al., 2013; Lovén et al., 2013).

4C analysis
The 4C-seq samples were first processed by removing their associated read primer sequences (Table S4) from the 5' end of each FASTQ read. To improve mapping efficiency of the trimmed reads by making the read longer, the restriction enzyme digest site was kept on the trimmed read. After trimming the reads, the reads were mapped using bowtie with options –k 1 –m 1 against the hg19 genome assembly. All unmapped or repetitively mapping reads were discarded from further analysis. The hg19 genome was then "digested" in silico according to the restriction enzyme pair used for that sample to identify all the fragments that could be generated by a 4C experiment given a restriction enzyme pair. All mapped reads were assigned to their corresponding fragment based on where they mapped to the genome. The digestion of a sample in a 4C experiment creates a series of "blind" and "non-blind" fragments as described (van de Werken et al., 2012b). In a perfect experiment, we should have only observed reads

at non-blind fragments, and reads at blind fragments exhibit a much higher experimental variability than non-blind fragments, so we only used the reads from non-blind fragments for further analysis. To normalize the distribution of different samples, we quantile normalized all non-blind fragments in each sample together. If no reads were detected at a non-blind fragment for a given sample when reads were detected in at least one other sample, we assigned a “0” to that non-blind fragment for the sample(s) missing reads. After normalization, we then smoothed the normalized profile of each sample using a 6kb running mean at 500 bp steps across the genome. After smoothing, for each condition we combined the replicates of a condition by taking the mean signal of each bin across all replicates of the condition. Quantification of the 4C signal counted the reads per fragment per million sequenced reads in the indicated regions. For quantification the H3K27Ac stitched peaks from the ROSE algorithm were used (Hnisz et al., 2013) or the CTCF MACS peak calls, padded with 1kb on either side in order to include sufficient 4C fragments.

HiChIP data analysis

The HiChIP samples were processed by removing their associated read primer sequences from the 5’ end of each FASTQ read. Read pairs were separated and separate reads were mapped using bowtie with options –k 1 –m 1 against the hg19 genome assembly. Al unmapped or repetitively mapping read were discarded from further analysis. The hg19 genome was then divided in 50 kb bins and reads were joined back together in pairs (Paired End Tag PET). For every pair of bins the number of PETS joining them was then calculated. These data were then further analyzed by the ORIGAMI pipeline to identify significant bin to bin interaction pairs.

ChIA-PET data analysis and ORIGAMI description

We development a new software pipeline and analytical method called *origami* to process ChIA-PET. The software and releases can be found at https://github.com/younglab/origami using version alpha20160828. The ChIA-PET data sets analyzed along with their corresponding linker sequence and called interactions in and around the *MYC* TAD can be found in Table S4. Each ChIA-PET data sets was processed as follows: the reads were first trimmed and aligned using *origami-alignment,* which trims the ChIA-PET linker if present and aligns trimmed PETs. PETs not having a linker were discarded from further analysis. Each end of a PET with a linker sequence were separately mapped to the hg19 genome assembly using bowtie with the following options: -v 1 –k 1 –m 1. After alignment, the separated PETs were re-paired in the final BAM output. After repairing, all duplicated PETs within the data were removed, since these were believed to be PCR duplicates. Peaks were called on the re-paired ChIA-PET reads using MACS1 v1.4.2 with the following parameters: --nolambda –nomodel –p 1e-9.

We developed a novel analytical method to analyze ChIA-PET data that used a two-component Bayesian mixture model to accurately identify *in vivo* interactions from the

ChIA-PET data by accurately estimating the difference between the biological signal and technical and biological noise by controlling for error within the ChIA-PET protocol and linear genomic distance. We defined an *in vivo* interaction as two regions of the genome brought together in the nucleus longer than expected at random given the linear genomic distance between those two region. Our intuition was that true *in vivo* interactions would follow one distribution where experimental noise would arise from a separate distribution, and these two groups could be learned from the data using a mixture model. After alignment, we defined a set of putative contacts where a putative contact was any two MACS1 peaks linked together by at least one mapped PET. The PET count for a putative interaction was the total number of unique PETs mapped at both ends of the putative contact. All putative contacts and their PET count were used in the estimation. We estimated the distributions two-component mixture model (described more below) from these putative contacts using *origami-analysis*. We specifically designed the model to have the second mixture component represent the distribution of the *in vivo* interactions, and we tested whether the estimated group means (described below) were significantly different to validate that model found at least two different groups. After the estimation of each component, for each putative contact we estimate the posterior probability of whether the putative contact was within the distribution of the second component. If this posterior probability was greater than 0.9, we called this putative contact an *in vivo* interaction. We used this threshold because it was a good balance between what be believed to be a high true positive rate while minimizing the false positive rate in each sample analyzed (although we believe that are a few *in vivo* interactions below this threshold as well). In general, we often displayed all putative contacts within the *MYC* TAD by this posterior probability, eliminating the need for a specific cutoff threshold.

For the analytical model, we wanted to build a model that was able to estimate and control for noise arising from the ChIA-PET protocol and linear genomic distance. The linear genomic distance is a potential source of noise in the data because regions of the genome closer together in linear genomic distance are on average more likely to have more frequent interactions by chance than regions of the genome farther away from each other, as observed in Hi-C data. We assume that these two sources of noise are independent Poisson processes from each other (since we are measuring the interaction frequency through PET counts), which appears to be a valid assumption in practice (Phanstiel et al., 2015). With this in mind, the parameters within the model were as follows:

$P_i$ – the count of DNA mapped sequences/reads measured at position $i$, where $i \in \{1..N\}$ and $N$ is the total number of positions measured

$Z_i$— a latent variable having a value of either 0 or 1 measuring whether the measurement in $P_i$ came from a technical artifact (0) or *in vivo* biology (1)

$G_{ij}$ – a latent variable, where $j \in \{0,1\}$, measuring the number of counts observed for sample *i* if were part of component *j*

$B_{ij}$ – a latent variable measuring the number of counts observed for sample *i* if were part of component *j* as a function of the bias due to the genomic distance that sample *i* spans in the genome (where the distance is assumed to be a constant $d_i$ for that sample *i*)

$R_{ij}$ – a latent variable measuring sum of the two independent processes $G_{ij}$ and $B_{ij}$ for sample *i* if were part of component *j*

$\lambda_j$ – a parameter describing the mean of latent variable $G_{.j}$ for all samples, and we guarantee that $\lambda_1 > \lambda_0$to maintain identifiability of each component

$\nu_j(d)$ – a parameter describing the mean of the latent variable $B_{.j}$ at distance *d*

$w_{ij}$ – a parameter describing the binomial probability that sample *I* is part of component *j*

$a_i, b_i$ – a set of constants on the prior distribution of $w_{i1}$ to adjust our prior belief in sample *i* based on our understanding of the biology we have already validated in lab

More specifically, the model is parametrized as follows:

$$\lambda_j \sim Gamma(1,1)$$

$$w_{i1} \sim Beta(a_i, b_i)$$

$$G_j|\lambda_j \sim Poisson(\lambda_j)$$

$$B_{ij}|\nu_{j,}, d_i \sim Poisson(\nu_j|d_i)$$

$$R_{ij} = G_{ij} + B_{ij}|\lambda_j, \nu_j, d_i \sim Poisson(\lambda_j + \nu_j|d_i)$$

$$P_i|R_{ij}, \lambda_j, \nu_j, d_i = \sum_{j \in \{0,1\}} w_{ij} * R_j$$

$$P_i|Z_i = z_i, \lambda_{z_i}, \nu_{z_i}, d_i \sim Poisson\left(\lambda_j + \nu_j \middle| d_i\right)$$

$$w_{i1}|\, Z_i = z_i, P_i \sim Beta(a_i + z_i, b_i + (1 - z_i))$$

And $w_{i0} = 1 - w_{i1}$.

The distribution of the parameters and hyperparameters were simulated by Markov Chain Monte Carlo (MCMC) using either Gibbs sampling or the Metropolis-Hastings algorithm as appropriate. To speed up the simulation between $G_{.j}$ and $B_{ij}$, the $G_{.j}$ parameter is updated first. Then the $B_{ij}$ parameters is updated using the difference between the PETs for $G_{.j}$ and the number of PETs observed for each contact according

to the component they are assigned to in that iteration. Additionally, the mean of $G_0$ is enforced to be less than the mean of $G_1$, although in practice the mean of $G_1$ was always strictly greater than the mean of $G_0$ during the MCMC run so this was never a problem.

The parameter $v_j(d)$ is the mean of the Poisson process estimating the biological bias from the linear genomic distance between the two ends of the putative contact as a function of this distance. To simplify processing, we estimated this function at each iterative using a smoothed cubic spline regression for putative contacts within group *j*. This approximation worked well by generating trends consistent with the power-law decay observed in Hi-C data sets.

The priors $a_{\cdot i}$ and $b_{\cdot i}$ are set to be minimally informative as possible. The $a_{\cdot i}$ hyperparameter is the frequency of the number of contacts sharing one of the same anchors that have a strictly lower measured PET count than the putative contact *i*. The $b_{\cdot i}$ hyperparamer is set to be the frequency of putative contacts sharing the same anchor that have strictly higher number of observed PETs linking the anchors plus the ratio of the multiplication of the depth of reads at both anchors of the putative contact divided by the median depth across all putative contacts floored at 0. We found setting the priors with a non-informative Beta distribution (i.e., $Beta(1,1)$) would also generally call the same *in vivo* interactions but call many more interactions from the putative contacts, where we believed many more of these were artifacts. Hence, we found this minimally informative prior to be more useful for us biologically.

Each run of *origami-analysis* was for 1,000 iterations with a 100 step burn-in period. We chose this number of iterations because the model tended to converge fairly quickly given the complexity of these ChIA-PET data sets. The output of origami is the estimated posterior probability that the putative contact arose from the distribution estimated for the second mixture component, which is assumed to model *in vivo* interactions within the ChIA-PET experiment. Accordingly, putative contact with a posterior probability closer to 1 are believed to be more likely to be *in vivo* interactions.

<u>CTCF motif analysis</u>
CTCF motifs were called in the human and mouse genomes (using hg19 and mm9 assemblies, respectively) using *fimo* (Grant et al., 2011). The CTCF motif from the JASPAR CORE 2014 database was used. The *fimo* p-value threshold was set to 1e-2 and the max-stored-scores parameter set to 100000000. To rank the importance of individual CTCF motifs, the motifs within the targeted CTCF peak upstream of *MYC* were ranked by their score within the score column in the GFF output of fimo.

<u>Identification of genes with a putative enhancer-docking site</u>
Genes with a putative enhancer-docking site were identified by filtering the list of all 26,801 annotated genes down to those that were expressed to a level of 1 transcript per million based on available expression data for HCT-116, Jurkat and K562 cells (Table S4). CTCF ChIP-seq was then used to filter for genes that had a CTCF peak within 2.5

kilobase of an active TSS. Interaction data was then used to score if active TSSs with CTCF occupancy were interacting with an active enhancer. Enhancers were called using H3K27Ac data from each corresponding cell line. TSSs that qualify these conditions are reported in Table S2. Loop anchors from the interaction data that were overlapping with enhancers were used to identify all CTCF ChIP-seq peaks that could be involved in the anchoring on the enhancer side of the loops. The enhancer-promoter loop anchors, gene name, transcriptions start site, CTCF peaks, the JASPAR score off the strongest motif and the orientation of the strongest motif are included Table S6.

Gene Ontology (GO) analysis

We took all putative enhancer-docking site genes per cell type. We ran each gene set through the GO analysis against all GENCODE genes to find enriched biological processes. Using the topGO and qvalue Bioconductor packages, we tested for enriched terms using Fischer's exact test and correcting for multiple hypothesis testing by converting all p-values into q-values via pFDR. We corrected for multiple hypothesis testing by grouping all biological process, molecular function, and cellular function terms independently. We selected all enriched GO terms with a q-value less than .1 (10% FDR) for further analysis.

Supplementary References

Bray, N.L., Pimentel, H., Melsted, P., and Pachter, L. (2016). Near-optimal probabilistic RNA-seq quantification. Nat. Biotechnol. *34*, 525–527.

Dixon, J.R., Selvaraj, S., Yue, F., Kim, A., Li, Y., Shen, Y., Hu, M., Liu, J.S., and Ren, B. (2012). Topological domains in mammalian genomes identified by analysis of chromatin interactions. Nature *485*, 376–380.

Ghavi-Helm, Y., Klein, F.A., Pakozdi, T., Ciglar, L., Noordermeer, D., Huber, W., and Furlong, E.E.M. (2014). Enhancer loops appear stable during development and are associated with paused polymerase. Nature *512*, 96–9100.

Grant, C.E., Bailey, T.L., and Noble, W.S. (2011). FIMO: Scanning for occurrences of a given motif. Bioinformatics *27*, 1017–1018.

Hnisz, D., Abraham, B.J., Lee, T.I., Lau, A., Saint-André, V., Sigova, A.A., Hoke, H.A., and Young, R.A. (2013). Super-enhancers in the control of cell identity and disease. Cell *155*, 934–947.

Kent, W.J., Sugnet, C.W., Furey, T.S., Roskin, K.M., Pringle, T.H., Zahler, A.M., and Haussler, D. (2002). The human genome browser at UCSC. Genome Res. *12*, 996–1006.

Langmead, B., Trapnell, C., Pop, M., and Salzberg, S.L. (2009). Ultrafast and memory-efficient alignment of short DNA sequences to the human genome. Genome Biol. *10*, R25.

Lee, T., Johnston, S., and Young, R. (2006). Chromatin immunoprecipitation and

microarray-based analysis of protein location. Nat. Protoc. *1*, 729–748.

Li, H., Handsaker, B., Wysoker, A., Fennell, T., Ruan, J., Homer, N., Marth, G., Abecasis, G., and Durbin, R. (2009). The Sequence Alignment/Map format and SAMtools. Bioinformatics *25*, 2078–2079.

Ling, Q., Huang, W., Baldwin, A., and Jarvis, P. (2012). Chloroplast biogenesis is regulated by direct action of the ubiquitin-proteasome system. Science *338*, 655–659.

Lovén, J., Hoke, H.A., Lin, C.Y., Lau, A., Orlando, D.A., Vakoc, C.R., Bradner, J.E., Lee, T.I., and Young, R.A. (2013). Selective inhibition of tumor oncogenes by disruption of super-enhancers. Cell *153*, 320–334.

Mumbach, M.R., Rubin, A.J., Flynn, R.A., Dai, C., Khavari, P.A., Greenleaf, W.J., and Chang, H.Y. (2016). HiChIP: efficient and sensitive analysis of protein-directed genome architecture. Nat. Methods *13*, 919–922.

Nagano, T., Varnai, C., Schoenfelder, S., Javierre, B.-M., Wingett, S., and Fraser, P. (2015). Comparison of Hi-C results using in-solution versus in-nucleus ligation. Genome Biol. *16*, 175.

Phanstiel, D.H., Boyle, A.P., Heidari, N., and Snyder, M.P. (2015). Mango: A bias-correcting ChIA-PET analysis pipeline. Bioinformatics *31*, 3092–3098.

Quinlan, A.R., and Hall, I.M. (2010). BEDTools: A flexible suite of utilities for comparing genomic features. Bioinformatics *26*, 841–842.

Ran, F.A., Hsu, P.D., Wright, J., Agarwala, V., Scott, D.A., and Zhang, F. (2013). Genome engineering using the CRISPR-Cas9 system. Nat. Protoc. *8*, 2281–2308.

Rao, S.S.P., Huntley, M.H., Durand, N.C., Stamenova, E.K., Bochkov, I.D., Robinson, J.T., Sanborn, A.L., Machol, I., Omer, A.D., Lander, E.S., et al. (2014). A 3D map of the human genome at kilobase resolution reveals principles of chromatin looping. Cell *159*, 1665–1680.

Sanjana, N.E., Shalem, O., and Zhang, F. (2014). Improved vectors and genome-wide libraries for CRISPR screening. Nat. Methods *11*, 783–784.

Shalem, O., Sanjana, N.E., Hartenian, E., Shi, X., Scott, D. a, Mikkelsen, T.S., Heckl, D., Ebert, B.L., Root, D.E., Doench, J.G., et al. (2014). Genome-scale CRISPR-Cas9 knockout screening in human cells. Science *343*, 84–87.

Vidigal, J.A., and Ventura, A. (2015). Rapid and efficient one-step generation of paired gRNA CRISPR-Cas9 libraries. Nat. Commun. *6*, 8083.

Wang, Y., Zhang, B., Zhang, L., An, L., Xu, J., Li, D., Choudhary, M.N.K., Li, Y., Hu, M., Hardison, R., et al. (2017). The 3D Genome Browser: a web-based browser for visualizing 3D genome organization and long-range chromatin interactions. bioRxiv.

Weintraub, A.S., Li, C.H., Zamudio, A. V, Sigova, A.A., Hannett, N.M., Day, D.S., Abraham, B.J., Cohen, M.A., Nabet, B., Buckley, D.L., et al. (2017). YY1 Is a Structural Regulator of Enhancer-Promoter Loops. Cell *171*, 1573–1588.e28.

van de Werken, H.J.G., De Vree, P.J.P., Splinter, E., Holwerda, S.J.B., Klous, P., De Wit, E., and De Laat, W. (2012a). 4C technology: Protocols and data analysis (Elsevier Inc.).

van de Werken, H.J.G., Landan, G., Holwerda, S.J.B., Hoichman, M., Klous, P., Chachik, R., Splinter, E., Valdes-Quezada, C., Öz, Y., Bouwman, B.A.M., et al. (2012b). Robust 4C-seq data analysis to screen for regulatory DNA interactions. Nat Methods *9*, 969–972.

Whyte, W.A., Orlando, D.A., Hnisz, D., Abraham, B.J., Lin, C.Y., Kagey, M.H., Rahl, P.B., Lee, T.I., and Young, R.A. (2013). Master transcription factors and mediator establish super-enhancers at key cell identity genes. Cell *153*, 307–319.

Zhang, Y., Liu, T., Meyer, C.A., Eeckhoute, J., Johnson, D.S., Bernstein, B.E., Nussbaum, C., Myers, R.M., Brown, M., Li, W., et al. (2008). Model-based analysis of ChIP-Seq (MACS). Genome Biol. *9*.

Zhou, X., Maricque, B., Xie, M., Li, D., Sundaram, V., Martin, E. a, Koebbe, B.C., Nielsen, C., Hirst, M., Farnham, P., et al. (2011). The Human Epigenome Browser at Washington University. Nat. Methods *8*, 989–990.
