# Supplementary material for: Transcriptional Dysregulation of MYC Reveals Common Enhancer-Docking Mechanism
Source: Cell Rep. Author manuscript; Available in PMC 2018 May 1. (PMC5929158; doi:10.1016/j.celrep.2018.03.056)
Supplement: 8 [file NIHMS961951-supplement-8.pdf]

Cell Reports

Article

Transcriptional Dysregulation of *MYC* Reveals Common Enhancer-Docking Mechanism

Graphical Abstract

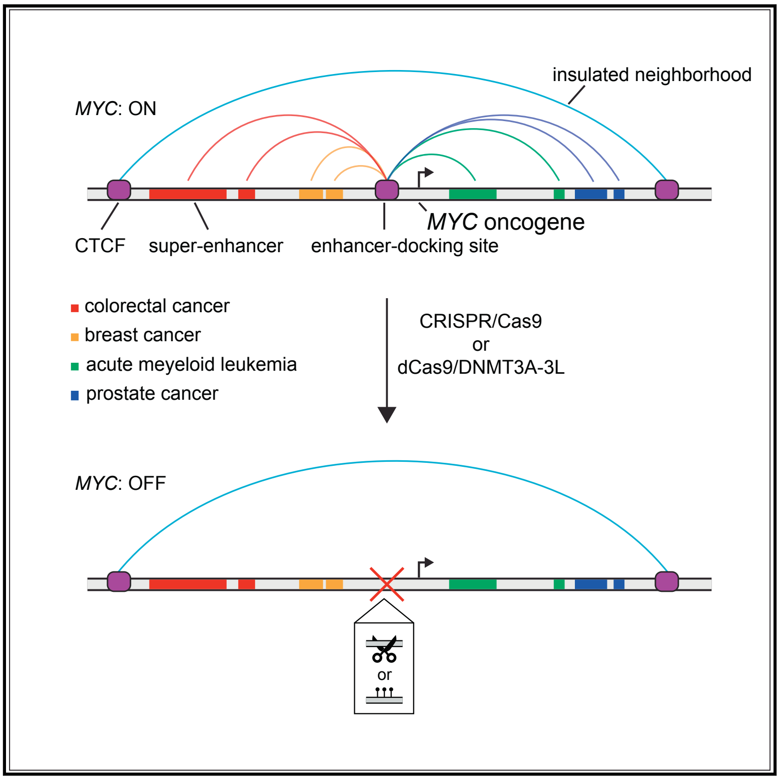

Highlights

- Tumor-specific super-enhancers loop to an enhancer-docking site at the *MYC* oncogene
- Deletion or methylation of *MYC* enhancer-docking site reduces enhancer-promoter looping
- *MYC* enhancer-docking site provides common vulnerability in diverse cancer cells
- Similar docking sites for multiple enhancers occur at additional genes

Authors

Jurian Schuijers,
John Colonnese Manteiga,
Abraham Selby Weintraub, ...,
Denes Hnisz, Tong Ihn Lee,
Richard Allen Young

Correspondence

young@wi.mit.edu

In Brief

Schuijers et al. show that a conserved CTCF site at the promoter of the *MYC* oncogene plays an important role in enhancer-promoter looping with tumor-specific super-enhancers. Perturbation of this site provides a potential therapeutic vulnerability.

Data and Software Availability

GSE92881

Schuijers et al., 2018, Cell Reports *23*, 349–360
April 10, 2018 © 2018 The Author(s).
https://doi.org/10.1016/j.celrep.2018.03.056

CellPress

Cell Reports
Article

OPEN ACCESS
CellPress

Transcriptional Dysregulation of *MYC* Reveals Common Enhancer-Docking Mechanism

Jurian Schuijers,[1,3] John Colonnese Manteiga,[1,2,3] Abraham Selby Weintraub,[1,2] Daniel Sindt Day,[1] Alicia Viridiana Zamudio,[1,2] Denes Hnisz,[1] Tong Ihn Lee,[1] and Richard Allen Young[1,2,4,*]
[1]Whitehead Institute for Biomedical Research, 455 Main Street, Cambridge, MA 02142, USA
[2]Department of Biology, Massachusetts Institute of Technology, Cambridge, MA, 02139, USA
[3]These authors contributed equally
[4]Lead Contact
*Correspondence: young@wi.mit.edu
https://doi.org/10.1016/j.celrep.2018.03.056

SUMMARY

Transcriptional dysregulation of the *MYC* oncogene is among the most frequent events in aggressive tumor cells, and this is generally accomplished by acquisition of a super-enhancer somewhere within the 2.8 Mb TAD where *MYC* resides. We find that these diverse cancer-specific super-enhancers, differing in size and location, interact with the *MYC* gene through a common and conserved CTCF binding site located 2 kb upstream of the *MYC* promoter. Genetic perturbation of this enhancer-docking site in tumor cells reduces CTCF binding, super-enhancer interaction, *MYC* gene expression, and cell proliferation. CTCF binding is highly sensitive to DNA methylation, and this enhancer-docking site, which is hypomethylated in diverse cancers, can be inactivated through epigenetic editing with dCas9-DNMT. Similar enhancer-docking sites occur at other genes, including genes with prominent roles in multiple cancers, suggesting a mechanism by which tumor cell oncogenes can generally hijack enhancers. These results provide insights into mechanisms that allow a single target gene to be regulated by diverse enhancer elements in different cell types.

INTRODUCTION

Elevated expression of the c-MYC transcription factor occurs in a broad spectrum of human cancers and is associated with tumor aggression and poor clinical outcome (Berns et al., 1992; Dang, 2012; Gabay et al., 2014; Grotzer et al., 2001). Activation of the *MYC* gene, which encodes c-MYC, is a hallmark of cancer initiation and maintenance. Dysregulation of *MYC* is often achieved through the formation of large tumor-specific super-enhancers in the region surrounding the *MYC* gene (Chapuy et al., 2013; Fulco et al., 2016; Herranz et al., 2014; Hnisz et al., 2013; Lin et al., 2016; Liu et al., 2015; Lovén et al., 2013; Shi et al., 2013; Whyte et al., 2013; Zhang et al., 2016). These large enhancer clusters differ in size, composition, and distance from the *MYC* promoter, yet all accomplish the same task of stimulating *MYC* overexpression across a broad spectrum of tumors.

Selective gene activation is essential to the gene expression programs that define both normal and cancer cells. During gene activation, transcription factors (TFs) bind enhancer elements and regulate transcription from the promoters of nearby or distant genes through physical contacts that involve looping of DNA between enhancers and promoters (Bonev and Cavalli, 2016; Buecker and Wysocka, 2012; Bulger and Groudine, 2011; Fraser et al., 2015; Müeller-Storm et al., 1989; Spitz, 2016; de Wit et al., 2013). The mechanisms that ensure that specific enhancers interact with specific promoters are not fully understood. Some enhancer-promoter interactions are likely determined by the nature of TFs bound at the two sites (Muerdter and Stark, 2016; Weintraub et al., 2017).

Recent studies have revealed that specific chromosome structures play important roles in gene control. Enhancer-promoter interactions generally occur within larger chromosomal loop structures formed by the interaction of CTCF proteins bound to each of the loop anchors (Dekker and Mirny, 2016; Fraser et al., 2015; Gibcus and Dekker, 2013; Gorkin et al., 2014a; Hnisz et al., 2016a, 2018; Ji et al., 2016). These loop structures, variously called topologically associated domains (TADs), sub-TADs, loop domains, CTCF contact domains, and insulated neighborhoods, tend to insulate enhancers and genes within the CTCF-CTCF loops from elements outside those loops (Dixon et al., 2012; Dowen et al., 2014; Franke et al., 2016; Hnisz et al., 2016a, 2016b; Ji et al., 2016; Narendra et al., 2015; Nora et al., 2012; Phillips-Cremins et al., 2013; Rao et al., 2014). Constraining DNA interactions within CTCF-CTCF loop structures in this manner may facilitate proper enhancer-promoter contacts.

CTCF does not generally occupy enhancer and promoter elements (Cuddapah et al., 2009; Dixon et al., 2012; Dowen et al., 2014; Handoko et al., 2011; Ji et al., 2016; Kim et al., 2007; Phillips-Cremins et al., 2013; Rao et al., 2014; Rubio et al., 2008; Tang et al., 2015). Another TF, YY1, generally binds to enhancers and promoters and facilitates their interaction through YY1 dimerization (Weintraub et al., 2017). However, when CTCF does bind these regulatory elements, it can also contribute to enhancer-promoter interactions (Banani et al., 2017; Nora et al., 2017; Splinter et al., 2006; Zuin et al., 2014).

Here, we investigate DNA looping structures in the *MYC* locus in multiple cancers and identify a CTCF-occupied site at the *MYC* promoter that facilitates docking with essentially any

This is an open access article under the CC BY license (http://creativecommons.org/licenses/by/4.0/).

enhancers that are formed within the 2.8 Mb *MYC* locus. The CTCF-occupied site at the *MYC* promoter, which we call the *MYC* enhancer-docking site, can be abrogated by genetic and epigenetic editing. Similar enhancer-docking sites occur at other oncogenes. This suggests a mechanism by which tumor cells can generally hijack enhancers and, with editing, a potential therapeutic vulnerability.

RESULTS

Cell-Type-Specific *MYC* Enhancers Loop to a Common Upstream CTCF Site

Previous studies have established that tumor cells acquire tumor-specific super-enhancers at various sites throughout the *MYC* locus (Figures 1A and S1A) (Bradner et al., 2017; Chapuy et al., 2013; Gabay et al., 2014; Gröschel et al., 2014; Herranz et al., 2014; Hnisz et al., 2013; Lin et al., 2016; Lovén et al., 2013; Parker et al., 2013; Zhang et al., 2016; Shi et al., 2013), but the mechanisms by which these diverse enhancer structures control *MYC* are not clear. In one case, for example, a super-enhancer located ~2 Mb downstream of the *MYC* gene has been shown to physically interact with *MYC*, but the mechanisms responsible for this specific interaction are unclear (Shi et al., 2013). To gain insights into the potential role of DNA loop structures in gene control at the *MYC* locus, we generated cohesin HiChIP data for HCT-116 cells and collected published DNA interaction data for three other cancer cell types for comparison (Figure 1B; Tables S1 and S5) (Hnisz et al., 2016a; Pope et al., 2014). Among the DNA loop structures observed in these datasets, a large 2.8 Mb DNA loop was evident in all four cell types. This loop connects CTCF sites encompassing the *MYC* gene and qualifies as an insulated neighborhood. The DNA anchor sites of this 2.8 Mb DNA loop occur at the boundaries of a TAD found in all cells (Figure S1B). The *MYC* TAD encompasses a region previously described as a "gene desert," because this large span of DNA contains no other annotated protein-coding genes (Montavon and Duboule, 2012; Ovcharenko et al., 2005).

While all cells examined appear to share the TAD-spanning 2.8 Mb loop encompassing *MYC*, the loop structures within the neighborhood were found to be markedly different among the tumor types. The internal loops were dominated by interactions between a *MYC* promoter-proximal CTCF site and the diverse super-enhancers (Figures 1B and 1C). The major differences between these internal structures in the different tumor cells involved the different positions of the tumor-specific super-enhancer elements. Examination of Hi-C data for a broader spectrum of tumor cell types suggests that tumor cells generally have DNA contacts between the *MYC* promoter-proximal site and other sites within the 2.8 Mb *MYC* TAD (Figure S1B). This looping was not limited to cancer cells, because examination of enhancer and promoter-capture Hi-C data in a variety of normal cell types that express *MYC* (Javierre et al., 2016) revealed that cell-type-specific enhancers do indeed loop to the *MYC* proximal CTCF site (Figures S1C and S1D). This indicated that this CTCF site is also used during normal development by cell-type-specific enhancers to facilitate *MYC* expression and cellular proliferation.

Further examination of the *MYC* promoter-proximal region revealed three constitutive CTCF binding sites (Figure 1C). All three sites were found to be occupied by CTCF in a wide variety of normal cells and tumor cells, and this binding pattern is shared across species (Figure S1C). Previous studies have examined the role of CTCF binding at all three sites (Filippova et al., 1996; Gombert and Krumm, 2009; Gombert et al., 2003; Klenova et al., 1993; Rubio et al., 2008). The two sites located within the *MYC* gene have been shown to play roles in *MYC* transcript start site selection and in promoter-proximal pausing of RNA polymerase II (Filippova et al., 1996). The CTCF binding site located 2 kb upstream of the major transcription start site has been reported to protect the promoter from methylation and to be an insulator element (Gombert and Krumm, 2009; Gombert et al., 2003). The DNA interaction data described here, however, suggests that this upstream site dominates connections with distal enhancer elements, as the majority of reads in the DNA interaction data are associated with this site in all tumor cells examined (Figures 1C and S1E). The −2 kb CTCF binding site contains a number of putative CTCF binding motifs; one of these most closely matches the canonical CTCF motif in the JASPAR database (Sandelin et al., 2004) and occurs within a highly conserved sequence (Figure 1D). These features, the presence of CTCF sites in tumor super-enhancers, and the ability of two CTCF-bound sites to be brought together through CTCF homodimerization (Saldaña-Meyer et al., 2014; Yusufzai et al., 2004) led us to further study the possibility that the −2 kb site has an enhancer-docking function critical to *MYC* expression.

MYC Promoter Proximal CTCF Site Is Necessary for Enhancer-Promoter Looping and High *MYC* Expression

To determine whether the putative enhancer-docking site plays a functional role in *MYC* expression through DNA loop formation, small perturbations of the CTCF binding site were generated in both alleles of the tumor cell lines K562, HCT-116, Jurkat, and MCF7 using clustered regularly interspaced short palindromic repeats (CRISPR)/Cas9 (Figures 2A and 2B). Attempts at genetic perturbation by transfection with constructs carrying CRISPR/Cas9 with a guide RNA specifically targeting the CTCF motif upstream of the *MYC* gene did not yield viable clones. To allow cells to continue to proliferate if the CTCF motif deletion was lethal, cells were virally transduced with an exogenous *MYC* gene driven by a phosphoglycerate kinase (*PGK*) promoter (Figure S2A). This construct contained sequence differences in the 3′ UTR that allowed discrimination between the endogenous and exogenous *MYC* mRNAs. Cells expressing this exogenous *MYC* gene were then subjected to CRISPR/Cas9 perturbation. Clones were selected with small deletions or insertions disrupting the canonical CTCF motif (Figures 2B and S2B) and these cells were further characterized. CTCF chromatin immunoprecipitation quantative polymerase chain reaction (ChIP-qPCR) showed complete loss of CTCF binding to this site in K562 and HCT-116 cells and a 60%–70% reduction in CTCF binding at this site in Jurkat and MCF7 cells (Figure 2C). RNA analysis revealed a 70%–80% reduction of endogenous *MYC* mRNA in the absence of the enhancer-docking site in all of these cell types (Figure 2D). Furthermore, an inducible CRISPR/Cas9

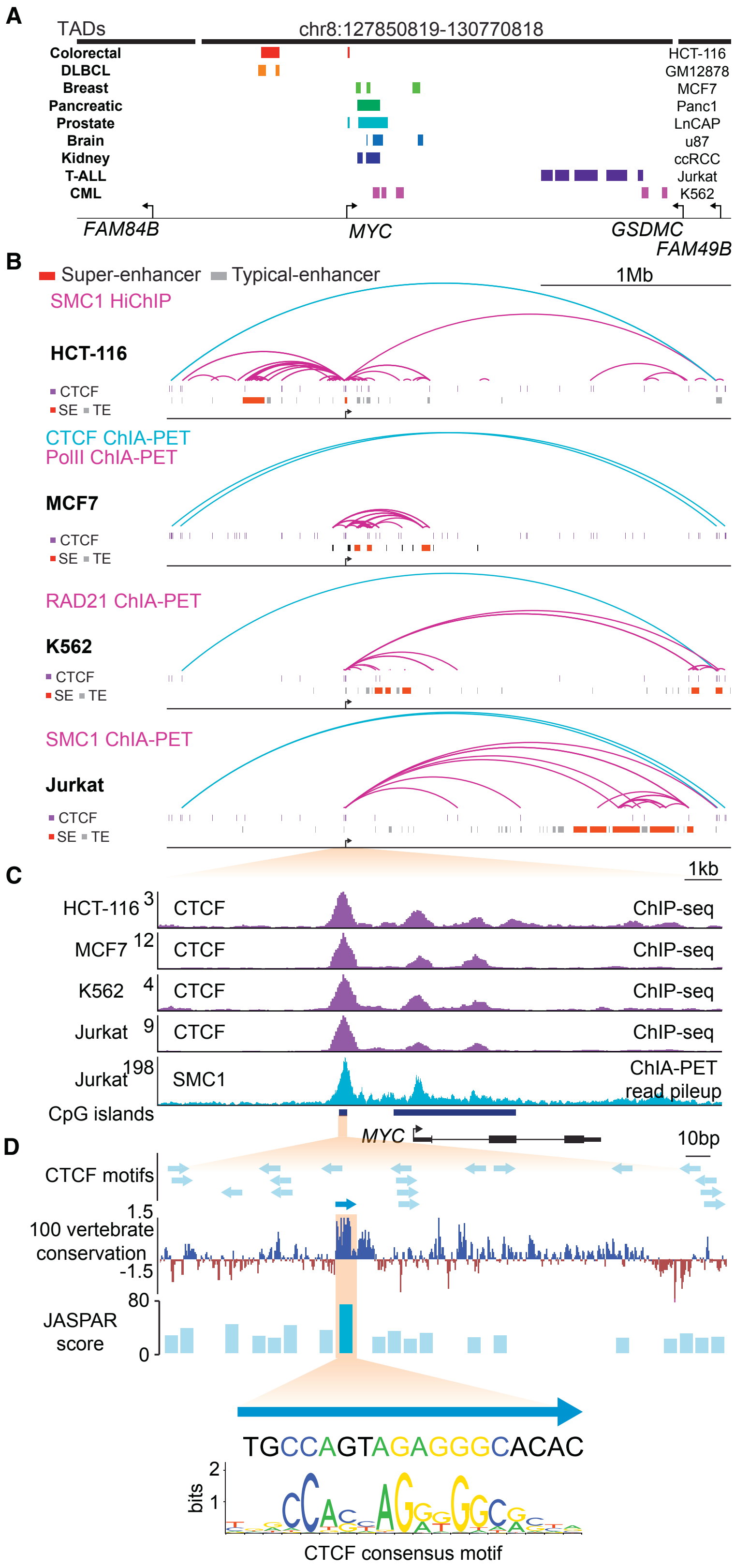

Figure 1. Cell-Type-Specific Super-Enhancers in the *MYC* Locus Loop to a Common CTCF Site

(A) The 4.5 Mb region surrounding the *MYC* gene. The 2.8 Mb TAD containing *MYC* and portions of the two adjacent TADs are indicated with thick black horizontal lines. Super-enhancers (data from Becket et al., 2016; Frietze et al., 2012; Lin et al., 2012; Pope et al., 2014; Wang et al., 2011) are shown in colored boxes for a panel of tumor cell lines that express *MYC*.

(B) Chromosome interaction data at the ~3 Mb *MYC* locus. For HCT-116, SMC1 HiChIP interactions with an origami score of at least 0.9 and a minimum PET count of 9 are shown as purple arcs; the insulated neighborhood spanning interaction, which encompasses the TAD, is shown as a blue arc (data from this study). For MCF7, Pol II ChIA-PET interactions with an origami score of at least 0.9 are shown as purple arcs; the insulated neighborhood spanning interactions from CTCF ChIA-PET data are shown in blue (data from ENCODE and Li et al., 2012). For K562, RAD21 ChIA-PET interactions with an origami score of at least 0.9 are shown as purple arcs; the insulated neighborhood spanning interaction is shown in blue and has an origami score of 0.44 (data from Heidari et al., 2014). For Jurkat, SMC1 ChIA-PET interactions with an origami score of at least 0.99 are shown as purple arcs; the insulated neighborhood spanning interactions are shown in blue (data from Hnisz et al., 2016a). CTCF ChIP-seq peaks are depicted as purple rectangles, super-enhancers are depicted as red rectangles, and typical enhancers are depicted as gray rectangles (data from this study; Hnisz et al., 2016a; Pope et al., 2014).

(C) CTCF ChIP-seq and SMC1 ChIA-PET read counts in the *MYC* promoter regions. Purple tracks display CTCF ChIP-seq signal in the four cell lines from (B). Light blue track displays the read counts from read pileup of Jurkat SMC1 ChIA-PET data, showing that the major peak of SMC1 ChIA-PET reads occurs at the −2 kb CTCF site. Dark blue bars indicate CpG islands. ChIP-seq read counts are shown in reads per million sequenced reads per base pair. ChIA-PET reads are shown as read counts per base pair.

(D) The top panel depicts all putative CTCF binding motifs as blue arrows, which indicate the orientation of the motif. The CTCF motif depicted in dark blue occurs in the most conserved region and shows the best match with consensus CTCF motif. 100 vertebrate conservation from the UCSC genome browser is depicted in the middle panel. The JASPAR score for all the motifs is indicated with blue bars. The position weight matrix for the canonical JASPAR CTCF motif and the actual sequence is shown below.

See also Figure S1.

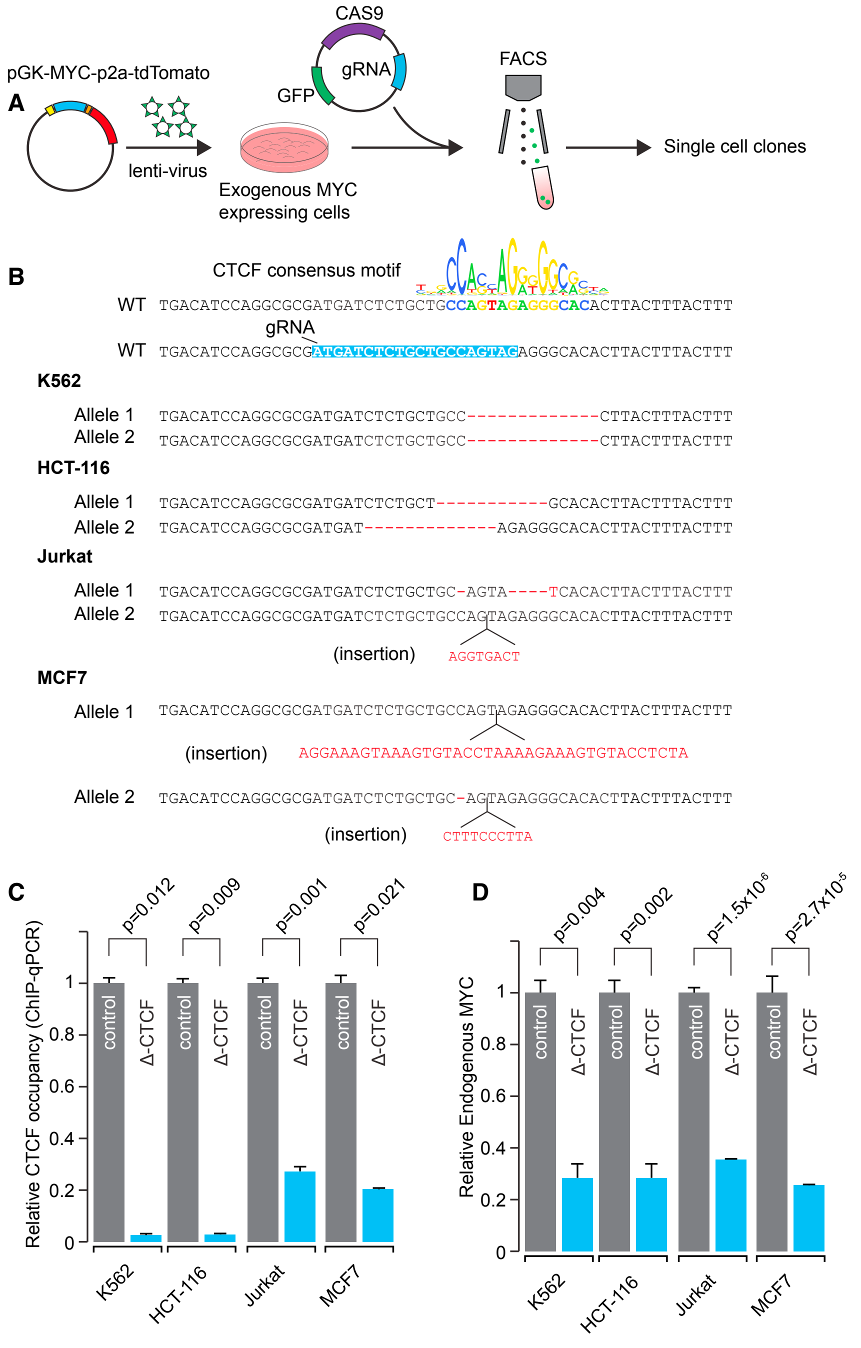

Figure 2. Perturbation of the Core CTCF Motif in the *MYC* CTCF Loop-Anchor Reduces CTCF Occupancy and *MYC* Expression

(A) Schematic representation of the experiment. HCT-116, K562, Jurkat, and MCF7 cells were transduced with a construct expressing *MYC* under a *PGK* promoter and selected for successful integration. These cells were then transiently transfected with plasmid carrying Cas9 and a gRNA targeting the CTCF binding motif. Positive cells were identified and selected using fluorescence-activated cell sorting (FACS). These cells were multiplied, and clonal populations were characterized.

(B) The DNA sequences in the vicinity of the core CTCF motif and the mutations generated in clonal populations of K562, HCT-116, Jurkat, and MCF7 cell lines. The reference (WT, wild-type) sequence highlighted in blue is complementary to the gRNA sequence targeting the most prominent CTCF motif (shown here in bold colored sequence). For the aneuploid MCF7 cell line, the two most common mutations are depicted.

(C) ChIP-qPCR showing reduction of the CTCF occupancy in Δ-CTCF K562, HCT-116, Jurkat, and MCF7 cells. p values were generated with a Student's t test. Error bars represent the SD of the mean from three technical replicates.

(D) qPCR showing reduction of endogenous *MYC* mRNA levels in Δ-CTCF K562, HCT-116, Jurkat, and MCF7 cells. p values were generated with a Student's t test. Error bars represent the SD of the mean from three biological replicates.

See also Figure S2.

perturbation model showed reduced proliferation for these four cell types upon induction of CTCF-site deletions (Figures S2C–S2G). These results indicate that the CTCF motif in the *MYC* enhancer-docking site is necessary for CTCF binding, for high levels of *MYC* expression and for cellular proliferation.

If the putative *MYC* enhancer-docking site contributes to looping interactions with distal enhancers, then the loss of this site should cause a decrease in DNA interactions between the *MYC* promoter and the distal super-enhancers. We used chromosome conformation capture combined with high-throughput sequencing (4C-seq) to compare super-enhancer interactions in K562 and HCT-116 cells with normal or perturbed CTCF binding motifs. The 4C-seq data in K562 cells indicated that the *MYC* enhancer-docking site interacts predominantly with distal super-enhancers located ~0.3 Mb and ~2 Mb downstream of the *MYC* gene and that the majority of these interactions were significantly reduced when the putative CTCF motif was perturbed (Figures 3A and S3A). In order to control for any direct effects of a genetic alteration near the viewpoint, 4C-seq was performed with a viewpoint placed in the downstream super-enhancer. This showed clear interactions with the *MYC* enhancer-docking site as well as with the nearby super-enhancer, and these interactions were significantly reduced upon perturbation of the CTCF motif (Figures 3B and S3B). Similar results were obtained in HCT-116 cells, where the viewpoint was centered on the super-enhancer located ~0.4 Mb upstream of the *MYC* gene (Figure S3C). These results showed that the CTCF site in the promoter-proximal region of *MYC* is important for optimal interaction with distal enhancers and supports the idea that this CTCF site functions as an enhancer-docking site.

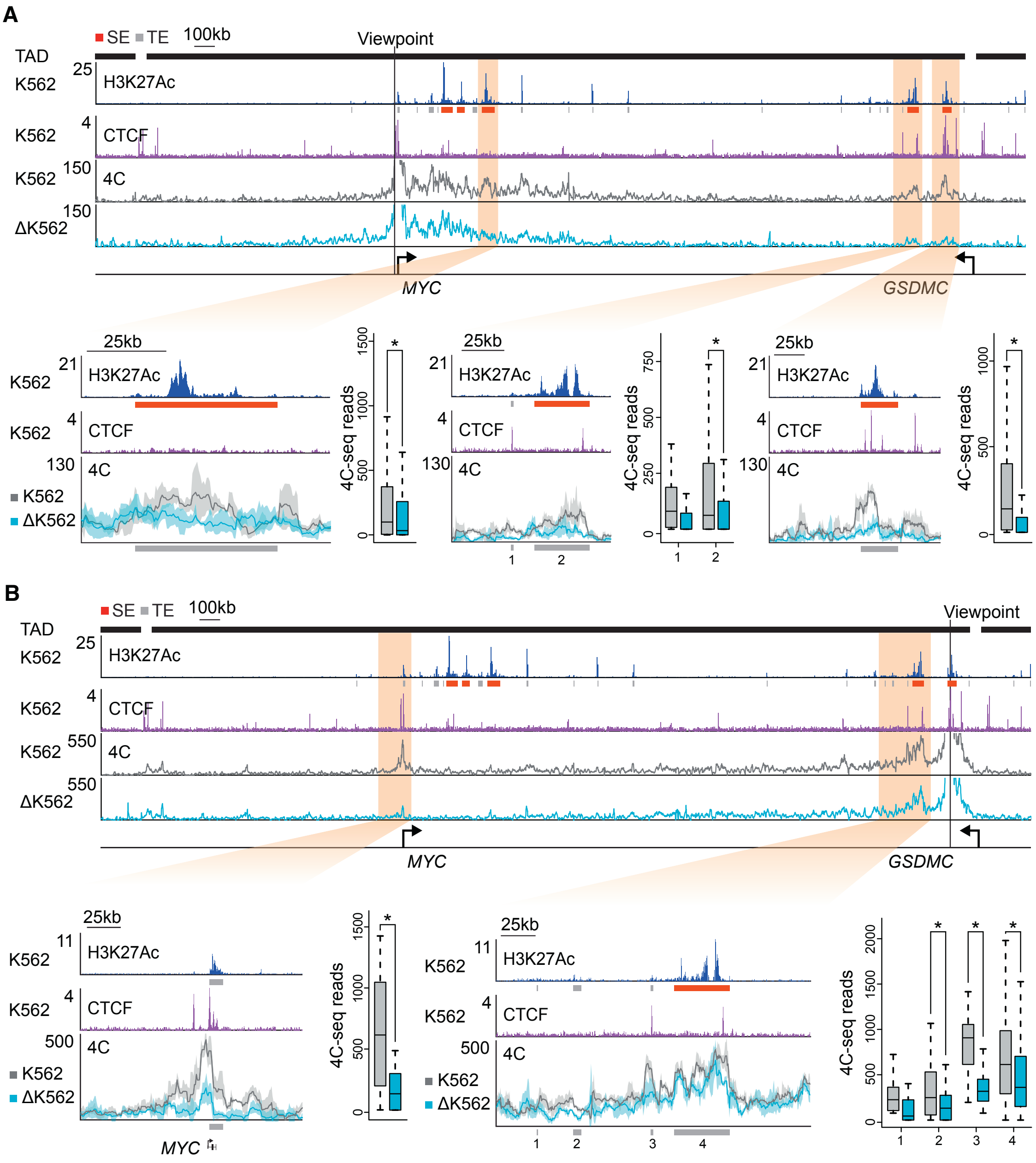

Figure 3. Perturbation of the *MYC* Enhancer-Docking Site Reduces Looping to Super-Enhancers

(A) 4C analysis showing reduced looping of the *MYC* promoter proximal CTCF site to super-enhancers in CTCF motif deletion cells (ΔK562) versus unmodified cells (K562). H3K27Ac ChIP-seq and CTCF ChIP-seq are shown in blue and purple colors respectively. Blowups show the 4C interactions for three K562 specific super-enhancers. The 4C viewpoint is situated 112 base pairs upstream of the deleted loop-anchor region.

(B) 4C analysis showing reduced looping of the *MYC* promoter proximal CTCF site to super-enhancers in CTCF motif deletion cells (ΔK562) versus unmodified cells (K562) using a viewpoint centered on the most distant super-enhancer downstream of the *MYC* gene. H3K27Ac ChIP-seq and CTCF ChIP-seq are shown in blue and purple, respectively. Blowups show the 4C interactions at the *MYC* promoter and distant super-enhancer near the viewpoint. Shading represents the 90% confidence interval based on three biological replicates. Peak calls from the H3K27Ac ChIP-seq were used to define the regions to be quantified and are indicated in gray boxes at the bottom of the panels. Boxplots show quantification of the reads per fragment for the indicated regions. p values were generated using Student's t test, and data pairs with a p value < 0.05 are indicated with an asterisk. Reads are shown in reads per million sequenced reads per base pair. Typical enhancers and super-enhancers are shown as gray boxes and red boxes, respectively.

See also Figure S3.

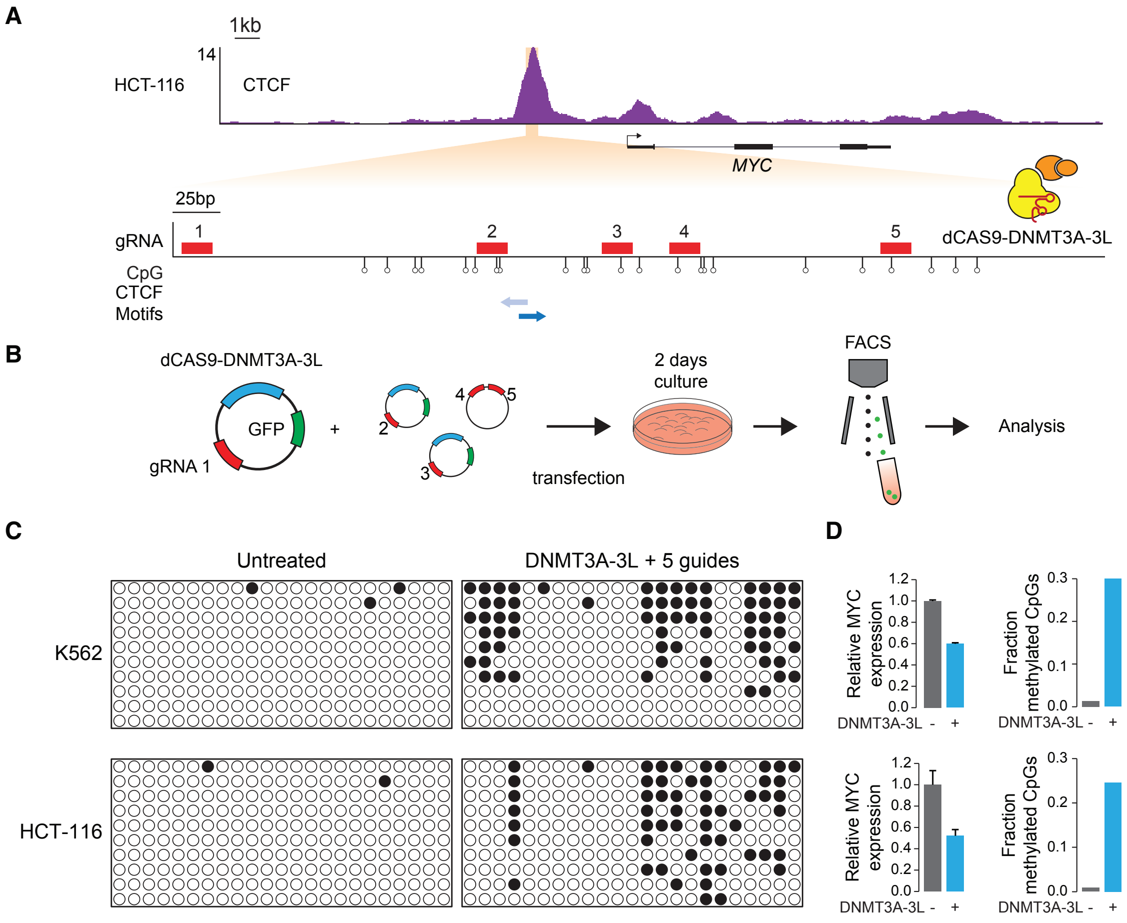

Figure 4. dCas9-Mediated Methylation of the CTCF Loop-Anchor Site Reduces *MYC* Expression in Tumor Cells

(A) Top panel shows CTCF ChIP-seq at the *MYC* gene region in HCT-116 cells. ChIP-seq reads are shown in reads per million sequenced reads per base pair. Bottom panels shows a blowup of the ~700-bp region underneath the CTCF peak depicting the CTCF motifs (blue arrows) and the gRNAs (red rectangles) used to target dCas9-DNMT3A-3L to the enhancer anchor. Lollipop symbols indicate the location of CpGs that are assayed for methylation levels in (C).

(B) Schematic representation of the experiment. HCT-116 or K562 cells were transfected with plasmids encoding the dCAS9-DNMT3A-3L, GFP, and a gRNA together with a plasmid encoding 2 additional gRNAs. HCT-116 or K562 cells were isolated by FACS after 2 days, and DNA and RNA were isolated.

(C) Methylation at *MYC* promoter loop-anchor site in untreated cells and cells transfected with dCas9-DNMT3A-3L in conjunction with the 5 indicated gRNAs.

(D) qPCR analysis of *MYC* mRNA levels and fraction of methylated CpGs for untreated and dCas9-DNMT3A-3L + 5 gRNA transfected cells. Error bars represent the SD of the mean for three biological replicates.

See also Figure S4.

Loss of *MYC* Expression with Methylation of Enhancer-Docking Site

CTCF binding is abrogated when its sequence motif is methylated (Bell and Felsenfeld, 2000; Maurano et al., 2015), and the *MYC* enhancer-docking site occurs within a CpG island that is consistently hypomethylated in different tumor types as well as in different normal tissues (Figures S4A and S4B). The recent development of tools that permit site-specific DNA methylation (Liu et al., 2016; Siddique et al., 2013) suggested a means to disrupt *MYC* expression by methylation of the enhancer-docking site. To achieve targeted methylation, we created a construct to express a dCas9 fusion protein consisting of the catalytic domain of DNMT3A and the interacting domain of DMNT3L. This dCas9-DNMT3A-3L protein was targeted to the *MYC* enhancer-docking site in K562 and HCT-116 cells using multiple guide RNAs that span the region (Figures 4A and 4B). The targeting of dCas9-DNMT3A-3L resulted in robust local DNA methylation (Figure 4C) and a 40%–50% reduction in mRNA levels in both cell types (Figure 4D). The methylated region likely contains binding sites for additional TFs that may be sensitive to DNA methylation, so it is possible that the reduced mRNA levels are due to multiple factors. In order to test whether disruption of TFs other than CTCF contribute to the reduction in *MYC* mRNA levels, the dCas9-DNMT3A-3L was targeted to the *MYC* enhancer-docking site in CTCF-site deleted K562 cells. No further reduction of *MYC* mRNA levels was observed under these conditions (Figures S4C and S4D), indicating that loss of CTCF was a major contributor to the observed reduction of *MYC* expression upon targeted methylation of the *MYC* enhancer-docking site. These results demonstrate that

epigenetic editing of the enhancer-docking site can reduce *MYC* expression.

CTCF Enhancer-Docking Sites at Additional Genes

Previous genomic studies have noted that CTCF might engender enhancer-promoter interactions at a minority of genes (Banani et al., 2017; Nora et al., 2017; Splinter et al., 2006; Zuin et al., 2014). We therefore identified the set of genes whose promoter-proximal regions contain CTCF-bound sites and that show evidence of enhancer interactions in K562, Jurkat, and HCT-116 cells. We identified all active transcription start sites (TSSs) that have at least one CTCF-bound site within 2.5 kb of the TSS that interacts with at least one enhancer. This yielded between 555 and 1,108 TSSs with a nearby CTCF site that loops to an active enhancer (Figure 5A; Table S2). We define these TSSs as having a putative CTCF enhancer-docking site. The majority of TSSs identified in this analysis were identified in only one cell type, with only 52 TSSs identified in all three cell types (Figure 5B). Nonetheless, these putative enhancer-docking sites tended to be constitutively bound by CTCF in all three cell types, and the CTCF motifs in these sites showed high sequence conservation (Figures 5C and 5D). This suggests that these putative enhancer-docking sites are occupied by CTCF regardless of interaction with active enhancers and that differences in cell-type-specific enhancers are largely responsible for differential use of enhancer-docking site genes in these cells.

Gene ontology analysis of the genes with putative enhancer-docking sites found different processes to be significantly enriched in each cell type, and these processes were dominated by the cellular identity of the cell lines (Figure S5A; Table S3). Common processes among the three cell types include cell cycle and other cancer-related processes such as gene expression and response to signaling (Figure S5A). A number of cancer-associated genes were found, including *TGIF1*, *VEGFA*, *RUNX1*, and *PIM1* (Figure 5E), as well as others (Figure S5B). We conclude that genes other than *MYC* are likely regulated by CTCF-bound enhancer-docking sites and that these include multiple cancer-associated genes.

DISCUSSION

Aberrant transcriptional activation of the *MYC* oncogene occurs frequently in tumor cells and is associated with tumor aggression. *MYC* resides within a 2.8 Mb TAD and its aberrant activation is generally accomplished by acquisition of a super-enhancer somewhere within that TAD. How these diverse cancer-specific super-enhancers loop long distances to specifically interact with *MYC* has not been clear. We find that the diverse super-enhancers commonly interact with, and depend on, a conserved CTCF binding site located 2 kb upstream of the *MYC* promoter. Because tumor super-enhancers can encompass genomic regions as large as 200 kb, and CTCF occupies sites that occur on average every 10 kb, there is considerable opportunity for super-enhancers to adventitiously contain a CTCF-bound site, which in turn could serve to interact with the *MYC* CTCF site (Table S6). Thus, different tumor super-enhancers have the opportunity to form through diverse mechanisms throughout this large TAD and can exploit the *MYC* CTCF site to interact with and activate *MYC* expression.

The concept that enhancer-promoter interactions generally occur within larger chromosomal loop structures such as TADs, which are themselves often formed by the interaction of CTCF proteins bound to each of the TAD loop anchors (Dekker and Mirny, 2016; Fraser et al., 2015; Gorkin et al., 2014a; Hnisz et al., 2016a), is supported by the observations described here. These larger loop structures tend to insulate enhancers and genes within the CTCF-CTCF loops from elements outside those loops. Constraining DNA interactions within CTCF-CTCF loop structures in this manner may facilitate proper enhancer-promoter contacts.

The evidence described here argues that diverse human tumor cell super-enhancers depend on the *MYC* CTCF site for optimal levels of enhancer-promoter looping and mRNA expression. A recent independent study in K562 cells used a tiling CRISPR screen to systematically perturb the *MYC* locus and also found that full *MYC* expression and cell proliferation is dependent on this region (Fulco et al., 2016). However, deletion of the −2 kb CTCF site has limited effects on *MYC* expression in mice (Dave et al., 2017; Gombert and Krumm, 2009), and some translocated enhancers can drive *MYC* expression in the absence of this CTCF site (Shiramizu et al., 1991). There are several potential explanations for these diverse results. It is possible that the −2 kb CTCF site is important for optimal *MYC* expression levels in human cells, but not in mice. It is conceivable that the deletion of a region containing the CTCF site can be compensated by features of the new enhancer landscape in the deletion mutations. Furthermore, additional mechanisms normally involved in enhancer-promoter interactions, such as YY1-YY1 interactions, may mask the loss of the CTCF site *in vivo*; YY1 is present in the *MYC* promoter region and is thus likely to contribute to DNA looping and expression (Weintraub et al., 2017).

Our studies suggest that an additional set of human genes, beyond *MYC*, may utilize promoter-proximal enhancer-docking sites to mediate cell-type-specific enhancer-promoter interactions. Such CTCF-mediated enhancer-promoter interactions are generally nested within larger CTCF-mediated loops that would function as insulated neighborhoods. At these genes with CTCF-mediated enhancer docking, the promoter-proximal enhancer-docking sites tend to be constitutively bound by CTCF and these binding sites tend to be highly conserved. Indeed, two studies have reported that these genes tend to lose expression upon perturbation of CTCF (Nora et al., 2017; Zuin et al., 2014), consistent with a role for CTCF in enhancer-promoter looping. Among these genes are cancer-associated genes that likely employ this mechanism to engender interactions with tumor-specific enhancers. For example, at *CSNK1A1*, a drug target in acute myeloid leukemia (AML) tumor cells (Järås et al., 2014), *VEGFA*, which is upregulated in many cancers (Goel and Mercurio, 2013), and *RUNX1*, a well-defined oncogene in AML (Deltcheva and Nimmo, 2017), the evidence suggests that super-enhancers in these cancer cells use a CTCF enhancer-docking mechanism to interact with the oncogene. Thus, a CTCF-dependent enhancer-docking mechanism, which presumably facilitates interaction with different

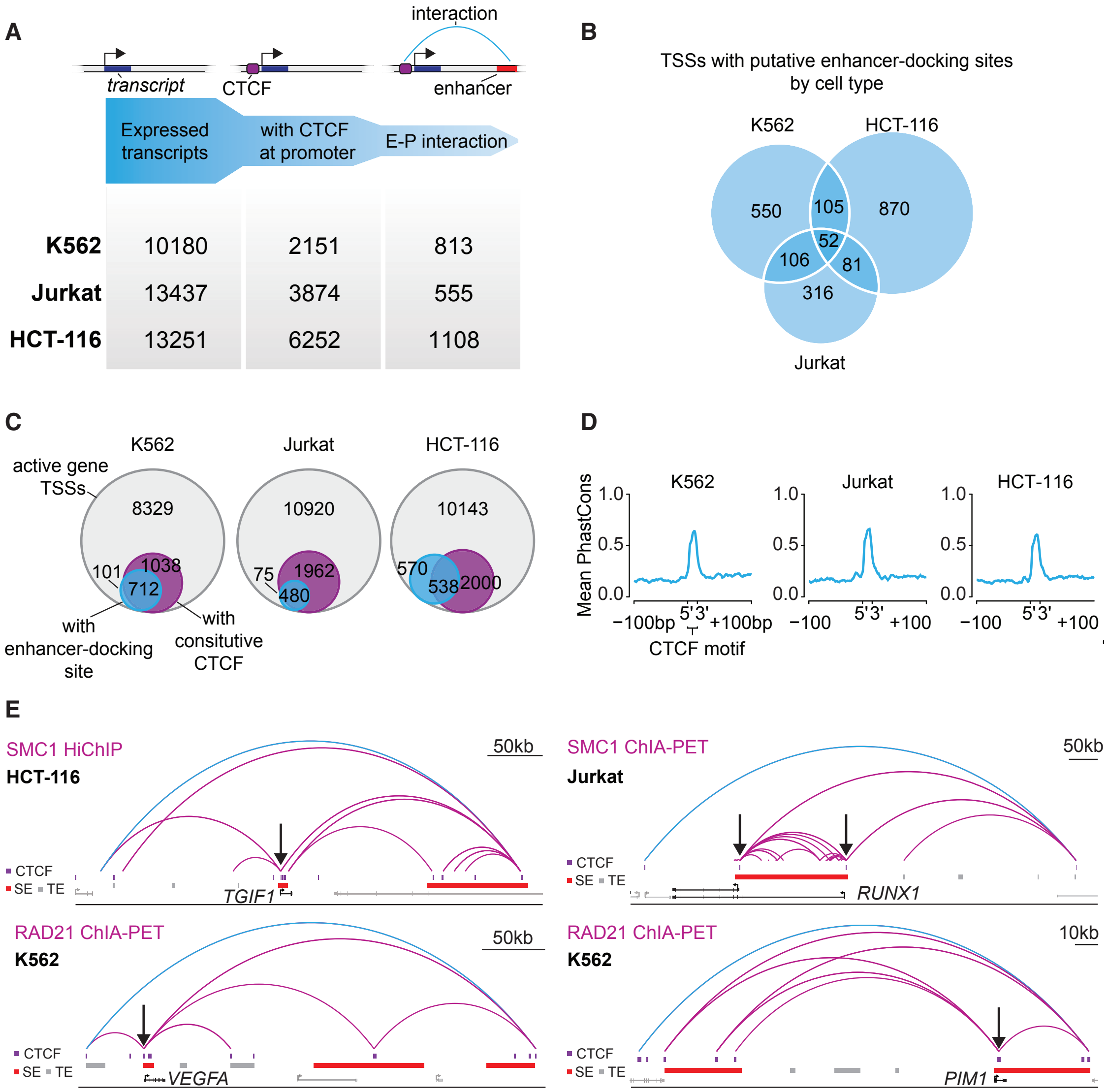

Figure 5. Putative Enhancer-Docking Sites Occur at Additional Genes with Prominent Roles in Cancer

(A) Identification of genes with putative enhancer-docking sites. Genes were filtered for their expression status, presence of a CTCF binding site within 2.5 kb of the TSS and evidence of looping to an active enhancer, defined by H3K27Ac ChIP-seq.

(B) Venn-diagram showing the overlap of TSSs with putative CTCF enhancer docking in K562, HCT-116, and Jurkat cells.

(C) Venn-diagrams showing the number of TSSs from active genes, the number of these that exhibit putative CTCF enhancer-docking and how many of these have a constitutive CTCF site within 2.5 kb of the TSS.

(D) Conservation analysis of the CTCF motifs in the CTCF-bound elements in putative enhancer-docking sites. The mean 46-way PhastCons score of the highest JASPAR scoring motifs in CTCF peaks within putative CTCF enhancer docking and their flanking regions are shown.

(E) Examples of genes with putative CTCF enhancer-docking sites from the different cell types analyzed. CTCF ChIP-seq peaks are shown as purple rectangles, typical enhancers are shown as gray rectangles, and super-enhancers are shown as red rectangles. Black arrows indicate the CTCF sites that may facilitate enhancer docking. The insulated neighborhood loop is shown in blue and loops internal to it are shown in purple. HCT-116 HiChIP interactions internal to the neighborhood with an origami score of at least 0.9 and a minimum PET count of 15 are shown for the *TGIF1* locus. Jurkat SMC1 ChIA-PET interactions internal to the neighborhood with an origami score of at least 0.97 are shown for the *RUNX1* locus. K562 RAD21 ChIA-PET interactions internal to the neighborhood with an origami score of at least 0.9 and a minimum PET count of 30 are shown for the *VEGFA* locus. K562 RAD21 ChIA-PET interactions internal to the neighborhood with an origami score of at least 0.9 and a minimum PET count of 30 are shown for the *PIM1* locus. Data are from this study and two others (Hnisz et al., 2016a; Heidari et al., 2014).

See also Figure S5.

cell-specific enhancers during development, is exploited by cancer cells to dysregulate expression of prominent oncogenes.

MYC dysregulation is a hallmark of cancer (Bradner et al., 2017). The c-MYC TF is an attractive target for cancer therapy because of the role that excessive c-MYC levels play in a broad spectrum of aggressive cancers (Felsher and Bishop, 1999; Jain et al., 2002; Soucek et al., 2008), but direct pharmacologic inhibition of c-MYC remains an elusive challenge in drug discovery (Bradner et al., 2017). The *MYC* enhancer-docking site, and presumably those of other oncogenes, can be repressed by dCas9-DNMT-mediated DNA methylation. Oncogene enhancer-docking sites may thus represent a vulnerability in multiple human cancers.

EXPERIMENTAL PROCEDURES

Further details and an outline for resources used in this work can be found in Supplemental Experimental Procedures.

CRISPR/Cas9 Genome Editing

Genome editing was performed using CRISPR/Cas9 essentially as described previously (Ran et al., 2013). The genomic sequences complementary to all guide RNAs are listed in Table S4.

ChIP-Seq

ChIP was performed as described previously (Lee et al., 2006). Approximately 30 million cells were crosslinked for 10 min at room temperature by the addition of one-tenth of the volume of 11% formaldehyde solution to the growth media followed by 5 min quenching with 125 mM glycine. Cells were washed twice with PBS, and then the supernatant was aspirated and the cell pellet was flash frozen at −80°C. 100 μL Protein G Dynabeads (Thermo 10003D) were blocked with 0.5% BSA (w/v) in PBS. Magnetic beads were bound with 40 μL anti-CTCF antibody (Millipore 07-729). Nuclei were isolated as previously described (Lee et al., 2006) and sonicated in lysis buffer on a Misonix 3000 sonicator for 5 cycles at 30 s each on ice (18–21 W) with 60 s on ice between cycles. Sonicated lysates were cleared once by centrifugation and incubated overnight at 4°C with magnetic beads bound with antibody to enrich for DNA fragments bound by the indicated factor. Beads were washed with wash buffers A, B, C, and D sequentially. DNA was eluted, cross-links were reversed, and DNA was purified with phenol chloroform extraction and ethanol precipitation. Libraries for Illumina sequencing were prepared following the Illumina TruSeq DNA Sample Preparation v2 kit and sequenced on the Illumina HiSeq 2500 for 40 bases in single-read mode.

4C-Seq

A modified version of 4C-seq (van de Werken et al., 2012) was developed (Supplemental Experimental Procedures). The major change was the ligation is performed in intact nuclei (*in situ*). This change was incorporated because previous work has noted that *in situ* ligation dramatically decreases the rate of chimeric ligations and background interactions (Rao et al., 2014).

HiChIP

HiChIP was performed essentially as described (Mumbach et al., 2016). 10 million HCT116 cells were crosslinked for 10 min at room temperature by the addition of one-tenth of the volume of 11% formaldehyde solution to the growth media followed by 5-min quenching with glycine. Cells were washed twice with PBS, and then the cell pellet was flash frozen in liquid nitrogen. Frozen samples were processed according to protocol (Supplemental Experimental Procedures).

Targeted Methylation and Bisulfite Sequencing

To perform targeted methylation, cells were transfected with a dCas9-DNMT3A-3L construct and five guides. To generate the dCas9-DNMT3A-3L construct, dCas9 was isolated from pSQL1658 (Addgene 51023) by PCR. Cas9 was removed from pX330-Cas9 (Addgene 42230) and replaced by DNMT3A-3L (Siddique et al., 2013). Guide RNAs used for targeting can be found in Table S4.

Statistical Methods

ChIP-Seq Data Analysis

ChIP-seq datasets were generated for this study as well as collated from previous studies (Table S5) and were aligned using Bowtie (version 0.12.2) to the human genome (build hg19, GRCh37) with parameter -k 1 -m 1 -n 2. We used MACS version 1.4.2 with the parameter “-no-model-keep-dup=auto.” A p value threshold of enrichment of 1e-09 was used.

4C Analysis

4C-seq reads were trimmed and mapped using bowtie with options -k 1 -m 1 against the hg19 genome assembly. We only used the reads from non-blind fragments for further analysis. The normalized profile of each sample was smoothened using a 6-kb running mean at 500-bp steps across the genome. Quantification of the 4C signal counted the reads per fragment per million sequenced reads in the super-enhancers or the CTCF MACS peak calls.

HiChIP and ChIA-PET Data Analysis

We developed a new software pipeline and analytical method called origami to process HiChIP and chromatin interaction analysis by paired-end tag sequencing (ChIA-PET). The software and releases can be found at https://github.com/younglab/origami using version alpha20160828. The ChIA-PET datasets analyzed along with their corresponding linker sequence and called interactions in and around the *MYC* TAD can be found in Table S4. Each ChIA-PET datasets was processed as follows: the reads were first trimmed and aligned using origami-alignment. Each end of a paired end tag (PET) with a linker sequence were separately mapped to the hg19 genome assembly using bowtie with the following options: -v 1 -k 1 -m 1. After alignment, the separated PETs were re-paired in the final BAM output. After repairing, all duplicated PETs within the data were removed. Peaks were called on the re-paired ChIA-PET reads using MACS1 v1.4.2 with the following parameters: -nolambda -nomodel -p 1e−9.

DATA AND SOFTWARE AVAILABILITY

The accession number for the sequencing data reported in this paper is GEO: GSE92881. The origami algorithm is accessible at https://github.com/younglab/origami (version alpha20160828).

SUPPLEMENTAL INFORMATION

Supplemental Information includes Supplemental Experimental Procedures, five figures, and six tables and can be found with this article online at https://doi.org/10.1016/j.celrep.2018.03.056.

ACKNOWLEDGMENTS

We thank X. Shawn Liu and Rudolf Jaenisch for their help with the targeted methylation of the CTCF-bound element. We thank the Whitehead Institute Genome Technology Core and the FACS facility for their contribution to this work. This work is supported by NIH grant HG002668 (R.A.Y.), a Rubicon fellowship by NWO (J.S.), Ludwig Graduate Fellowship funds (A.S.W.), American Cancer Society fellowship PF-16-146-01-DMC (D.S.D.), a Margaret and Herman Sokol Postdoctoral Award (D.H.), and National Science Foundation GRFP funds (A.V.Z).

AUTHOR CONTRIBUTIONS

J.S., J.C.M., and R.A.Y. wrote the manuscript. J.S. and J.C.M. performed methylation, perturbation, and other experiments. D.H. performed HiChIP. D.S.D. analyzed HiChIP and ChIA-PET data and wrote origami. J.S., J.C.M., and A.V.Z. performed 4C experiments. A.S.W. made inducible CTCF-site deletion lines. T.I.L. provided helpful feedback and R.A.Y. supervised.

DECLARATION OF INTERESTS

The Whitehead Institute filed a patent application based on this study. R.A.Y. is a founder of Syros Pharmaceuticals, of Marauder Therapeutics and of Omega Therapeutics. The remaining authors declare no competing interests.

Received: December 8, 2017
Revised: February 9, 2018
Accepted: March 14, 2018
Published: April 10, 2018

REFERENCES

Banani, S.F., Lee, H.O., Hyman, A.A., and Rosen, M.K. (2017). Biomolecular condensates: organizers of cellular biochemistry. Nat. Rev. Mol. Cell Biol. *18*, 285–298.

Becket, E., Chopra, S., Duymich, C.E., Lin, J.J., You, J.S., Pandiyan, K., Nichols, P.W., Siegmund, K.D., Charlet, J., Weisenberger, D.J., et al. (2016). Identification of DNA methylation-independent epigenetic events underlying clear cell renal cell carcinoma. Cancer Res. *76*, 1954–1964.

Bell, A.C., and Felsenfeld, G. (2000). Methylation of a CTCF-dependent boundary controls imprinted expression of the Igf2 gene. Nature *405*, 482–485.

Berns, E.M.J.J., Klijn, J.G.M., van Putten, W.L., van Staveren, I.L., Portengen, H., and Foekens, J.A. (1992). c-myc amplification is a better prognostic factor than HER2/neu amplification in primary breast cancer. Cancer Res. *52*, 1107–1113.

Bonev, B., and Cavalli, G. (2016). Organization and function of the 3D genome. Nat. Rev. Genet. *17*, 661–678.

Bradner, J.E., Hnisz, D., and Young, R.A. (2017). Transcriptional addiction in cancer. Cell *168*, 629–643.

Buecker, C., and Wysocka, J. (2012). Enhancers as information integration hubs in development: lessons from genomics. Trends Genet. *28*, 276–284.

Bulger, M., and Groudine, M. (2011). Functional and mechanistic diversity of distal transcription enhancers. Cell *144*, 327–339.

Chapuy, B., McKeown, M.R., Lin, C.Y., Monti, S., Roemer, M.G.M., Qi, J., Rahl, P.B., Sun, H.H., Yeda, K.T., Doench, J.G., et al. (2013). Discovery and characterization of super-enhancer-associated dependencies in diffuse large B cell lymphoma. Cancer Cell *24*, 777–790.

Cuddapah, S., Jothi, R., Schones, D.E., Roh, T.Y., Cui, K., and Zhao, K. (2009). Global analysis of the insulator binding protein CTCF in chromatin barrier regions reveals demarcation of active and repressive domains. Genome Res. *19*, 24–32.

Dang, C.V. (2012). MYC on the path to cancer. Cell *149*, 22–35.

Dave, K., Sur, I., Yan, J., Zhang, J., Kaasinen, E., Zhong, F., Blaas, L., Li, X., Kharazi, S., Gustafsson, C., et al. (2017). Mice deficient of*Myc*super-enhancer region reveal differential control mechanism between normal and pathological growth. eLife *6*, 1–25.

de Wit, E., Bouwman, B.A., Zhu, Y., Klous, P., Splinter, E., Verstegen, M.J., Krijger, P.H., Festuccia, N., Nora, E.P., Welling, M., et al. (2013). The pluripotent genome in three dimensions is shaped around pluripotency factors. Nature *501*, 227–231.

Dekker, J., and Mirny, L. (2016). The 3D genome as moderator of chromosomal communication. Cell *164*, 1110–1121.

Deltcheva, E., and Nimmo, R. (2017). RUNX transcription factors at the interface of stem cells and cancer. Biochem. J. *474*, 1755–1768.

Dixon, J.R., Selvaraj, S., Yue, F., Kim, A., Li, Y., Shen, Y., Hu, M., Liu, J.S., and Ren, B. (2012). Topological domains in mammalian genomes identified by analysis of chromatin interactions. Nature *485*, 376–380.

Dowen, J.M., Fan, Z.P., Hnisz, D., Ren, G., Abraham, B.J., Zhang, L.N., Weintraub, A.S., Schujiers, J., Lee, T.I., Zhao, K., and Young, R.A. (2014). Control of cell identity genes occurs in insulated neighborhoods in mammalian chromosomes. Cell *159*, 374–387.

Felsher, D.W., and Bishop, J.M. (1999). Reversible tumorigenesis by MYC in hematopoietic lineages. Mol. Cell *4*, 199–207.

Filippova, G.N., Fagerlie, S., Klenova, E.M., Myers, C., Dehner, Y., Goodwin, G., Neiman, P.E., Collins, S.J., and Lobanenkov, V.V. (1996). An exceptionally conserved transcriptional repressor, CTCF, employs different combinations of zinc fingers to bind diverged promoter sequences of avian and mammalian c-myc oncogenes. Mol. Cell. Biol. *16*, 2802–2813.

Franke, M., Ibrahim, D.M., Andrey, G., Schwarzer, W., Heinrich, V., Schöpflin, R., Kraft, K., Kempfer, R., Jerković, I., Chan, W.-L., et al. (2016). Formation of new chromatin domains determines pathogenicity of genomic duplications. Nature *538*, 265–269.

Fraser, J., Ferrai, C., Chiariello, A.M., Schueler, M., Rito, T., Laudanno, G., Barbieri, M., Moore, B.L., Kraemer, D.C., Aitken, S., et al.; FANTOM Consortium (2015). Hierarchical folding and reorganization of chromosomes are linked to transcriptional changes in cellular differentiation. Mol. Syst. Biol. *11*, 852.

Frietze, S., Wang, R., Yao, L., Tak, Y.G., Ye, Z., Gaddis, M., Witt, H., Farnham, P.J., and Jin, V.X. (2012). Cell type-specific binding patterns reveal that TCF7L2 can be tethered to the genome by association with GATA3. Genome Biol. *13*, R52.

Fulco, C.P., Munschauer, M., Anyoha, R., Munson, G., Grossman, S.R., Perez, E.M., Kane, M., Cleary, B., Lander, E.S., and Engreitz, J.M. (2016). Systematic mapping of functional enhancer-promoter connections with CRISPR interference. Science *354*, 769–773.

Gabay, M., Li, Y., and Felsher, D.W. (2014). MYC activation is a hallmark of cancer initiation and maintenance. Cold Spring Harb. Perspect. Med. *4*, 1–14.

Gibcus, J.H., and Dekker, J. (2013). The hierarchy of the 3D genome. Mol. Cell *49*, 773–782.

Goel, H.L., and Mercurio, A.M. (2013). VEGF targets the tumour cell. Nat. Rev. Cancer *13*, 871–882.

Gombert, W.M., and Krumm, A. (2009). Targeted deletion of multiple CTCF-binding elements in the human C-MYC gene reveals a requirement for CTCF in C-MYC expression. PLoS ONE *4*, e6109.

Gombert, W.M., Farris, S.D., Rubio, E.D., Morey-Rosler, K.M., Schubach, W.H., and Krumm, A. (2003). The c-myc insulator element and matrix attachment regions define the c-myc chromosomal domain. Mol. Cell. Biol. *23*, 9338–9348.

Gorkin, D.U., Leung, D., and Ren, B. (2014a). The 3D genome in transcriptional regulation and pluripotency. Cell Stem Cell *14*, 762–775.

Gröschel, S., Sanders, M.A., Hoogenboezem, R., de Wit, E., Bouwman, B.A.M., Erpelinck, C., van der Velden, V.H.J., Havermans, M., Avellino, R., van Lom, K., et al. (2014). A single oncogenic enhancer rearrangement causes concomitant EVI1 and GATA2 deregulation in leukemia. Cell *157*, 369–381.

Grotzer, M.A., Hogarty, M.D., Janss, A.J., Liu, X., Zhao, H., Eggert, A., Sutton, L.N., Rorke, L.B., Brodeur, G.M., and Phillips, P.C. (2001). MYC messenger RNA expression predicts survival outcome in childhood primitive neuroectodermal tumor/medulloblastoma. Clin. Cancer Res. *7*, 2425–2433.

Handoko, L., Xu, H., Li, G., Ngan, C.Y., Chew, E., Schnapp, M., Lee, C.W.H., Ye, C., Ping, J.L.H., Mulawadi, F., et al. (2011). CTCF-mediated functional chromatin interactome in pluripotent cells. Nat. Genet. *43*, 630–638.

Heidari, N., Phanstiel, D.H., He, C., Grubert, F., Jahanbani, F., Kasowski, M., Zhang, M.Q., and Snyder, M.P. (2014). Genome-wide map of regulatory interactions in the human genome. Genome Res. *24*, 1905–1917.

Herranz, D., Ambesi-Impiombato, A., Palomero, T., Schnell, S.A., Belver, L., Wendorff, A.A., Xu, L., Castillo-Martin, M., Llobet-Navás, D., Cordon-Cardo, C., et al. (2014). A NOTCH1-driven MYC enhancer promotes T cell development, transformation and acute lymphoblastic leukemia. Nat. Med. *20*, 1130–1137.

Hnisz, D., Abraham, B.J., Lee, T.I., Lau, A., Saint-André, V., Sigova, A.A., Hoke, H.A., and Young, R.A. (2013). Super-enhancers in the control of cell identity and disease. Cell *155*, 934–947.

Hnisz, D., Weintraub, A.S., Day, D.S., Valton, A.-L., Bak, R.O., Li, C.H., Goldmann, J., Lajoie, B.R., Fan, Z.P., Sigova, A.A., et al. (2016a). Activation of

proto-oncogenes by disruption of chromosome neighborhoods. Science *351*, 1454–1458.

Hnisz, D., Day, D.S., and Young, R.A. (2016b). Insulated Neighborhoods: structural and functional units of mammalian gene control. Cell *167*, 1188–1200.

Hnisz, D., Schuijers, J., Li, C.H., and Young, R.A. (2018). Regulation and dysregulation of chromosome structure in cancer. Annu. Rev. Cancer Biol. *2*, 21–40.

Jain, M., Arvanitis, C., Chu, K., Dewey, W., Leonhardt, E., Trinh, M., Sundberg, C.D., Bishop, J.M., and Felsher, D.W. (2002). Sustained loss of a neoplastic phenotype by brief inactivation of MYC. Science *297*, 102–104.

Järås, M., Miller, P.G., Chu, L.P., Puram, R.V., Fink, E.C., Schneider, R.K., Al-Shahrour, F., Peña, P., Breyfogle, L.J., Hartwell, K.A., et al. (2014). Csnk1a1 inhibition has p53-dependent therapeutic efficacy in acute myeloid leukemia. J. Exp. Med. *211*, 605–612.

Javierre, B.M., Burren, O.S., Wilder, S.P., Kreuzhuber, R., Hill, S.M., Sewitz, S., Cairns, J., Wingett, S.W., Várnai, C., Thiecke, M.J., et al.; BLUEPRINT Consortium (2016). Lineage-specific genome architecture links enhancers and non-coding disease variants to target gene promoters. Cell *167*, 1369–1384.

Ji, X., Dadon, D.B., Powell, B.E., Fan, Z.P., Borges-Rivera, D., Shachar, S., Weintraub, A.S., Hnisz, D., Pegoraro, G., Lee, T.I., et al. (2016). 3D chromosome regulatory landscape of human pluripotent cells. Cell Stem Cell *18*, 262–275.

Kim, T.H., Abdullaev, Z.K., Smith, A.D., Ching, K.A., Loukinov, D.I., Green, R.D.D., Zhang, M.Q., Lobanenkov, V.V., and Ren, B. (2007). Analysis of the vertebrate insulator protein CTCF-binding sites in the human genome. Cell *128*, 1231–1245.

Klenova, E.M., Nicolas, R.H., Paterson, H.F., Carne, A.F., Heath, C.M., Goodwin, G.H., Neiman, P.E., and Lobanenkov, V.V. (1993). CTCF, a conserved nuclear factor required for optimal transcriptional activity of the chicken c-myc gene, is an 11-Zn-finger protein differentially expressed in multiple forms. Mol. Cell. Biol. *13*, 7612–7624.

Lee, T.I., Johnstone, S.E., and Young, R.A. (2006). Chromatin immunoprecipitation and microarray-based analysis of protein location. Nat. Protoc. *1*, 729–748.

Li, G., Ruan, X., Auerbach, R.K., Sandhu, K.S., Zheng, M., Wang, P., Poh, H.M., Goh, Y., Lim, J., Zhang, J., et al. (2012). Extensive promoter-centered chromatin interactions provide a topological basis for transcription regulation. Cell *148*, 84–98.

Lin, C.Y., Lovén, J., Rahl, P.B., Paranal, R.M., Burge, C.B., Bradner, J.E., Lee, T.I., and Young, R.A. (2012). Transcriptional amplification in tumor cells with elevated c-Myc. Cell *151*, 56–67.

Lin, C.Y., Erkek, S., Tong, Y., Yin, L., Federation, A.J., Zapatka, M., Haldipur, P., Kawauchi, D., Risch, T., Warnatz, H.-J., et al. (2016). Active medulloblastoma enhancers reveal subgroup-specific cellular origins. Nature *530*, 57–62.

Liu, M., Maurano, M.T., Wang, H., Qi, H., Song, C.-Z., Navas, P.A., Emery, D.W., Stamatoyannopoulos, J.A., and Stamatoyannopoulos, G. (2015). Genomic discovery of potent chromatin insulators for human gene therapy. Nat. Biotechnol. *33*, 198–203.

Liu, X.S., Wu, H., Ji, X., Stelzer, Y., Wu, X., Czauderna, S., Shu, J., Dadon, D., Young, R.A., and Jaenisch, R. (2016). Editing DNA methylation in the mammalian genome. Cell *167*, 233–247.e17.

Lovén, J., Hoke, H.A., Lin, C.Y., Lau, A., Orlando, D.A., Vakoc, C.R., Bradner, J.E., Lee, T.I., and Young, R.A. (2013). Selective inhibition of tumor oncogenes by disruption of super-enhancers. Cell *153*, 320–334.

Maurano, M.T., Wang, H., John, S., Shafer, A., Canfield, T., Lee, K., and Stamatoyannopoulos, J.A. (2015). Role of DNA methylation in modulating transcription factor occupancy. Cell Rep. *12*, 1184–1195.

Montavon, T., and Duboule, D. (2012). Landscapes and archipelagos: spatial organization of gene regulation in vertebrates. Trends Cell Biol. *22*, 347–354.

Müeller-Storm, H.P., Sogo, J.M., and Schaffner, W. (1989). An enhancer stimulates transcription in trans when attached to the promoter via a protein bridge. Cell *58*, 767–777.

Muerdter, F., and Stark, A. (2016). Gene regulation: activation through space. Curr. Biol. *26*, R895–R898.

Mumbach, M.R., Rubin, A.J., Flynn, R.A., Dai, C., Khavari, P.A., Greenleaf, W.J., and Chang, H.Y. (2016). HiChIP: efficient and sensitive analysis of protein-directed genome architecture. Nat. Methods *13*, 919–922.

Narendra, V., Rocha, P.P., An, D., Raviram, R., Skok, J.A., Mazzoni, E.O., and Reinberg, D. (2015). CTCF establishes discrete functional chromatin domains at the Hox clusters during differentiation. Science *347*, 1017–1021.

Nora, E.P., Lajoie, B.R., Schulz, E.G., Giorgetti, L., Okamoto, I., Servant, N., Piolot, T., van Berkum, N.L., Meisig, J., Sedat, J., et al. (2012). Spatial partitioning of the regulatory landscape of the X-inactivation centre. Nature *485*, 381–385.

Nora, E.P., Goloborodko, A., Valton, A.-L., Gibcus, J.H., Uebersohn, A., Abdennur, N., Dekker, J., Mirny, L.A., and Bruneau, B.G. (2017). Targeted degradation of CTCF decouples local insulation of chromosome domains from genomic compartmentalization. Cell *169*, 930–944.

Ovcharenko, I., Loots, G.G., Nobrega, M.A., Hardison, R.C., Miller, W., and Stubbs, L. (2005). Evolution and functional classification of vertebrate gene deserts. Genome Res. *15*, 137–145.

Parker, S.C.J., Stitzel, M.L., Taylor, D.L., Orozco, J.M., Erdos, M.R., Akiyama, J.A., van Bueren, K.L., Chines, P.S., Narisu, N., Black, B.L., et al.; NISC Comparative Sequencing Program; National Institutes of Health Intramural Sequencing Center Comparative Sequencing Program Authors; NISC Comparative Sequencing Program Authors (2013). Chromatin stretch enhancer states drive cell-specific gene regulation and harbor human disease risk variants. Proc. Natl. Acad. Sci. USA *110*, 17921–17926.

Phillips-Cremins, J.E., Sauria, M.E., Sanyal, A., Gerasimova, T.I., Lajoie, B.R., Bell, J.S., Ong, C.-T.T., Hookway, T.A., Guo, C., Sun, Y., et al. (2013). Architectural protein subclasses shape 3D organization of genomes during lineage commitment. Cell *153*, 1281–1295.

Pope, B.D., Ryba, T., Dileep, V., Yue, F., Wu, W., Denas, O., Vera, D.L., Wang, Y., Hansen, R.S., Canfield, T.K., et al. (2014). Topologically associating domains are stable units of replication-timing regulation. Nature *515*, 402–405.

Ran, F.A., Hsu, P.D., Wright, J., Agarwala, V., Scott, D.A., and Zhang, F. (2013). Genome engineering using the CRISPR-Cas9 system. Nat. Protoc. *8*, 2281–2308.

Rao, S.S.P., Huntley, M.H., Durand, N.C., Stamenova, E.K., Bochkov, I.D., Robinson, J.T., Sanborn, A.L., Machol, I., Omer, A.D., Lander, E.S., and Aiden, E.L. (2014). A 3D map of the human genome at kilobase resolution reveals principles of chromatin looping. Cell *159*, 1665–1680.

Rubio, E.D., Reiss, D.J., Welcsh, P.L., Disteche, C.M., Filippova, G.N., Baliga, N.S., Aebersold, R., Ranish, J.A., and Krumm, A. (2008). CTCF physically links cohesin to chromatin. Proc. Natl. Acad. Sci. USA *105*, 8309–8314.

Saldaña-Meyer, R., González-Buendía, E., Guerrero, G., Narendra, V., Bonasio, R., Recillas-Targa, F., and Reinberg, D. (2014). CTCF regulates the human p53 gene through direct interaction with its natural antisense transcript, Wrap53. Genes Dev. *28*, 723–734.

Sandelin, A., Alkema, W., Engström, P., Wasserman, W.W., and Lenhard, B. (2004). JASPAR: an open-access database for eukaryotic transcription factor binding profiles. Nucleic Acids Res. *32*, D91–D94.

Shi, J., Whyte, W.A., Zepeda-Mendoza, C.J., Milazzo, J.P., Shen, C., Roe, J.S., Minder, J.L., Mercan, F., Wang, E., Eckersley-Maslin, M.A., et al. (2013). Role of SWI/SNF in acute leukemia maintenance and enhancer-mediated Myc regulation. Genes Dev. *27*, 2648–2662.

Shiramizu, B., Barriga, F., Neequaye, J., Jafri, A., Dalla-Favera, R., Neri, A., Guttierez, M., Levine, P., and Magrath, I. (1991). Patterns of chromosomal breakpoint locations in Burkitt's lymphoma: relevance to geography and Epstein-Barr virus association. Blood *77*, 1516–1526.

Siddique, A.N., Nunna, S., Rajavelu, A., Zhang, Y., Jurkowska, R.Z., Reinhardt, R., Rots, M.G., Ragozin, S., Jurkowski, T.P., and Jeltsch, A. (2013). Targeted methylation and gene silencing of VEGF-A in human cells by using a designed Dnmt3a-Dnmt3L single-chain fusion protein with increased DNA methylation activity. J. Mol. Biol. *425*, 479–491.

Soucek, L., Whitfield, J., Martins, C.P., Finch, A.J., Murphy, D.J., Sodir, N.M., Karnezis, A.N., Swigart, L.B., Nasi, S., and Evan, G.I. (2008). Modelling Myc inhibition as a cancer therapy. Nature *455*, 679–683.

Spitz, F. (2016). Gene regulation at a distance: From remote enhancers to 3D regulatory ensembles. Semin. Cell Dev. Biol. *57*, 57–67.

Splinter, E., Heath, H., Kooren, J., Palstra, R.-J., Klous, P., Grosveld, F., Galjart, N., and de Laat, W. (2006). CTCF mediates long-range chromatin looping and local histone modification in the beta-globin locus. Genes Dev. *20*, 2349–2354.

Tang, Z., Luo, O.J., Li, X., Zheng, M., Zhu, J.J., Szalaj, P., Trzaskoma, P., Magalska, A., Wlodarczyk, J., Ruszczycki, B., et al. (2015). CTCF-mediated human 3D genome architecture reveals chromatin topology for transcription. Cell *163*, 1611–1627.

van de Werken, H.J.G., Landan, G., Holwerda, S.J.B., Hoichman, M., Klous, P., Chachik, R., Splinter, E., Valdes-Quezada, C., Öz, Y., Bouwman, B.A.M., et al. (2012). Robust 4C-seq data analysis to screen for regulatory DNA interactions. Nat. Methods *9*, 969–972.

Wang, D., Garcia-Bassets, I., Benner, C., Li, W., Su, X., Zhou, Y., Qiu, J., Liu, W., Kaikkonen, M.U., Ohgi, K.A., et al. (2011). Reprogramming transcription by distinct classes of enhancers functionally defined by eRNA. Nature *474*, 390–394.

Weintraub, A.S., Li, C.H., Zamudio, A.V., Sigova, A.A., Hannett, N.M., Day, D.S., Abraham, B.J., Cohen, M.A., Nabet, B., Buckley, D.L., et al. (2017). YY1 is a structural regulator of enhancer-promoter loops. Cell *171*, 1573–1588.

Whyte, W.A., Orlando, D.A., Hnisz, D., Abraham, B.J., Lin, C.Y., Kagey, M.H., Rahl, P.B., Lee, T.I., and Young, R.A. (2013). Master transcription factors and mediator establish super-enhancers at key cell identity genes. Cell *153*, 307–319.

Yusufzai, T.M., Tagami, H., Nakatani, Y., and Felsenfeld, G. (2004). CTCF tethers an insulator to subnuclear sites, suggesting shared insulator mechanisms across species. Mol. Cell *13*, 291–298.

Zhang, X., Choi, P.S., Francis, J.M., Imielinski, M., Watanabe, H., Cherniack, A.D., and Meyerson, M. (2016). Identification of focally amplified lineage-specific super-enhancers in human epithelial cancers. Nat. Genet. *48*, 176–182.

Zuin, J., Dixon, J.R., van der Reijden, M.I.J.A., Ye, Z., Kolovos, P., Brouwer, R.W.W., van de Corput, M.P.C., van de Werken, H.J.G., Knoch, T.A., van IJcken, W.F.J., et al. (2014). Cohesin and CTCF differentially affect chromatin architecture and gene expression in human cells. Proc. Natl. Acad. Sci. USA *111*, 996–1001.

Cell Reports, Volume *23*

Supplemental Information

Transcriptional Dysregulation of *MYC* Reveals Common Enhancer-Docking Mechanism

Jurian Schuijers, John Colonnese Manteiga, Abraham Selby Weintraub, Daniel Sindt Day, Alicia Viridiana Zamudio, Denes Hnisz, Tong Ihn Lee, and Richard Allen Young

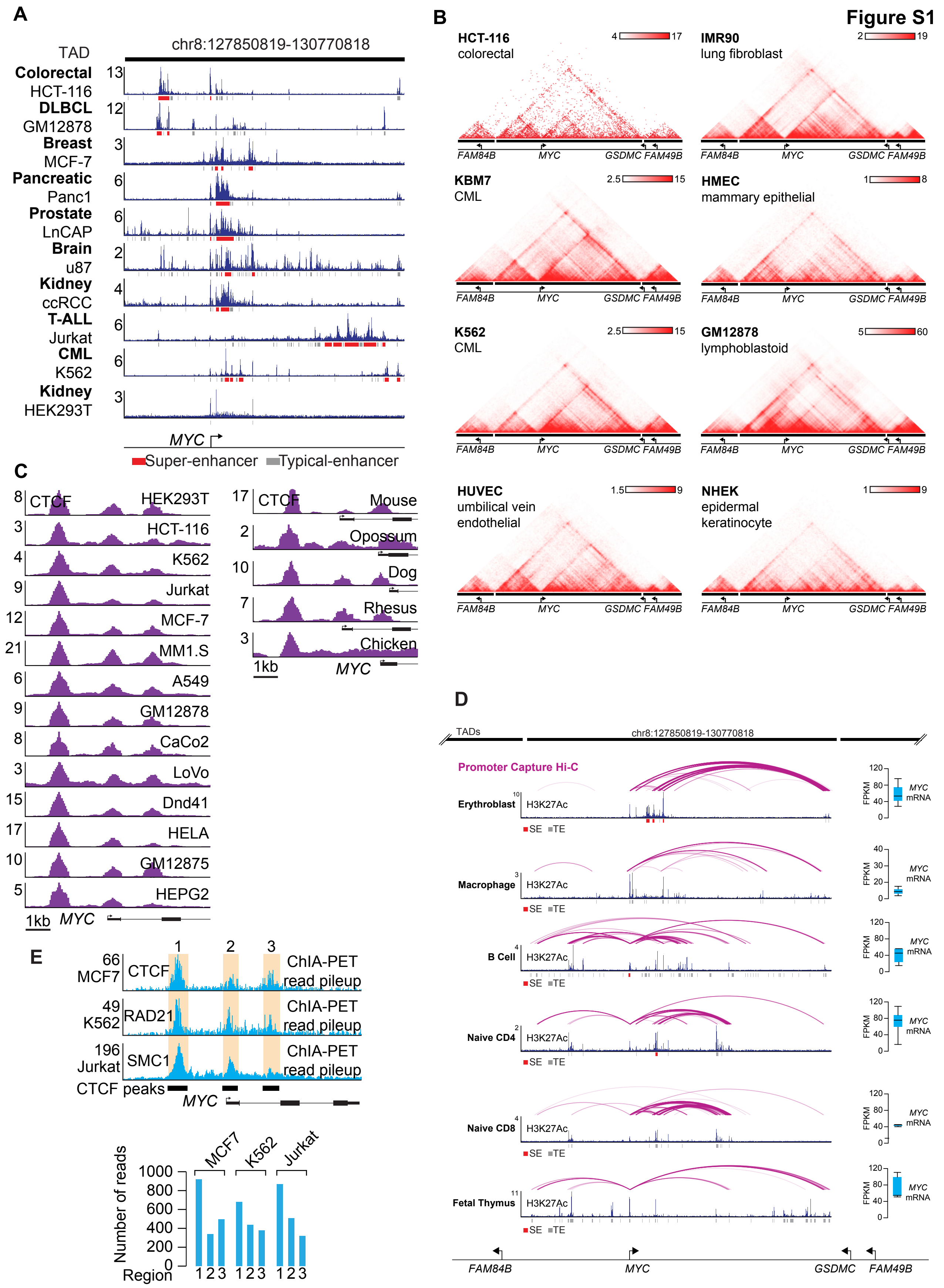

Figure S1
A
TAD
chr8:127850819-130770818
Colorectal HCT-116
DLBCL GM12878
Breast MCF-7
Pancreatic Panc1
Prostate LnCAP
Brain u87
Kidney ccRCC
T-ALL Jurkat
CML K562
Kidney HEK293T
MYC
Super-enhancer
Typical-enhancer
B
HCT-116 colorectal
IMR90 lung fibroblast
KBM7 CML
HMEC mammary epithelial
K562 CML
GM12878 lymphoblastoid
HUVEC umbilical vein endothelial
NHEK epidermal keratinocyte
FAM84B MYC GSDMC FAM49B
C
CTCF
HEK293T
HCT-116
K562
Jurkat
MCF-7
MM1.S
A549
GM12878
CaCo2
LoVo
Dnd41
HELA
GM12875
HEPG2
Mouse
Opossum
Dog
Rhesus
Chicken
1kb
MYC
D
TADs
chr8:127850819-130770818
Promoter Capture Hi-C
Erythroblast
Macrophage
B Cell
Naive CD4
Naive CD8
Fetal Thymus
H3K27Ac
SE TE
FPKM
MYC mRNA
FAM84B MYC GSDMC FAM49B
E
MCF7 CTCF ChIA-PET read pileup
K562 RAD21 ChIA-PET read pileup
Jurkat SMC1 ChIA-PET read pileup
CTCF peaks
MYC
Number of reads
Region
MCF7 K562 Jurkat

Figure S1. Cell type-specific super-enhancers in the *MYC* locus loop to a common CTCF site. Related to Figure 1.

(**A**) The 2.8 Mb TAD containing the *MYC* gene is indicated with thick black horizontal lines. H3K27Ac ChIP-seq signal (reads per million sequenced reads per base pair, data from this manuscript, (Becket et al., 2016; Frietze et al., 2012; Lin et al., 2012; Pope et al., 2014; Wang et al., 2011)) is shown in dark blue for a panel of tumor cell lines that express *MYC*. Tumor super-enhancers in the *MYC* TAD are depicted as red rectangles and typical enhancers are depicted as grey rectangles. (**B**) Heatmap of the ORIGAMI processed HiChIP, unfiltered data showing the *MYC* TAD with flanking regions (chr8:127100000-131525000) and Heatmaps of Hi-C interaction data showing the *MYC* TAD with flanking regions (chr8:127100000-131525000) across seven different cell types (data from (Rao et al., 2014)). The HiChIP was not smoothened as opposed to the smoothened HiC data. Scale bars represent the contrast settings used, numbers indicate the maximum intensity cutoff. The color intensity represents the PET count and the cutoff is represented in PET numbers for the HiChIP data. (**C**) CTCF ChIP-seq across a panel of tumor cell lines (data from (Anders et al., 2014; Encode Consortium, 2012; Hnisz et al., 2016; Pope et al., 2014; Wang et al., 2012; Yan et al., 2013)), and from mouse T-helper cells, Opossum, Dog, and Rhesus macaque liver (data from (Schmidt et al., 2012; Stadler et al., 2011)). Read counts are shown in reads per million sequenced reads per base pair.

(**D**) Promoter Hi-C interaction data and H3K27Ac ChIP-seq at the *MYC* TAD for cell types that represent different stages in hematopoietic development. The 2.8 Mb TAD containing *MYC* and part of the two adjacent TADs are indicated with thick black horizontal lines. Promoter Hi-C interactions are shown as purple colored arcs; the intensity of purple color reflects the confidence score from (Javierre et al., 2016). H3K27Ac ChIP-seq signal is shown, measured in reads per million sequenced reads per base pair (data from(Bernstein et al., 2010; Encode Consortium, 2012; Schmidt et al., 2016; Xu et al., 2012)). Super-enhancers are depicted as red rectangles and typical enhancers as grey rectangles. The relative level of *MYC* transcripts in the corresponding cell types are shown as boxplots in fragments per kilobase of exon per million sequenced reads (FPKM), expression data from the BLUEPRINT consortium, fetal thymus expression data from the ENCODE consortium. (**E**) ChIA-PET read pileups at the *MYC* promoter and quantification of the reads in the three CTCF peaks indicated. Light blue tracks display the read counts from read pileups of MCF7 CTCF, K562 RAD21 and Jurkat SMC1 ChIA-PET data showing that the majority of the ChIA-PET reads are found at the enhancer-docking site. Reads are shown as read counts per base pair.

Figure S2

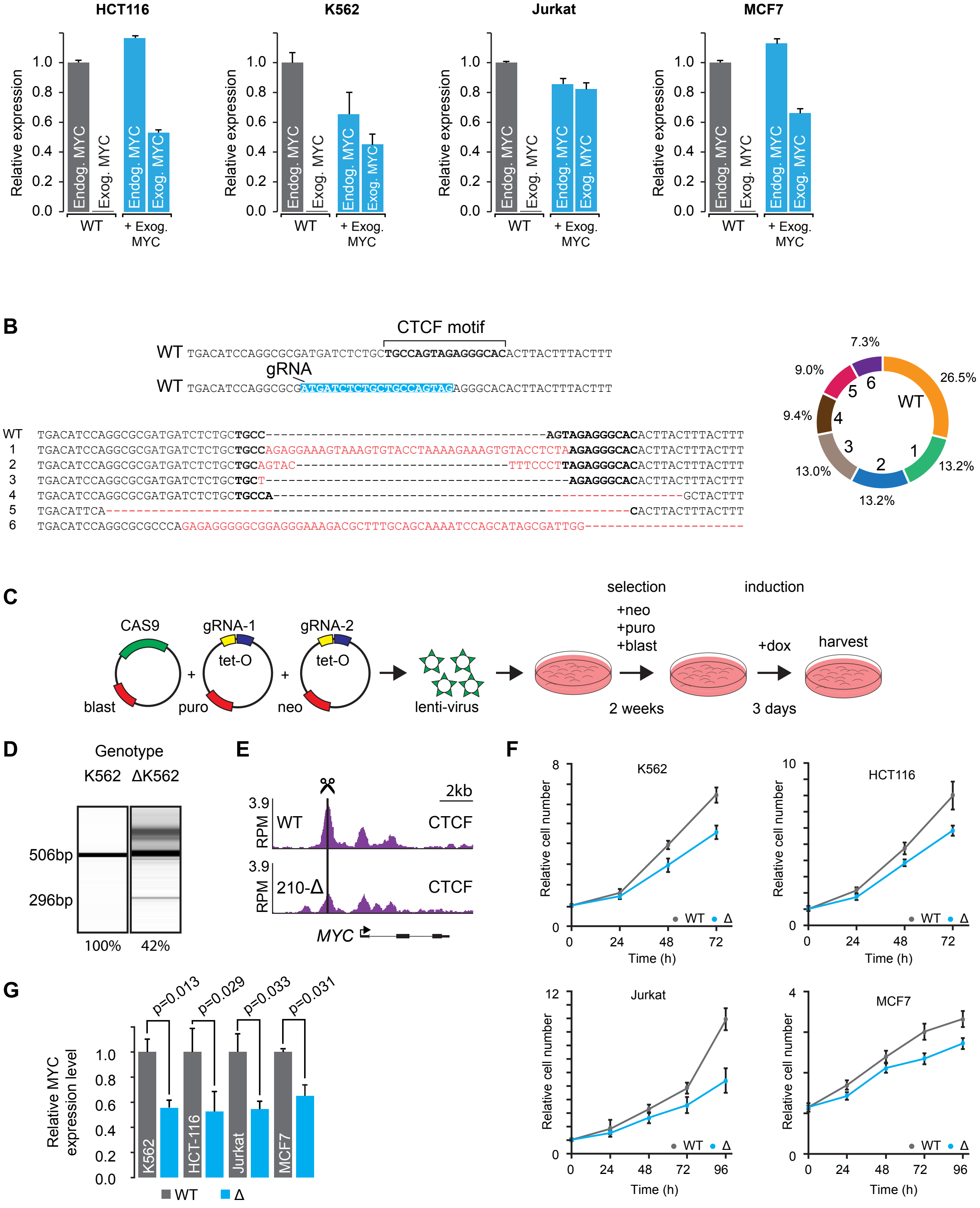

A
HCT116
K562
Jurkat
MCF7
Relative expression
1.0
0.8
0.6
0.4
0.2
0.0
Endog. MYC
Exog. MYC
WT
+ Exog. MYC
B
CTCF motif
WT TGACATCCAGGCGCGATGATCTCTGCTGCCAGTAGAGGGCACACTTACTTTACTTT
gRNA
WT TGACATCCAGGCGCGATGATCTCTGCTGCCAGTAGAGGGCACACTTACTTTACTTT
WT TGACATCCAGGCGCGATGATCTCTGCTGCC-----AGTAGAGGGCACACTTACTTTACTTT
1 TGACATCCAGGCGCGATGATCTCTGCTGCCAGAGGAAAGTAAAGTGTACCTAAAAGAAAGTGTACCTCTAAGAGGGCACACTTACTTTACTTT
2 TGACATCCAGGCGCGATGATCTCTGCTGCAGTAC-----TTTCCCTTAGAGGGCACACTTACTTTACTTT
3 TGACATCCAGGCGCGATGATCTCTGCTGCT-----AGAGGGCACACTTACTTTACTTT
4 TGACATCCAGGCGCGATGATCTCTGCTGCCA-----GCTACTTT
5 TGACATTCA-----CACTTACTTTACTTT
6 TGACATCCAGGCGCGCCCAGAGAGGGGGCGGAGGGAAAGACGCTTTGCAGCAAAATCCAGCATAGCGATTGG-----
7.3%
26.5%
9.0%
9.4%
13.0%
13.2%
13.2%
WT
1
2
3
4
5
6
C
CAS9
gRNA-1
gRNA-2
tet-O
blast
puro
neo
lenti-virus
selection
+neo
+puro
+blast
2 weeks
induction
+dox
3 days
harvest
D
Genotype
K562
ΔK562
506bp
296bp
100%
42%
E
2kb
3.9
RPM
WT
CTCF
210-Δ
MYC
F
K562
HCT116
Jurkat
MCF7
Relative cell number
Time (h)
WT
Δ
G
p=0.013
p=0.029
p=0.033
p=0.031
Relative MYC expression level
K562
HCT-116
Jurkat
MCF7
WT
Δ

Figure S2. Perturbation of the core CTCF motif in the *MYC* CTCF loop-anchor reduces CTCF occupancy and *MYC* expression. Related to Figure 2.

(**A**) qPCR measuring the mRNA levels of endogenous (endog.) and exogenous (exog.) *MYC* in parental (wild type) and exogenous *MYC* expressing HCT-116, K562, MCF7 and Jurkat cells. Endogenous and exogenous *MYC* were detected using primers directed against the 3' UTR of the *MYC* mRNA and the *MYC*-tdTomato junction respectively. (**B**) Sequencing of mutant alleles in the selected MCF7 clone with mutated enhancer-docking site. The CRISPR targeted region was amplified, fragmented and sequenced to identify the composition and frequency of mutant alleles. The 6 most common mutant alleles are displayed. (**C**) Perturbation of *MYC* promoter proximal CTCF site reduces *MYC* expression and proliferation rate across cancers. Schematic representation of the experiment. Cells were transduced with one virus carrying Cas9 and two viruses each carrying one guide RNA (gRNA) under a doxycycline inducible promoter. After selection for all three components, cells were induced with doxycycline for 3 days prior to harvest and testing. (**D**) Heat map of fragment lengths after genotyping PCR of wild type K562 and ΔK562 cells. PCR product was analyzed with a Fragment Analyzer and fragments of different lengths were quantified. The percentage of fragments with a length expected from wild type cells (506) is indicated under the lanes. The expected length of the PCR product for deleted and recombined alleles is 296bp. (**E**) ChIP-seq of CTCF in induced or uninduced K562 cells targeting the 210bp fragment containing the -2kb CTCF site. The -2kb CTCF binding is reduced, while the other CTCF binding sites in the *MYC* locus are unaffected. (**F**) Proliferation of parental (grey) and CTCF site deleted (blue) K562, HCT-116, Jurkat and MCF7 cells. Error bars represent the standard deviation of the mean from six biological replicates. (**G**) qPCR showing the *MYC* mRNA levels after deletion of the CTCF site in K562, HCT-116, Jurkat and MCF7 cells. p-values were generated with a Students T-test. Error bars represent the standard deviation of the mean from three biological replicates.

Figure S3

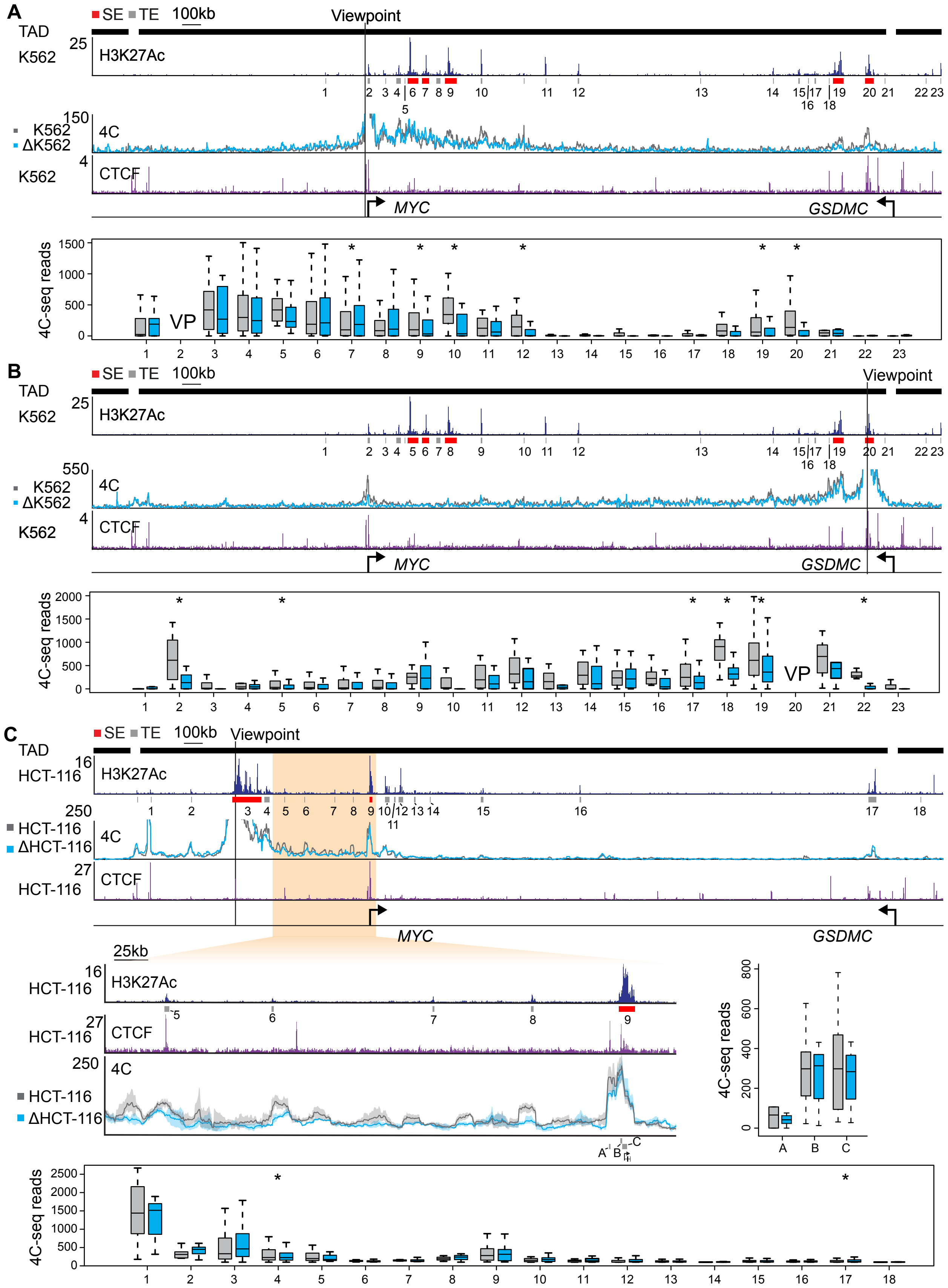
A
SE
TE
100kb
Viewpoint
TAD
K562
H3K27Ac
4C
ΔK562
CTCF
MYC
GSDMC
4C-seq reads
VP
B
C
HCT-116
ΔHCT-116
25kb

Figure S3. Perturbation of the *MYC* enhancer-docking site reduces looping to super-enhancers. Related to figure 3.

(**A**) 4C analysis showing quantification of regions based on H3K27Ac ChIP-seq peak calls for the 4C experiment with the viewpoint (VP) at the -2kb upstream CTCF site in K562 cells. (**B**) 4C analysis showing quantification of regions based on H3K27Ac ChIP-seq peak calls for the 4C experiment with the viewpoint (VP) at downstream super-enhancer in K562 cells. (**C**) 4C analysis showing quantification of regions based on H3K27Ac or CTCF ChIP-seq peak calls for the 4C experiment with the viewpoint (VP) at upstream super-enhancer in HCT-116 cells. Blowup shows the 4C interactions for the intervening region between the super-enhancer and the *MYC* gene. H3K27Ac ChIP-seq and CTCF ChIP-seq are shown in blue and purple, respectively. Shading represents the 90% confidence interval based on three biological replicates. Grey bars indicate the regions that are quantified. Box plots show quantification of the reads per fragment for the indicated regions. p-values were generated using Students T-test and data pairs with a p-value of < 0.05 are indicated with an asterisk. Reads are shown in reads per million sequenced reads per base pair. Typical-enhancers and super-enhancers are shown as grey boxes and red boxes respectively.

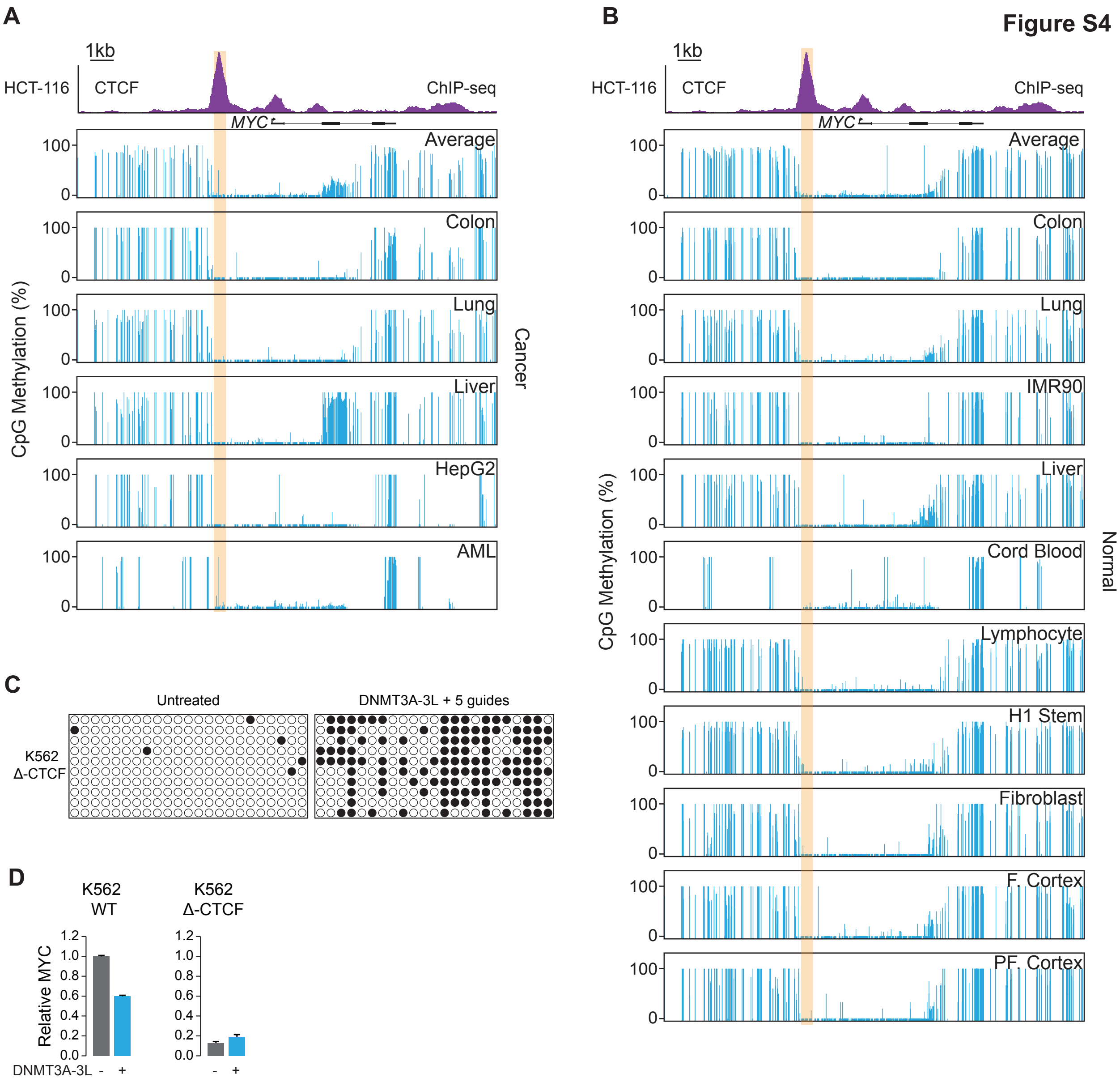

Figure S4. The *MYC* CTCF loop-anchor site is hypomethylated in a swathe of cancer and normal cells. Loss of *MYC* expression upon docking site methylation is dependent on presence of CTCF. Related to figure 4.
(**A**) Percentage of methylation of CpG's at the *MYC* locus in cancer cells. Percent methylation of each CpG in the region for which data was available for is represented as a blue line. HCT-116 CTCF ChIP-seq signal is shown in purple with the *MYC* promoter proximal CTCF site highlighted in yellow for reference. ChIP-seq read counts are shown in reads per million sequenced reads per base pair. (**B**) Percentage of methylation of CpG's at the *MYC* locus in normal cells. Percent methylation of each CpG in the region for which data was available for is represented as a blue line. Whole genome bisulfite sequencing data from ENCODE, (Barabé et al., 2016; Ziller et al., 2013). (**C**) Methylation at *MYC* promoter loop-anchor site in untreated and dCas9-DNMT3A-3L + 5 gRNA transfected K562 Δ-CTCF cells. (**D**) qPCR analysis of *MYC* mRNA levels for untreated and dCas9-DNMT3A-3L + 5 gRNA transfected K562 WT cells (data from Figure 4D) and K562 Δ-CTCF cells. Error bars represent the standard deviation of the mean for three biological replicates.

Figure S5

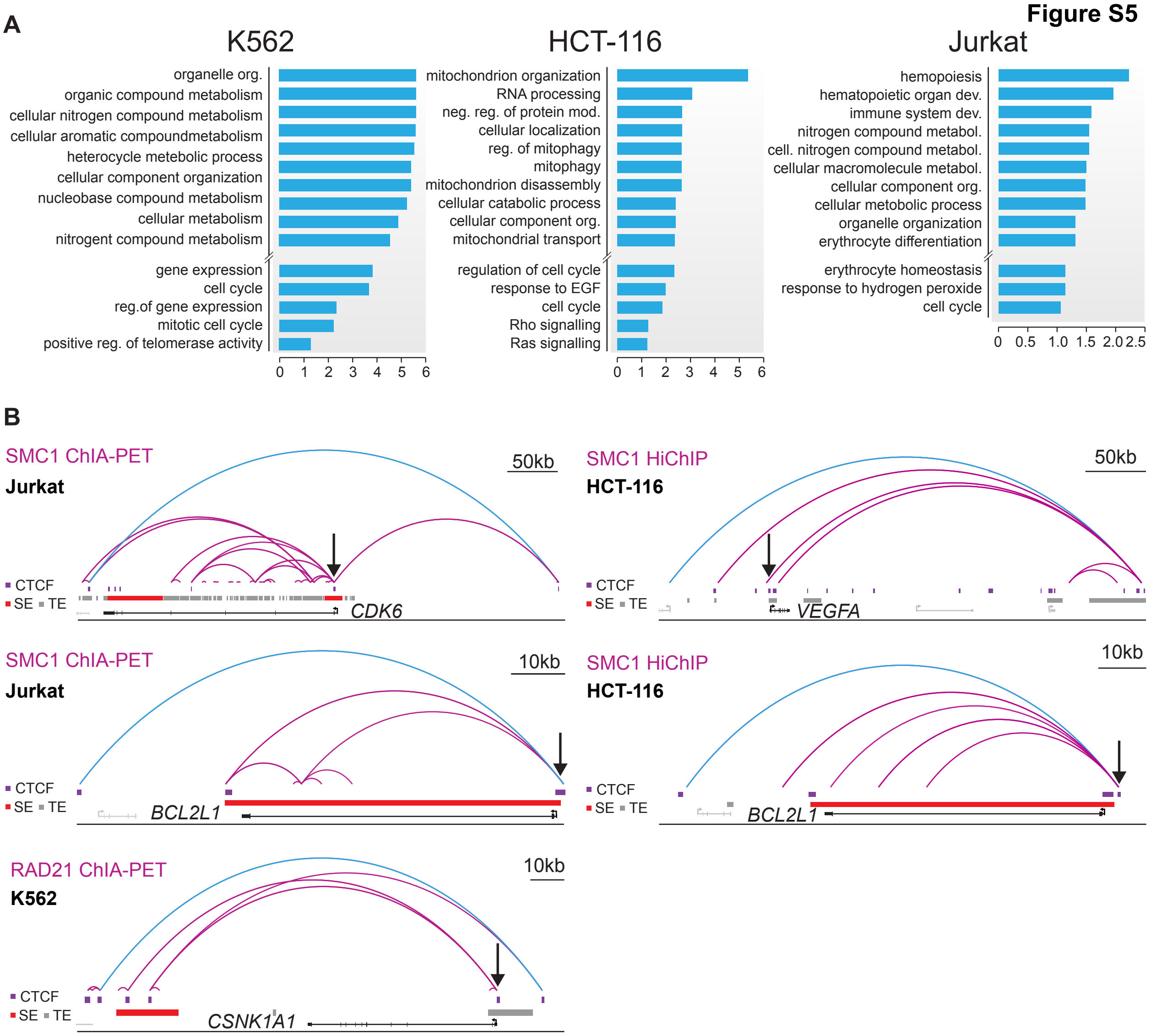

Figure S5. Putative enhancer-docking sites occur at additional genes with prominent roles in cancer. Related to figure 5.

(**A**) Gene ontology analysis of the genes with putative CTCF enhancer-docking in K562, HCT-116 and Jurkat cells. Blue bars indicate the –log10 of the q-value associated with the enrichment of the GO term indicated. The top ten most enriched GO terms are shown in addition to selected cancer associated GO terms that are significantly enriched (q-value < 0.1). (**B**) Displays of additional cancer associated genes with putative CTCF enhancer-docking. Black arrows indicate the CTCF sites that may facilitate enhancer-docking. CTCF ChIP-seq peaks are shown as purple rectangles, typical enhancers are shown as grey rectangles and super-enhancers are shown as red rectangles. The *MYC* insulated neighborhood loop is shown in blue and loops internal to it are shown in purple. ChIA-PET data used is indicated in purple lettering.

Supplemental Experimental Procedures

CONTACT FOR REAGENT AND RESOURCE SHARING

Further information and requests for resources and reagents should be directed to and will be fulfilled by the Lead Contact, Rick Young (young@wi.mit.edu). The Whitehead Institute has filed a patent application based on the work described in this manuscript.

RESOURCE TABLE

REAGENT or RESOURCE	SOURCE	IDENTIFIER
Antibodies		
H3K27Ac	Abcam	4729
SMC1	Bethyl	A300-055A
CTCF	Millipore	07729
Beta-Actin	Sigma	A5441
Chemicals, Peptides, and Recombinant Proteins		
Doxycycline	Sigma Aldrich	D9891
polybrene	Millipore	TR-1003
Critical Commercial Assays		
EpiTect Bisulfite Kit	Qiagen	59104
TruSeq DNA Sample Preparation v2 Kit	Illumina	RS-122-2001
Nextera DNA Library Preparation Kit	Illumina	FC-121-1030
Expand Long Template Polymerase	Roche	11759060001
RNeasy plus	Qiagen	74134
TaqMan Universal PCR Master Mix	Applied Biosystems	4304437
Power SYBR Green Master Mix	Applied Biosystems	4368577
Deposited Data		
4C	This study	GSE92880
HIChIP	This study	GSE92881
ChIP-seq	This study	GSE92879
Experimental Models: Cell Lines		
HCT-116	ATCC	CCL-243
K562	ATCC	HTB-22
MCF7	ATCC	CCL-247
Jurkat	ATCC	TIB-152
HEK293T	ATCC	CRL-3216
Oligonucleotides		
See table S4		
Recombinant DNA		
dCAS9-DNMT3A-3L	This study	
px330-Cas9	Zhang Lab	Addgene: 42230
DNMT3A-3L	Jeltsch Lab	
pLentiGuidePuro		Addgene: 52963
pAW21	This study	
pAW22	This study	

psPAX	Didier Trono	Addgene 12260
pMD2.G	Didier Trono	Addgene 12259
Software and Algorithms		
Origami	This study/ (Weintraub et al., 2017)	https://github.com/younglab/origami
ROSE	(Whyte et al., 2013)	https://bitbucket.org/young_computation/rose
4C Fourfold	This Study	https://github.com/younglab/fourfold
Bowtie	(Langmead et al., 2009)	http://bowtiebio.sourceforge.net/index.shtml
Samtools	(Li et al., 2009)	http://samtools.sourceforge.net
MACS	(Zhang et al., 2008)	http://liulab.dfci.harvard.edu/MACS/index.html
BEDTools	(Quinlan and Hall, 2010)	http://bedtools.readthedocs.io
UCSC Genome Browser	(Kent et al., 2002)	http://genome.ucsc.edu/cgibin/hgGateway
WASHU EpiGenome browser	(Zhou et al., 2011)	http://epigenomegateway.wustl.edu/
3D Genome viewer	(Wang et al., 2017)	http://www.3dgenome.org
Kallisto	(Bray et al., 2016)	https://pachterlab.github.io/kallisto/

EXPERIMENTAL MODEL AND SUBJECT DETAILS

Cell Lines

K562 (female), MCF7 (female), HCT-116 (male) and Jurkat (male) cells were purchased from ATCC (CCL-243, HTB-22, CCL-247, TIB-152) and propagated according to ATCC guidelines in RPMI-1640 with GlutaMax (Life Technologies 61870-127) or DMEM, high glucose, pyruvate (Life Technologies 11995-073), supplemented with 10% fetal bovine serum (Sigma). Cells were maintained at 37°C and 5% CO_2.

METHOD DETAILS

CRISPR/Cas9 genome editing

Genome editing was performed using CRISPR/Cas9 essentially as described (Ran et al., 2013; Shalem et al., 2014). The genomic sequences complementary to all guide RNAs are listed in Table S4

For generation of the *MYC* CTCF site deletion clones, target-specific oligonucleotides were cloned into the pX330 plasmid which carries a U6 promoter, chimeric guide RNA, and a codon-optimized version of Cas9. pX330 was a kind gift of F. Zhang (Ling et al., 2012) (Addgene: 42230). Using this construct, 2 million cells (expressing the exogenous

copy of *MYC*) were transfected with 10 ug of DNA with 50 uL of 1mg/ml PEI and sorted for presence of GFP after 2 days. Individual cells were then propagated in to clonal lines.

For the inducible CRISPR/Cas9 CTCF perturbation experiments (Figure S2C-G), target specific oligonucleotides were cloned into a plasmid containing the chimeric RNA, a doxycycline inducible H1 promoter, TetR, and a selectable marker (pAW 21 or pAW 22). Three separate viruses were produced containing pAW21::up guide, pAW22::down guide, and pLentiCas9-blast (Addgene: 52962, a kind gift of F. Zhang (Sanjana et al., 2014)). Stable cell lines were generated (see section on virus production and cell line generation) and then genome editing was induced by the addition of doxycycline (Sigma Aldrich D9891) at 1 ug/mL. Cells were induced for 72 hours and every 24 hours fresh doxycycline was spiked in.

<u>Virus production and generation of cell lines</u>
For virus production, HEK293T cells were grown to 50-75% confluency on a 15 cm dish and then transfected with 15 µg plasmid of interest, 11.25 µg psPAX (Addgene 12260), and 3.75 µg pMD2.G (Addgene 12259). psPAX and pMD2.G were kind gifts of Didier Trono. After 12 hours, media was replaced. Viral supernatant was collected 24 hours after media replacement (36 hrs post transfection) and fresh media was added. Viral supernatant was collected again 48 hours after the media replacement (60 hours post transfection). Viral supernatant was cleared of cells by either centrifugation at 500 x g for 10 minutes or filtration through a 0.45-micron filter. The virus was concentrated with Lenti-X concentrator (Clonetech 631231) per manufacturers' instruction. Concentrated virus was resuspended in either DMEM or RPMI (depending on the cell line being infected) and added to 5 million cells in the presence of polybrene (Millipore TR-1003) at 8 ug/mL. After 24 hours, viral media was removed and fresh media containing drug was added. Drug concentrations are as follows: Puromycin (Thermo A1113802) (2 ug/mL), Geneticin (Thermo 10131027) (800 ug/mL), Blasticidin (Invivogen ant-bl-1) (10 ug/mL). Cells were selected until all cells on non-transduced plates died.

<u>RNA isolation and quantitative RT-PCR</u>
RNA was isolated using the RNeasy, RNeasy plus or AllPrep kit (QIAGEN 74004, 80204) and reverse transcribed using oligo-dT primers (Promega C1101) and SuperScript III reverse transcriptase (Thermo 18080093) according to the manufacturers' instructions. Quantitative real-time PCR was performed on a 7000 AB Detection System using Taqman probes for *MYC* (Hs00153408_m1) and GAPDH (Hs02758991_g1) in conjunction with Taqman 2x master mix (Thermo 4304437). For detection of endogenous *MYC* only in experiments utilizing cells expressing the exogenous copy of *MYC*, primers specific to the endogenous copy of *MYC* (Table S4) were designed against a *MYC* 3' UTR region not present in the exogenous construct and qPCR was conducted with SYBR green PCR master mix (Thermo 4309155).

<u>ChIP-seq</u>

ChIP was performed as described in (Lee et al., 2006) with a few adaptations. ~30 million K562 cells were crosslinked for 10 min at room temperature by the addition of one-tenth of the volume of 11% formaldehyde solution (11% formaldehyde, 50 mM HEPES pH 7.3, 100 mM NaCl, 1 mM EDTA pH 8.0, 0.5 mM EGTA pH 8.0) to the growth media followed by 5 min quenching with 125 mM glycine. Cells were washed twice with PBS, then the supernatant was aspirated and the cell pellet was flash frozen at -80C. 100µl of Protein G Dynabeads (Thermo 10003D) were blocked with 0.5%BSA (w/v) in PBS. Magnetic beads were bound with 40 µl of anti-CTCF antibody (Millipore 07-729). Nuclei were isolated as previously described (Lee et al., 2006), and sonicated in lysis buffer (20 mM Tris-HCl pH 8.0, 150 mM NaCl, 2 mM EDTA pH8.0, 0.1% SDS, and 1% Triton X-100) on a Misonix 3000 sonicator for 5 cycles at 30s each on ice (18-21 W) with 60 s on ice between cycles. Sonicated lysates were cleared once by centrifugation and incubated overnight at 4°C with magnetic beads bound with antibody to enrich for DNA fragments bound by the indicated factor. Beads were washed with wash buffer A (50 mM HEPES-KOH pH7.9, 140 mM NaCl, 1 mM EDTA pH 8.0, 0.1% Na-Deoxycholate, 1%Triton X-100, 0.1% SDS), B (50 mM HEPES-KOH pH7.9, 500 mM NaCl, 1 mM EDTA pH 8.0,0.1% Na-Deoxycholate, 1% Triton X-100, 0.1% SDS), C (20 mM Tris-HCl pH8.0, 250 mMLiCl, 1 mM EDTA pH 8.0, 0.5% Na-Deoxycholate, 0.5% IGEPAL C-630 0.1% SDS) and D (TE with 50 mM NaCl) sequentially. DNA was eluted in elution buffer (50 mM Tris-HCL pH 8.0, 10mM EDTA, 1% SDS). Cross-links were reversed overnight at 65°C. RNA and protein were digested using RNase A and Proteinase K, respectively and DNA was purified with phenol chloroform extraction and ethanol precipitation. Purified ChIP DNA was used to prepare Illumina multiplexed sequencing libraries. Libraries for Illumina sequencing were prepared following the Illumina TruSeq DNA Sample Preparation v2 kit. Amplified libraries were size-selected using a 2% gel cassette in the Pippin Prep system from Sage Science set to capture fragments between 200 and 400 bp. Libraries were quantified by qPCR using the KAPA Biosystems Illumina Library Quantification kit according to kit protocols. Libraries were sequenced on the Illumina HiSeq 2500 for 40 bases in single read mode.

<u>4C-seq</u>
A modified version of 4C-seq (van de Werken et al., 2012a, 2012b) was developed. The major change was the ligation is performed in intact nuclei (in situ). This change was incorporated because previous work has noted that in situ ligation dramatically decreases the rate of chimeric ligations and background interactions (Nagano et al., 2015; Rao et al., 2014).

Approximately 5 million K562 cells were resuspended in 5 mL 10% FBS/PBS. 5 mL of 4% formaldehyde in 10% FBS/PBS was added and cells were crosslinked for 10 minutes while rotating at room temperature. Glycine was added to a final concentration of 0.125 M and cells were centrifuged at 300xg for 5 minutes. Cells were washed twice with PBS, transferred to an eppendorf tube, snap frozen and stored at -80. Pellets were gently resuspended in Hi-C lysis buffer (10 mM Tris-HCl pH 8, 10 mM NaCl, 0.2%

Igepal) with 1x protease inhibitors (Roche, 11697498001). Cells were incubated on ice for 30 minutes then washed once with 500 uL of ice-cold Hi-C lysis buffer with no protease inhibitors. Pellets were resuspended in 50 uL of 0.5% SDS and incubated at 62°C for 7 minutes. 145 uL of water and 25 uL of 10% Triton X-100 were added and tubes incubated at 37°C for 15 minutes. 25 uL of 10X New England Biolabs CutSmart buffer and 200 units of NlaIII (NEB R0125L) enzyme were added and the chromatin was digested for four hours at 37 degrees in a thermomixer at 500 RPM. 200 additional units of NlaIII was spiked in and digest continued for 12 hours. Then, 200 additional units of NlaIII was spiked in and digest continued for four more hours. Restriction enzyme was inactivated by heating to 62°C for 20 minutes while shaking at 500 rpm. Proximity ligation was performed in a total of 1200 uL with 2000 units of T4 DNA ligase (NEB M0202M) for six hours at room temperature. After ligation samples were spun down for 5 minutes at 2500 g and resuspended in 300 uL 10 mM Tris-HCl, 1% SDS and 0.5 mM NaCl with 1000 units of Proteinase K. Samples were reversed cross-linked overnight at 68°C.

Samples were then phenol-chloroform extracted and ethanol precipitated and the second digestion was performed overnight in 450 uL with 50 units of CviQI (NEB R0639L). Samples were phenol-chloroform extracted and ethanol precipitated and the second ligation was performed in 14 mL total with 6700 units of T4 DNA ligase (NEB 0202M) at 16°C overnight. Samples were ethanol precipitated, resuspended in 500 uL Qiagen EB buffer, and purified with a Qiagen PCR kit.

The concentration was measured with a Nanodrop and PCR amplification was performed with 16 50 uL PCR reactions using Roche Expand Long Template polymerase (Roche 11759060001). Reaction conditions are as follows: 11.2 uL Roche Expand Long Template Polymerase, 80 uL of 10 X Roche Buffer 1, 16 uL of 10 mM dNTPs (Promega PAU1515), 112 uL of 10 uM forward primer, 112 uL of 10 uM reverse primer (Table S4), 200 ng template, and milli-q water till 800 uL total. Reactions were mixed and then distributed into 16 50 uL reactions for amplification. Cycling conditions were a “Touchdown PCR” based on reports that this decreases non-specific amplification of 4C libraries (Ghavi-Helm et al., 2014). The conditions are: 2’ 94°C, 10’’ 94°C, 1’ 63°C, 3’ 68 °C, repeat steps 2-4 but decrease annealing temperature by one degree, until 53°C is reached at which point reaction is cycled an additional 15 times at 53°C, after 25 total cycles are performed the reaction is held for 5’ at 68°C and then 4°C. Libraries were cleaned-up using a Roche PCR purification kit (Roche 11732676001) using 4 columns per library. Reactions were then further purified with Ampure XP beads (Agencourt A63882) with a 1:1 ratio of bead solution to library following the manufactures instructions. Samples were then quantified with Qubit and the KAPA Biosystems Illumina Library Quantification kit according to kit protocols. Libraries were sequenced on the Illumina HiSeq 2500 for 40 bases in single read mode.

HiChIP

HiChIP was performed essentially as described (Mumbach et al., 2016). 10 million HCT116 cells were crosslinked for 10 min at room temperature by the addition of one-tenth of the volume of 11% formaldehyde solution (11% formaldehyde, 50 mM HEPES pH 7.3, 100 mM NaCl, 1 mM EDTA pH 8.0, 0.5 mM EGTA pH 8.0) to the growth media followed by 5 min quenching with 125 mM glycine. Cells were washed twice with PBS, then the supernatant was aspirated and the cell pellet was flash frozen in liquid nitrogen. Frozen crosslinked cells were stored at −80°C.

The crosslinked pellets were thawed on ice, resuspended in 500 μL of ice-cold Hi-C Lysis Buffer (10mM Tris-HCl pH 8.0, 10 mM NaCl, 0.2% NP-40 with protease inhibitors), and rotated at 4ºC for 30 minutes. Nuclei were spun down at 2500 rcf for 5 minutes at 4ºC, and washed once with 500 μL of ice-cold Hi-C Lysis Buffer. Supernatant was removed, and the pellet was resuspended in 100 μL of 0.5% SDS. Nuclei were incubated at 62ºC for 7 minutes, and SDS was quenched by addition of 285 μL of H_2O and 50 μL of 10% Triton X-100 for 15 minutes at 37ºC. After the addition of 50 μL of 10X NEB Buffer 2 and 400U of MboI restriction enzyme (NEB, R0147), chromatin was digested overnight at 37ºC. The following day, the MboI enzyme was inactivated by incubating the nuclei at 62ºC for 20 minutes.

To fill in the restriction fragment overhangs and mark the DNA ends with biotin, the following was added: 37.5 μL 0.4 mM biotin-ATP (19524-016, Invitrogen) 1.5 μL of 10 mM dCTP (N0441S, NEB), 1.5 μL of 10 mM dTTP (N0443S, NEB), 1.5 μL of 10 mM dGTP (N0442S, NEB), 10 μL of 5U/μL DNA Polymerase I, Large (Klenow) Fragment (NEB, M0210), and the tubes were incubated at 37ºC for 1 hour with rotation. Next, the following mix was added for the proximity ligation step: 150 μL of 10X NEB T4 DNA ligase buffer with 10 mM ATP (NEB, B0202), 125 μL 10% Triton X-100, 3 μL 50 mg/mL BSA, 10 μL 400 U/μL T4 DNA Ligase (NEB, M0202), 660 μL H_2O, and the nuclei suspension was incubated at room temperature for 6 hours with rotation. Nuclei were pelleted at 2500 rcf for 5 minutes and supernatant was removed.

Pellets were resuspended in 880 μL in Nuclear Lysis Buffer (50mM Tris-HCl pH 7.5, 10 mM EDTA, 1% SDS with protease inhibitors), and the lysates were sonicated on a Covaris S220 instrument using the following parameters: Fill Level 10, Duty Cycle 5, PIP 140, Cycles/Burst 200, for 4 minutes. Sonicated lysates were spun down at 16100 rcf for 15 minutes at 4oC, and the supernatant was transferred to a fresh tube. The supernatant was split into two Eppendorf tubes (about 400 μL of lysate in each), and 800 μL of ChIP dilution buffer (0.01% SDS, 1.1% Triton X-100, 1.2 mM EDTA, 16.7 mM Tris pH 7.5, 167 mM NaCl) was added to each tube. 60 μL of Protein G beads (Life Technologies) were washed in ChIP dilution buffer, resuspended in 100 μL ChIP dilution buffer and 50 μL were added to each of the two tube of lysates. Tubes were then rotated for 1 hour at 4ºC to preclear the lysates. Dynabeads were separated on a magnetic stand, and the supernatant was moved to a fresh tube. 3.5 μg of SMC1A antibody (Bethyl A300-055A) were added to each tube, and tubes were incubated at 4ºC overnight with rotation. The next day, 60 μL of Protein G beads were washed ChIP Dilution Buffer, resuspended in 100 μL ChIP Dilution Buffer, and 50 μL was added to

each sample tube. Samples were then incubated for 2 hours at 4°C with rotation. Beads were washed twice with Low Salt Wash Buffer (0.1% SDS, 1% Triton X-100, 2 mM EDTA, 20 mM Tris-HCl pH 7.5, 150 mM NaCl), twice with High Salt Wash Buffer (0.1% SDS, 1% Triton X-100, 2 mM EDTA, 20 mM Tris-HCl pH 7.5, 500 mM NaCl), twice with LiCl buffer (10 mM Tris pH 7.5, 250 mM LiCl, 1% NP-40, 1% Sodium-deoxycholate, 1 mM EDTA). Beads were then resuspended in 100 µL of DNA Elution Buffer (50 mM $NaHCO_3$, 1% SDS), incubated for 10 minutes at room temperature with rotation and 3 minutes at 37°C with shaking. Beads were separated on a magnetic stand, and supernatant was transferred to a fresh tube. Beads were then mixed with another 100 µL of DNA Elution Buffer, incubated for 10 minutes at room temperature with rotation and 3 minutes at 37°C with shaking. Beads were separated on a magnetic stand, and supernatant was combined with the previous round of supernatant. 10 µL of Proteinase K (20mg/ml) was added to each sample and samples were incubated at 55°C for 45 minutes with shaking. Temperature was then increased to 67°C, and samples were incubated for 1.5 hours with shaking. Samples were purified on a Zymo column (Zymo Research).

Fragmentation of the ChIP DNA was performed using the Tn5 transposase (Illumina). First, 5 µL of Streptavidin M-280 magnetic beads were washed with Tween Wash Buffer (5 mM Tris-HCl pH 7.5, 0.5 mM EDTA, 1 M NaCl, 0.05% Tween-20), resuspended in 10 µL of Binding Buffer (10 mM, Tris-HCl pH 7.5, 1 mM EDTA, 2 M NaCl), and added to the samples. Samples were then rotated for 15 minutes at room temperature. Beads were separated on a magnet, and supernatant was discarded. Beads were washed twice with 500 µL of Tween Wash Buffer and incubated at 55°C for 2 minutes shaking. Beads were then washed with 100 µL of 1X TD Buffer (Nextera DNA sample preparation kit, FC-121-1030, Illumina), and tagmented using the Nextera DNA sample preparation kit (FC-121-1030, Illumina). 0.5 µL TDE1 enzyme was used to tagment 10ng of ChIP DNA (quantified after the previous Zymo column purification). Tagmentation was performed for 10 minutes at 55°C with shaking. Beads were then separated on a magnet, and supernatant was discarded. Beads were washed with 50 mM EDTA at 50°C for 30 minutes, and twice with 50 mM EDTA at 50°C for 3 minutes. Beads were then washed twice in Tween Wash Buffer at 55°C for 2 minutes, and once with 10 mM Tris for 1 minute at room temperature. The tagmented library still bound to the beads was amplified by 12 cycles of PCR using the Nextera DNA sample preparation kit. The library was then purified on a Zymo column, size-selected (300-700 bp) using AMPure beads (Agencourt) per manufacturers' instructions, and sequenced 100x100 on an Illumina Hi-Seq 2500.

<u>Targeted methylation and bisulfite sequencing.</u>

To perform targeted methylation, HCT-116 cells or K562 cells were transfected with a dCas9-DNMT3A-3L construct and 5 guides. To generate the dCas9-DNMT3A-3L construct, dCas9 was isolated from pSQL1658 (Addgene: 51023) by PCR. Cas9 was removed from pX330-Cas9 (Addgene: 42230) by AgeI and EcoRI restriction digest.

dCas9 was inserted into pX330 to create pX330-dCas9. DNMT3A-3L carrier plasmid was a generous gift from the Jeltsch lab (Siddique et al., 2013). DNMT3A-3L was cloned into pX330-dCas9 using PmeI and AscI sites to create pX330-dCas9-DNMT3A-3L (no guides). Guide RNAs were added to pX330-dCas9-DNMT3A-3L by digesting pX330-dCas9-DNMT3A-3L with BbsI followed by ligation of annealed oligos (Table S4) to create pX330-dCas9-DNMT3A-3L-guide with three different guides. An additional plasmid containing two guides, lentiGuide-Puro-double_guide, was also generated. Double guide containing gBlock (individual guide sequences Table S4) was cloned into lentiGuide-Puro (Addgene: 52963) as described (Vidigal and Ventura, 2015) to create lentiGuide-Puro-double_guide. Two hundred fifty thousand HCT-116 cells were transfected with 250ng of pX330-dCas9-DNMT3A-3L-guide1, 250ng of pX330-dCas9-DNMT3A-3L-guide2, 250ng of pX330-dCas9-DNMT3A-3L-guide3, 250ng lentiGuide-Puro-double_guide, and 5 uL of 1mg/ml PEI, and harvested after two days. One million K562 cells were transfected with 2.5µg of pX330-dCas9-DNMT3A-3L-guide1, 2.5µg of pX330-dCas9-DNMT3A-3L-guide2, 2.5µg of pX330-dCas9-DNMT3A-3L-guide3, and 2.5µg lentiGuide-Puro-double_guide using the Neon transfection system (Invitrogen MPK10025) following the manufacturers protocol and electroporation settings as follows: 1450V, 10ms width, 3 pulses. HCT-116 and K562 cells were harvested and sorted for GFP presence after two days.

To detect methylation, 2ug of gDNA from HCT-116 or K562 cells untransfected or transfected with dCas9-DNMT3A-3L plus guides were bisulfite converted using the EpiTect Bisulfite Kit (QIAGEN 59104). Converted gDNA was eluted in 20uL H2O. Converted gDNA was PCR amplified with EpiMark® Hot Start Taq DNA Polymerase (NEB M0490) using 3uL of converted gDNA as template and locus specific primers (table S4). PCR was carried out as follows: 95°C for 30 sec; 95°C for 20 sec; 52°C for 30 sec; 68°C for 30 sec; repeat steps 2-4 45x; 68°C for 5 min; Hold 4°C. Resultant amplicons were cleaned up using QIAquick PCR Purification Kit (QIAGEN 28106) and eluted in 20ul H2O. Clean amplicons (3uL) were subcloned using the pGEM-T Easy vector system (Promega A1360) and transformed into DH5α competent cells. Individual colonies were then picked, and colony PCR was carried out using GoTaq Green Master Mix (Promega M712) with the same primers originally used for the converted gDNA amplification. Colony PCR was carried out as follows: 95°C for 2 min; 95°C for 45 sec; 51°C for 45 sec; 72°C for 45 sec; repeat steps 2-4 29x; 72°C 5 min; Hold 4°C. Resultant amplicons were then Sanger sequenced, and CpG methylation was detected as CpG sequences that were not converted to TpG. All converted DNA analyzed had greater than or equal to 95% bisulfite conversion rate.

Hi-C visualization

Hi-C datasets were visualized using the 3D Genome browser at http://www.3dgenome.org.

Visualization of ChIA-PET interactions on the WashU Genome Browser

The output of origami was visualized in the WashU genome browser by converting the output of origami into a WashU compatible format using *origami-conversion.*

Topoloogically Associating Domain (TAD) calls
TAD calls were taken from the TAD calls in (Dixon et al., 2012) from the H1 human embryonic cell line.

QUANTIFICATION AND STATISTICAL ANALYSIS

ChIP-seq data analysis
ChIP-Seq datasets were generated for this study as well as collated from previous studies (Table S5), and were aligned using Bowtie (version 0.12.2) (44) to the human genome (build hg19, GRCh37) with parameter -k 1 -m 1 -n 2. We used the MACS version 1.4.2 (model-based analysis of ChIP-seq) (45) peak finding algorithm to identify regions of ChIP-seq enrichment over input DNA control with the parameter "--no-model --keep-dup=auto". A p-value threshold of enrichment of 1e-09 was used. UCSC Genome Browser tracks were generated using MACS wiggle outputs with parameters "-w –S – space=50". The browser snapshots of the ChIP-Seq binding profiles displayed throughout the study use read per kilobase per million mapped reads dimension (rpm/bp) on the y-axis.

Identification of enhancers and super-enhancers
Enhancers and super-enhancers were identified using H3K27Ac ChIP-seq data as previously described (Hnisz et al., 2013). Briefly, enhancers were defined as H3K27Ac ChIP-Seq peaks identified using MACS. To identify super-enhancers, the H3K27Ac ChIP-Seq peaks (i.e. enhancers) were stitched together if they were within 12.5 kb, and the stitched enhancers were ranked by their ChIP-seq read signal of H3K27Ac, using the ROSE algorithm (https://bitbucket.org/young_computation/rose) (Lovén et al., 2013). ROSE separates super-enhancers from typical enhancers by identifying an inflection point of H3K27ac signal vs. enhancer rank (Hnisz et al., 2013; Lovén et al., 2013).

4C analysis
The 4C-seq samples were first processed by removing their associated read primer sequences (Table S4) from the 5' end of each FASTQ read. To improve mapping efficiency of the trimmed reads by making the read longer, the restriction enzyme digest site was kept on the trimmed read. After trimming the reads, the reads were mapped using bowtie with options –k 1 –m 1 against the hg19 genome assembly. All unmapped or repetitively mapping reads were discarded from further analysis. The hg19 genome was then "digested" in silico according to the restriction enzyme pair used for that sample to identify all the fragments that could be generated by a 4C experiment given a restriction enzyme pair. All mapped reads were assigned to their corresponding fragment based on where they mapped to the genome. The digestion of a sample in a 4C experiment creates a series of "blind" and "non-blind" fragments as described (van de Werken et al., 2012b). In a perfect experiment, we should have only observed reads

at non-blind fragments, and reads at blind fragments exhibit a much higher experimental variability than non-blind fragments, so we only used the reads from non-blind fragments for further analysis. To normalize the distribution of different samples, we quantile normalized all non-blind fragments in each sample together. If no reads were detected at a non-blind fragment for a given sample when reads were detected in at least one other sample, we assigned a “0” to that non-blind fragment for the sample(s) missing reads. After normalization, we then smoothed the normalized profile of each sample using a 6kb running mean at 500 bp steps across the genome. After smoothing, for each condition we combined the replicates of a condition by taking the mean signal of each bin across all replicates of the condition. Quantification of the 4C signal counted the reads per fragment per million sequenced reads in the indicated regions. For quantification the H3K27Ac stitched peaks from the ROSE algorithm were used (Hnisz et al., 2013) or the CTCF MACS peak calls, padded with 1kb on either side in order to include sufficient 4C fragments.

HiChIP data analysis

The HiChIP samples were processed by removing their associated read primer sequences from the 5’ end of each FASTQ read. Read pairs were separated and separate reads were mapped using bowtie with options –k 1 –m 1 against the hg19 genome assembly. Al unmapped or repetitively mapping read were discarded from further analysis. The hg19 genome was then divided in 50 kb bins and reads were joined back together in pairs (Paired End Tag PET). For every pair of bins the number of PETS joining them was then calculated. These data were then further analyzed by the ORIGAMI pipeline to identify significant bin to bin interaction pairs.

ChIA-PET data analysis and ORIGAMI description

We development a new software pipeline and analytical method called *origami* to process ChIA-PET. The software and releases can be found at https://github.com/younglab/origami using version alpha20160828. The ChIA-PET data sets analyzed along with their corresponding linker sequence and called interactions in and around the *MYC* TAD can be found in Table S4. Each ChIA-PET data sets was processed as follows: the reads were first trimmed and aligned using *origami-alignment,* which trims the ChIA-PET linker if present and aligns trimmed PETs. PETs not having a linker were discarded from further analysis. Each end of a PET with a linker sequence were separately mapped to the hg19 genome assembly using bowtie with the following options: -v 1 –k 1 –m 1. After alignment, the separated PETs were re-paired in the final BAM output. After repairing, all duplicated PETs within the data were removed, since these were believed to be PCR duplicates. Peaks were called on the re-paired ChIA-PET reads using MACS1 v1.4.2 with the following parameters: --nolambda –nomodel –p 1e-9.

We developed a novel analytical method to analyze ChIA-PET data that used a two-component Bayesian mixture model to accurately identify *in vivo* interactions from the

ChIA-PET data by accurately estimating the difference between the biological signal and technical and biological noise by controlling for error within the ChIA-PET protocol and linear genomic distance. We defined an *in vivo* interaction as two regions of the genome brought together in the nucleus longer than expected at random given the linear genomic distance between those two region. Our intuition was that true *in vivo* interactions would follow one distribution where experimental noise would arise from a separate distribution, and these two groups could be learned from the data using a mixture model. After alignment, we defined a set of putative contacts where a putative contact was any two MACS1 peaks linked together by at least one mapped PET. The PET count for a putative interaction was the total number of unique PETs mapped at both ends of the putative contact. All putative contacts and their PET count were used in the estimation. We estimated the distributions two-component mixture model (described more below) from these putative contacts using *origami-analysis*. We specifically designed the model to have the second mixture component represent the distribution of the *in vivo* interactions, and we tested whether the estimated group means (described below) were significantly different to validate that model found at least two different groups. After the estimation of each component, for each putative contact we estimate the posterior probability of whether the putative contact was within the distribution of the second component. If this posterior probability was greater than 0.9, we called this putative contact an *in vivo* interaction. We used this threshold because it was a good balance between what be believed to be a high true positive rate while minimizing the false positive rate in each sample analyzed (although we believe that are a few *in vivo* interactions below this threshold as well). In general, we often displayed all putative contacts within the *MYC* TAD by this posterior probability, eliminating the need for a specific cutoff threshold.

For the analytical model, we wanted to build a model that was able to estimate and control for noise arising from the ChIA-PET protocol and linear genomic distance. The linear genomic distance is a potential source of noise in the data because regions of the genome closer together in linear genomic distance are on average more likely to have more frequent interactions by chance than regions of the genome farther away from each other, as observed in Hi-C data. We assume that these two sources of noise are independent Poisson processes from each other (since we are measuring the interaction frequency through PET counts), which appears to be a valid assumption in practice (Phanstiel et al., 2015). With this in mind, the parameters within the model were as follows:

P_i – the count of DNA mapped sequences/reads measured at position i, where $i \in \{1..N\}$ and N is the total number of positions measured

Z_i— a latent variable having a value of either 0 or 1 measuring whether the measurement in P_i came from a technical artifact (0) or *in vivo* biology (1)

G_{ij} – a latent variable, where $j \in \{0,1\}$, measuring the number of counts observed for sample *i* if were part of component *j*

B_{ij} – a latent variable measuring the number of counts observed for sample *i* if were part of component *j* as a function of the bias due to the genomic distance that sample *i* spans in the genome (where the distance is assumed to be a constant d_i for that sample *i*)

R_{ij} – a latent variable measuring sum of the two independent processes G_{ij} and B_{ij} for sample *i* if were part of component *j*

λ_j – a parameter describing the mean of latent variable $G_{.j}$ for all samples, and we guarantee that $\lambda_1 > \lambda_0$to maintain identifiability of each component

$\nu_j(d)$ – a parameter describing the mean of the latent variable $B_{.j}$ at distance *d*

w_{ij} – a parameter describing the binomial probability that sample *I* is part of component *j*

a_i, b_i – a set of constants on the prior distribution of w_{i1} to adjust our prior belief in sample *i* based on our understanding of the biology we have already validated in lab

More specifically, the model is parametrized as follows:

$$\lambda_j \sim Gamma(1,1)$$

$$w_{i1} \sim Beta(a_i, b_i)$$

$$G_j|\lambda_j \sim Poisson(\lambda_j)$$

$$B_{ij}|\nu_{j,}, d_i \sim Poisson(\nu_j|d_i)$$

$$R_{ij} = G_{ij} + B_{ij}|\lambda_j, \nu_j, d_i \sim Poisson(\lambda_j + \nu_j|d_i)$$

$$P_i|R_{ij}, \lambda_j, \nu_j, d_i = \sum_{j \in \{0,1\}} w_{ij} * R_j$$

$$P_i|Z_i = z_i, \lambda_{z_i}, \nu_{z_i}, d_i \sim Poisson\big(\lambda_j + \nu_j\big|d_i\big)$$

$$w_{i1}|\, Z_i = z_i, P_i \sim Beta(a_i + z_i, b_i + (1 - z_i))$$

And $w_{i0} = 1 - w_{i1}$.

The distribution of the parameters and hyperparameters were simulated by Markov Chain Monte Carlo (MCMC) using either Gibbs sampling or the Metropolis-Hastings algorithm as appropriate. To speed up the simulation between $G_{.j}$ and B_{ij}, the $G_{.j}$ parameter is updated first. Then the B_{ij} parameters is updated using the difference between the PETs for $G_{.j}$ and the number of PETs observed for each contact according

to the component they are assigned to in that iteration. Additionally, the mean of G_0 is enforced to be less than the mean of G_1, although in practice the mean of G_1 was always strictly greater than the mean of G_0 during the MCMC run so this was never a problem.

The parameter $v_j(d)$ is the mean of the Poisson process estimating the biological bias from the linear genomic distance between the two ends of the putative contact as a function of this distance. To simplify processing, we estimated this function at each iterative using a smoothed cubic spline regression for putative contacts within group *j*. This approximation worked well by generating trends consistent with the power-law decay observed in Hi-C data sets.

The priors $a_{\cdot i}$ and $b_{\cdot i}$ are set to be minimally informative as possible. The $a_{\cdot i}$ hyperparameter is the frequency of the number of contacts sharing one of the same anchors that have a strictly lower measured PET count than the putative contact *i*. The $b_{\cdot i}$ hyperparamer is set to be the frequency of putative contacts sharing the same anchor that have strictly higher number of observed PETs linking the anchors plus the ratio of the multiplication of the depth of reads at both anchors of the putative contact divided by the median depth across all putative contacts floored at 0. We found setting the priors with a non-informative Beta distribution (i.e., $Beta(1,1)$) would also generally call the same *in vivo* interactions but call many more interactions from the putative contacts, where we believed many more of these were artifacts. Hence, we found this minimally informative prior to be more useful for us biologically.

Each run of *origami-analysis* was for 1,000 iterations with a 100 step burn-in period. We chose this number of iterations because the model tended to converge fairly quickly given the complexity of these ChIA-PET data sets. The output of origami is the estimated posterior probability that the putative contact arose from the distribution estimated for the second mixture component, which is assumed to model *in vivo* interactions within the ChIA-PET experiment. Accordingly, putative contact with a posterior probability closer to 1 are believed to be more likely to be *in vivo* interactions.

<u>CTCF motif analysis</u>
CTCF motifs were called in the human and mouse genomes (using hg19 and mm9 assemblies, respectively) using *fimo* (Grant et al., 2011). The CTCF motif from the JASPAR CORE 2014 database was used. The *fimo* p-value threshold was set to 1e-2 and the max-stored-scores parameter set to 100000000. To rank the importance of individual CTCF motifs, the motifs within the targeted CTCF peak upstream of *MYC* were ranked by their score within the score column in the GFF output of fimo.

<u>Identification of genes with a putative enhancer-docking site</u>
Genes with a putative enhancer-docking site were identified by filtering the list of all 26,801 annotated genes down to those that were expressed to a level of 1 transcript per million based on available expression data for HCT-116, Jurkat and K562 cells (Table S4). CTCF ChIP-seq was then used to filter for genes that had a CTCF peak within 2.5

kilobase of an active TSS. Interaction data was then used to score if active TSSs with CTCF occupancy were interacting with an active enhancer. Enhancers were called using H3K27Ac data from each corresponding cell line. TSSs that qualify these conditions are reported in Table S2. Loop anchors from the interaction data that were overlapping with enhancers were used to identify all CTCF ChIP-seq peaks that could be involved in the anchoring on the enhancer side of the loops. The enhancer-promoter loop anchors, gene name, transcriptions start site, CTCF peaks, the JASPAR score off the strongest motif and the orientation of the strongest motif are included Table S6.

Gene Ontology (GO) analysis

We took all putative enhancer-docking site genes per cell type. We ran each gene set through the GO analysis against all GENCODE genes to find enriched biological processes. Using the topGO and qvalue Bioconductor packages, we tested for enriched terms using Fischer's exact test and correcting for multiple hypothesis testing by converting all p-values into q-values via pFDR. We corrected for multiple hypothesis testing by grouping all biological process, molecular function, and cellular function terms independently. We selected all enriched GO terms with a q-value less than .1 (10% FDR) for further analysis.

Supplementary References

Bray, N.L., Pimentel, H., Melsted, P., and Pachter, L. (2016). Near-optimal probabilistic RNA-seq quantification. Nat. Biotechnol. *34*, 525–527.

Dixon, J.R., Selvaraj, S., Yue, F., Kim, A., Li, Y., Shen, Y., Hu, M., Liu, J.S., and Ren, B. (2012). Topological domains in mammalian genomes identified by analysis of chromatin interactions. Nature *485*, 376–380.

Ghavi-Helm, Y., Klein, F.A., Pakozdi, T., Ciglar, L., Noordermeer, D., Huber, W., and Furlong, E.E.M. (2014). Enhancer loops appear stable during development and are associated with paused polymerase. Nature *512*, 96–9100.

Grant, C.E., Bailey, T.L., and Noble, W.S. (2011). FIMO: Scanning for occurrences of a given motif. Bioinformatics *27*, 1017–1018.

Hnisz, D., Abraham, B.J., Lee, T.I., Lau, A., Saint-André, V., Sigova, A.A., Hoke, H.A., and Young, R.A. (2013). Super-enhancers in the control of cell identity and disease. Cell *155*, 934–947.

Kent, W.J., Sugnet, C.W., Furey, T.S., Roskin, K.M., Pringle, T.H., Zahler, A.M., and Haussler, D. (2002). The human genome browser at UCSC. Genome Res. *12*, 996–1006.

Langmead, B., Trapnell, C., Pop, M., and Salzberg, S.L. (2009). Ultrafast and memory-efficient alignment of short DNA sequences to the human genome. Genome Biol. *10*, R25.

Lee, T., Johnston, S., and Young, R. (2006). Chromatin immunoprecipitation and

microarray-based analysis of protein location. Nat. Protoc. *1*, 729–748.

Li, H., Handsaker, B., Wysoker, A., Fennell, T., Ruan, J., Homer, N., Marth, G., Abecasis, G., and Durbin, R. (2009). The Sequence Alignment/Map format and SAMtools. Bioinformatics *25*, 2078–2079.

Ling, Q., Huang, W., Baldwin, A., and Jarvis, P. (2012). Chloroplast biogenesis is regulated by direct action of the ubiquitin-proteasome system. Science *338*, 655–659.

Lovén, J., Hoke, H.A., Lin, C.Y., Lau, A., Orlando, D.A., Vakoc, C.R., Bradner, J.E., Lee, T.I., and Young, R.A. (2013). Selective inhibition of tumor oncogenes by disruption of super-enhancers. Cell *153*, 320–334.

Mumbach, M.R., Rubin, A.J., Flynn, R.A., Dai, C., Khavari, P.A., Greenleaf, W.J., and Chang, H.Y. (2016). HiChIP: efficient and sensitive analysis of protein-directed genome architecture. Nat. Methods *13*, 919–922.

Nagano, T., Varnai, C., Schoenfelder, S., Javierre, B.-M., Wingett, S., and Fraser, P. (2015). Comparison of Hi-C results using in-solution versus in-nucleus ligation. Genome Biol. *16*, 175.

Phanstiel, D.H., Boyle, A.P., Heidari, N., and Snyder, M.P. (2015). Mango: A bias-correcting ChIA-PET analysis pipeline. Bioinformatics *31*, 3092–3098.

Quinlan, A.R., and Hall, I.M. (2010). BEDTools: A flexible suite of utilities for comparing genomic features. Bioinformatics *26*, 841–842.

Ran, F.A., Hsu, P.D., Wright, J., Agarwala, V., Scott, D.A., and Zhang, F. (2013). Genome engineering using the CRISPR-Cas9 system. Nat. Protoc. *8*, 2281–2308.

Rao, S.S.P., Huntley, M.H., Durand, N.C., Stamenova, E.K., Bochkov, I.D., Robinson, J.T., Sanborn, A.L., Machol, I., Omer, A.D., Lander, E.S., et al. (2014). A 3D map of the human genome at kilobase resolution reveals principles of chromatin looping. Cell *159*, 1665–1680.

Sanjana, N.E., Shalem, O., and Zhang, F. (2014). Improved vectors and genome-wide libraries for CRISPR screening. Nat. Methods *11*, 783–784.

Shalem, O., Sanjana, N.E., Hartenian, E., Shi, X., Scott, D. a, Mikkelsen, T.S., Heckl, D., Ebert, B.L., Root, D.E., Doench, J.G., et al. (2014). Genome-scale CRISPR-Cas9 knockout screening in human cells. Science *343*, 84–87.

Vidigal, J.A., and Ventura, A. (2015). Rapid and efficient one-step generation of paired gRNA CRISPR-Cas9 libraries. Nat. Commun. *6*, 8083.

Wang, Y., Zhang, B., Zhang, L., An, L., Xu, J., Li, D., Choudhary, M.N.K., Li, Y., Hu, M., Hardison, R., et al. (2017). The 3D Genome Browser: a web-based browser for visualizing 3D genome organization and long-range chromatin interactions. bioRxiv.

Weintraub, A.S., Li, C.H., Zamudio, A. V, Sigova, A.A., Hannett, N.M., Day, D.S., Abraham, B.J., Cohen, M.A., Nabet, B., Buckley, D.L., et al. (2017). YY1 Is a Structural Regulator of Enhancer-Promoter Loops. Cell *171*, 1573–1588.e28.

van de Werken, H.J.G., De Vree, P.J.P., Splinter, E., Holwerda, S.J.B., Klous, P., De Wit, E., and De Laat, W. (2012a). 4C technology: Protocols and data analysis (Elsevier Inc.).

van de Werken, H.J.G., Landan, G., Holwerda, S.J.B., Hoichman, M., Klous, P., Chachik, R., Splinter, E., Valdes-Quezada, C., Öz, Y., Bouwman, B.A.M., et al. (2012b). Robust 4C-seq data analysis to screen for regulatory DNA interactions. Nat Methods *9*, 969–972.

Whyte, W.A., Orlando, D.A., Hnisz, D., Abraham, B.J., Lin, C.Y., Kagey, M.H., Rahl, P.B., Lee, T.I., and Young, R.A. (2013). Master transcription factors and mediator establish super-enhancers at key cell identity genes. Cell *153*, 307–319.

Zhang, Y., Liu, T., Meyer, C.A., Eeckhoute, J., Johnson, D.S., Bernstein, B.E., Nussbaum, C., Myers, R.M., Brown, M., Li, W., et al. (2008). Model-based analysis of ChIP-Seq (MACS). Genome Biol. *9*.

Zhou, X., Maricque, B., Xie, M., Li, D., Sundaram, V., Martin, E. a, Koebbe, B.C., Nielsen, C., Hirst, M., Farnham, P., et al. (2011). The Human Epigenome Browser at Washington University. Nat. Methods *8*, 989–990.
